# Single‐cell RNA sequencing reveals the CRTAC1 ^+^ population actively contributes to the pathogenesis of spinal ligament degeneration by SPP1 ^+^ macrophage

**DOI:** 10.1111/acel.14320

**Published:** 2024-08-19

**Authors:** Yulong Tang, Dachun Zhuo, Yuexin Yu, Weilin Pu, Yanyun Ma, Yuting Zhang, Yan Huang, Qing Zhang, Kunhai Tang, Chen Meng, Di Yang, Lu Bai, Dongyi He, Li Jin, Hejian Zou, Huji Xu, Qi Zhu, Jiucun Wang, Yuanyuan Chen, Jing Liu

**Affiliations:** ^1^ Shanghai Key Laboratory of Vascular Lesions and Remodeling, Shanghai Pudong Hospital, Fudan University Pudong Medical Center, and Human Phenome Institute, Zhangjiang Fudan International Innovation Center Fudan University Shanghai China; ^2^ State Key Laboratory of Genetic Engineering School of Life Science, Fudan University Shanghai China; ^3^ Greater Bay Area Institute of Precision Medicine (Guangzhou), School of Life Sciences, Fudan University Guangzhou China; ^4^ Ministry of Education Key Laboratory of Contemporary Anthropology, Department of Anthropology and Human Genetics School of Life Sciences, Fudan University Shanghai China; ^5^ Research Unit of Dissecting the Population Genetics and Developing New Technologies for Treatment and Prevention of Skin Phenotypes and Dermatological Diseases (2019RU058) Chinese Academy of Medical Sciences Beijing China; ^6^ Institute of Arthritis Research, Shanghai Academy of Chinese Medical Sciences, Guanghua Integrative Medicine Hospital Shanghai China; ^7^ Department of Rheumatology Shanghai Guanghua Hospital of Integrated Traditional Chinese and Western Medicine Shanghai China; ^8^ Division of Rheumatology Huashan Hospital, and Institute of Rheumatology, Immunology and Allergy, Fudan University Shanghai China; ^9^ Department of Rheumatology and Immunology Changzheng Hospital, Naval Medical University Shanghai China; ^10^ Orthopedic Department Shanghai Sixth People's Hospital, Shanghai Jiaotong University Affiliated Shanghai China; ^11^ Orthopaedic Department People's Hospital of Shigatse City Shigatse China

**Keywords:** degenerative lumbar spinal stenosis, immune regulation, ligament degeneration, low back pain, scRNA‐seq

## Abstract

Degenerative spinal stenosis is a chronic disease that affects the spinal ligaments and associated bones, resulting in back pain and disorders of the limbs among the elderly population. There are few preventive strategies for such ligament degeneration. We here aimed to establish a comprehensive transcriptomic atlas of ligament tissues to identify high‐priority targets for pharmaceutical treatment of ligament degeneration. Here, single‐cell RNA sequencing was performed on six degenerative ligaments and three traumatic ligaments to understand tissue heterogeneity. After stringent quality control, high‐quality data were obtained from 32,014 cells. Distinct cell clusters comprising stromal and immune cells were identified in ligament tissues. Among them, we noted that collagen degradation associated with CTHRC1^+^ fibroblast‐like cells and calcification linked to CRTAC1^+^ chondrocyte‐like cells were key features of ligament degeneration. SCENIC analysis and further experiments identified ATF3 as a key transcription factor regulating the pathogenesis of CRTAC1^+^ chondrocyte‐like cells. Typically, immune cells infiltrate localized organs, causing tissue damage. In our study, myeloid cells were found to be inflammatory‐activated, and SPP1^+^ macrophages were notably enriched in degenerative ligaments. Further exploration via CellChat analysis demonstrated a robust interaction between SPP1^+^ macrophages and CRTAC1^+^ chondrocyte‐like cells. Activated by SPP1, ATF3 propels the CRTAC1/MGP/CLU axis, fostering ligament calcification. Our unique resource provides novel insights into possible mechanisms underlying ligament degeneration, the target cell types, and molecules that are expected to mitigate degenerative spinal ligament. We also highlight the role of immune regulation in ligament degeneration and calcification, enhancing our understanding of this disease.

## Graphical abstract

This study demonstrated the pathogenesis of ligament degeneration. Key features of degenerative ligaments include collagen degradation, associated with CTHRC1+ fibroblast‐like cells, and calcification, linked to CRTAC1^+^ chondrocyte‐like cells. Within the immune compartment of degenerative ligaments, myeloid cells exhibit inflammatory activation, with a significant enrichment of SPP1+ macrophages. These macrophages actively interact with CRTAC1^+^ chondrocyte‐like cells through the key mediating transcription factor ATF3, thereby driving the CRTAC1/MGP/CLU axis and promoting ligament calcification.

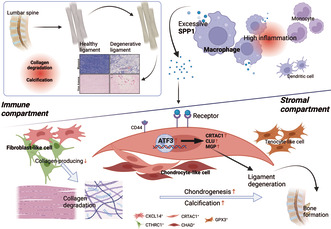

**Abbreviations**
cDC: conventional dendritic cell
CSI: connection specificity index
DEGs: differentially expressed genes
DSS: degenerative spinal stenosis
ECM: extracellular matrix
GO: Gene Ontology
GRN: gene regulatory network
Hh: hedgehog
HVGs: highly variable genes
IF: immunofluorescence
IHC: immunohistochemistry
JIA: juvenile idiopathic arthritis
L‐R: ligand‐receptor
PBS: phosphate buffer saline
PCs: principal components
PVDF: polyvinylidene difluoride
RSS: regulon specificity score
SCENIC: Single‐Cell Regulatory Network Inference and Clustering
scRNA‐seq: single‐cell RNA sequencing
SDS‐PAGE: sodium dodecyl sulfate polyacrylamide gel electrophoresis
siRNA: small interfering RNA
SpA: spondyloarthritis
TBST: Tris‐buffered saline with Tween 20
TF: transcription factor
TSS: transcription start site
UMAP: Uniform manifold approximation and projection
UMIs: unique molecular identifiers

## 1 INTRODUCTION

Degenerative spinal stenosis (DSS) is the most common disease among the world's aging population. It typically affects the cervical and lumbar spine, causing focal and radicular pain, claudication, limited mobility, and a significant global socio‐economic burden (Katz et al., 2022; Knezevic et al., 2021; Schneider et al., 2021; GBD 2021 Low Back Pain Collaborators, 2023). In the aging population, this pathology is most frequently associated with spinal surgery. The radiological manifestations of DSS are degeneration of the three‐joint complex, ligamentum flavum thickening, and osteophyte formation, which ultimately decrease the space of the neurovascular elements (Abbas et al., 2023). One study found that up to 30% of elderly individuals (>55 years) have at least moderate radiological stenosis without symptoms (Tong et al., 2006); these patients may have worse outcomes than those with obvious symptoms if the pathological process cannot be halted. However, the molecular mechanisms associated with osteophyte formation remain largely elusive, and there are no effective drugs to ameliorate or reverse the process of osteophyte formation.

In recent years, single‐cell RNA sequencing (scRNA‐seq) has been widely used to explore biological functions and identify new cell subtypes in various organs and tissues (Li et al., 2023; Perez et al., 2022; Wang et al., 2023). scRNA‐seq has been used to deconvolute tendon tissues, revealing key cellular and molecular phenotypes that drive pathogenesis (Akbar, MacDonald, et al., 2021; Garcia‐Melchor et al., 2021; Ying, 2023; Zhao, Liang, et al., 2022). Increasing scRNA‐seq‐based evidence has shown that inflammation and immune regulation are important in the biological functions of entheses (ligaments and tendons) (Millar et al., 2017). Key inflammatory mediators, such as cytokines, nitric oxide, prostaglandins, and lipoxins, play crucial roles in modulating tendinopathy‐related changes in the extracellular matrix (ECM) (Akbar, Crowe, et al., 2021; Millar et al., 2015, 2017). Pathogenic processes of the enthesis involve various cell types, including chondrocytes, fibroblasts, tenocytes, endothelial cells, and immune cells (Millar et al., 2021). The hedgehog (Hh) signaling pathway and *SPARC* (OSN) genetic predisposition factors for ligament pathologies (Feng et al., 2020; Lim et al., 2021; Trombetta‐eSilva et al., 2015). Several studies have demonstrated the involvement of macrophages, T cells, and novel cell populations in a SpA mouse model (Dai et al., 2020; Nakamura et al., 2021; Regan‐Komito et al., 2020; Venken et al., 2023; Yang et al., 2018; Yi et al., 2023; Zhong et al., 2018). In human entheses, myeloid cells can secrete interleukin (IL)‐23, inducing T cell production of IL‐17 (Cuthbert et al., 2019). M2 macrophages are also activated in juvenile idiopathic arthritis (JIA) patients (Schulert et al., 2016).

Previous studies have primarily focused on tendinopathy related to frozen shoulder and in the patellar and Achilles tendons. Challenges remain in determining the key pathogenic cell types and molecular patterns during degenerative enthesis pathologies, especially in age‐related spinal ligament degeneration. An in‐depth exploration of interactions between immune cells and stromal cells in the spinal ligament tissue is necessary to shed light on possible treatments. To this end, we here utilized scRNA‐seq to decipher the composition of stromal cells and infiltrated immune cells, allowing construction of a comprehensive spinal ligament tissue and associated microenvironment cell atlas. This unique resource was designed to enable identification of specific cell types involved in DSS; to provide potential therapeutic targets for spinal degenerative diseases; and to clarify the pathogenesis of other musculoskeletal disorders, including osteoarthritis and ankylosing spondylitis.

## 2 METHODS

### 2.1 Participants

We enrolled six patients with degenerative lumbar stenosis and three patients with trauma for this study. Detailed patient information is provided in Table S1. All participants provided written informed consent. Approval for this study was granted by the institutional review boards of Shanghai Sixth People's Hospital (2023‐KY‐167(K)), Shanghai Guanghua Hospital of Integrated Traditional Chinese and Western Medicine (2021‐K‐04), and Fudan University (FE22230I).

### 2.2 Sample collection

Tissue samples, including bone (spinous process, facet, and lamina) and ligaments (ligamentum flavum, interspinous ligament, and supraspinal ligament), were obtained during spinal canal decompression surgery. Immediately after extraction, the tissue was preserved in ice‐cold solution (phosphate buffer saline [PBS] containing 1% penicillin and streptomycin and 0.5% bovine serum albumin filtered by a 0.22 μm sterile disposable needle filter) and then transported to the laboratory for cell dissociation.

Surgically obtained tissue underwent three washes with PBS to eliminate impurities such as blood clots. Ligaments were meticulously separated from bone fragments, minced into approximately1 mm^2^ pieces with scalpels and scissors, and digested in a 37°C water bath for 1–2 h using a digestion solution (α‐MEM supplemented with 2 mg/mL type II Collagenase, 1 mg/mL DNase I, and 0.5 mg/mL dispase II) with consistent gentle shaking.

The solution buffer was monitored for cell counting by microscopy every 15 min until the isolated cells reached a total count of 10^8^ cells. Subsequently, the single‐cell suspension passed through 100‐μm and 40‐μm cell strainers (BD Falcon) sequentially, followed by centrifugation at 400 × g for 5 min at 4°C. The cells were resuspended in 200 μL FACS buffer solution (sterilized PBS supplemented with 1% BSA) and 1.8 mL red blood cell lysis buffer on ice for 8 min. After centrifugation at 400 × g for 5 min at 4°C, the cells were resuspended in 100 μL FACS buffer or α‐MEM complete medium for fluorescence‐activated cell sorting or cell culture, respectively.

### 2.3 Fluorescence‐activated cell sorting

The cell suspension was incubated with Fixable Viability Dye eFlour 506 (BV510, ThermoFisher, USA) for 15 min at 4°C in the dark, washed by PBS, and then incubated with CD45 (PerCP‐Cy5.5, BD Biosciences, USA) for 15 min at 4°C in the dark. Fluorescence‐activated cell sorting was performed using BD FACS Aria III (BD Biosciences). Debris and doublets were removed, and live cells expressing CD45 were sorted following the gating strategy shown in supplemental Figure S1. CD45^+^ live cells and CD45^−^ live cells were collected into two independent cell collection tubes, respectively.

### 2.4 Single‐cell RNA sequencing by 10X genomics

Due to potential selection bias inherent in the 10X Genomics platform for constructing single‐cell cDNA libraries for sequencing, we pooled CD45^+^ cells and CD45^−^ cells at a ratio of 3:7 into a new cell suspension before applying it to the 10X Genomics system. Library preparation utilized Gel Bead Kit V3 (10X Genomics, Pleasanton, CA) following standard protocols. The resulting single‐cell libraries were sequenced on an Illumina NovaSeq 6000 using paired‐end sequencing mode under default settings.

### 2.5 Processing of scRNA‐seq data

The gene‐barcode matrices were generated using the Cell Ranger toolkit (version 3.1), aligning sequences to the GRCh38 human reference genome and counting unique molecular identifiers (UMIs) for each cell. Quality control and downstream analysis employed the R package Seurat (version 3.1.4) (Hafemeister & Satija, 2019). Initially, cells with mitochondrial gene counts exceeding 10% and UMIs <500 or >5000 were filtered. Potential doublets were identified and removed using the R package DoubletFinder (version 2.0) with default settings. Subsequently, we normalized feature expression for each cell using “LogNormalize” and log‐transformed the results with the NormalizeData function. Batch effects were corrected utilizing the R package Harmony (version 0.1.1). The scaled and batch effect‐corrected expression profiles of all samples were utilized for subsequent analyses.

Top principal components (PCs) were computed based on gene expression profiles of 2000 highly variable genes (HVGs). The optimal number of PCs for further analysis was determined by the PCElbowPlot function. Cell clustering was performed using the FindNeighbors and FindClusters functions, with the optimal resolution identified through a “clustering tree” method. Visualization was achieved using the RunUMAP function. Cell identity foreach cluster was defined based on the expression of well‐known or previously reported cell‐associated markers.

Differentially expressed genes (DEGs) within each subcluster were identified using the FindAllMarkers function in *Seurat*, employing the Wilcoxon rank‐sum test to assess the significance of each gene. Criteria for defining cluster signatures included expression in more than 20% of cells within either the traumatic or degenerative group, or both, with a log_2_FoldChange >1 or <−1 and a Wilcoxon rank‐sum test adjusted *p*‐value < 0.05 (adjusted with false discovery rate for multiple testing).

### 2.6 Trajectory analysis

Trajectory analysis was conducted using the R packages Monocle 2 (version 2.28.0) (Qiu et al., 2017). Ordering genes and variance levels were set as recommended by Monocle 2. The DDRTree function was applied to reduce dimensions with default settings. The differentialGeneTest function in Monocle 2 was used to reveal DEG variance with pseudotime. The URD package was employed to uncover the transition process of macrophages with default settings (Siebert et al., 2019).

### 2.7 Construction of inflammation scores in the scRNA‐seq dataset

Chronic inflammation's potential role in ligament degeneration led us to construct an inflammation module based on the Gene Ontology (GO) database. Module scores were determined using the AddModuleScore function in *Seurat*, with the list of signatures involved in inflammation‐associated pathways detailed in Table S2.

### 2.8 Cell–cell communication

To explore potential interactions between immune and stromal cells, *CellChat* (version 1.6.1) was applied (Jin et al., 2021). Utilizing a manually curated database of literature‐supported ligand‐receptor (L‐R) interactions, based on the law of mass action, it modeled the probability of cell–cell communication through permutation tests. Significant cell‐type‐specific interactions between L‐R pairs (*p* < 0.01) were selected for visualization.

### 2.9 SCENIC and downstream analysis

Single‐Cell Regulatory Network Inference and Clustering (SCENIC) analysis, conducted with the latest version of the pySCENIC pipeline, revealed the gene regulatory network (GRN) in different cell types and clusters. Gene‐motif rankings (500 bp upstream or 100 bp downstream of the transcription start site [TSS]) determined the search space around the TSS. The motif database (mc9nr), comprising 24,453 motifs, was used for RcisTarget and GENIE3 algorithms to infer the GRNs. Regulon analysis was performed to explore the regulons of stromal cells further. The regulon specificity score (RSS) determined the specificity of each predicted regulon for each cell type. Additionally, the connection specificity index (CSI) for all regulons was calculated to reveal connectedness between different regulons. Visualization of the correlation of regulons in different models (CSI > 0.7 as a cut‐off) was achieved using Cytoscape (Shannon et al., 2003).

### 2.10 Immunohistochemistry (IHC) and immunofluorescence (IF) staining

Tissues underwent fixation in 4% paraformaldehyde for 4–6 h, followed by embedding in paraffin. Paraformaldehyde‐fixed paraffin‐embedded sections, cut into 5 μm using a Leica RM2235, were initially deparaffinized in a series of 100% xylene, rehydrated in graded ethanol (100%, 100%, 95%, and 80%), and then briefly washed in distilled water and PBS. Antigen thermal repair was executed with 0.01 M citrate buffer (pH 6.0) or EDTA buffer (pH 9.0 at high pressure). After washing in PBS, sections were treated with 3% hydrogen peroxide‐methanol for 10 min and blocked with normal goat serum for 30 min. Subsequently, sections were incubated overnight with CLU (1:300, Abcam, catalog: ab92548); MGP (1:100, Abcam, catalog: ab192396); CHAD (1:300, Abcam, catalog: ab238551); PCOLCE2 (1:300, Abcam, catalog: ab232721); COL I (1:100, Abcam, catalog: ab260043); COL III (1:100, Abcam, catalog: ab7778); PRG4 (1:600, Novus, catalog: NBP1‐19048) antibody at 4°C. Incubation with goat anti‐rabbit IgG secondary antibody (JACKSON, catalog: 111–035‐003) was followed by color development with DAB solution (Sigma, catalog: D8001). All images were captured using an Open‐field slice scanner (NanoZoomer S210).

For IF staining, tissue samples were fixed, decalcified, sectioned, incubated, and blocked as described. They were then incubated overnight with CRTAC1 (1:300, Abcam, ab254691) or ATF3 (1:100, Abcam, catalog: ab254268) at 4°C. Following washing, sections were incubated with a fluorescein‐conjugated secondary antibody (ZuochengBio, catalog: ZCTD001_5), and nuclei were counterstained with DAPI. Image acquisition was performed using a fluorescence section scanner (PANNORAMIC MIDI II).

### 2.11 Masson's trichrome staining

Paraffin‐embedded tissue sections were deparaffinized in xylene and rehydrated through graded ethanol solutions (100%, 95%) into distilled water. Sections were stained with hematoxylin for 5 min, differentiated in 1% acid alcohol, and washed in distilled water to achieve bluing. After rinsing in distilled water for 5 min, sections were stained with Biebrich scarlet‐acid fuchsin for 15 min, rinsed in 1% acetic acid for 5 min, differentiated in phosphomolybdic acid for 5 min, and rinsed again in 1% acetic acid for 5 min. Sections were stained with aniline blue for 3 min, followed by a final rinse in 1% acetic acid for 5 min. The stained sections were dehydrated through graded ethanol (95% and absolute ethanol), cleared in xylene, and mounted with neutral balsam. Images were captured using a standard light microscope with a digital camera.

### 2.12 Von Kossa staining for calcium salts

Paraffin‐embedded tissue sections were placed in an oven at 60°C for 1 h, then deparaffinized and rehydrated. Sections were rinsed in distilled water for 3 min twice. Von Kossa silver solution was applied, and sections were exposed to ultraviolet light for 15 min. Following this, sections were rinsed in distilled water for 3 min three times, incubated in sodium thiosulfate for 2 min to visualize calcium salts as black granules. Afterward, sections were rinsed in distilled water for 3 min three times, counterstained with nuclear fast red for 5 min, briefly rinsed in distilled water, dehydrated in absolute ethanol, cleared in xylene, and mounted with neutral balsam.

### 2.13 Primary human spinal ligament cell isolation and culture

Primary human spinal ligament cells were isolated as previously described. Single‐cell suspensions were cultivated in α‐MEM (Gibco, catalog: C12571500BT), supplemented with 20% FBS (Gibco, catalog: 10099141C) and 100 U/mL penicillin and streptomycin for 7–14 days at 37°C in a 5% CO_2_ humidified incubator. All experiments were conducted with passages 1–3 for the primary human ligament cells.

### 2.14 Gene silencing and inhibition of ATF3

For transfection experiments, primary human spinal ligament cells from lumbar degenerative stenosis patients were transfected with 40 nM si‐ATF3 mixed with 2 μL Lipofectamine® RNAiMAX transfection reagent (Invitrogen, ThermoFisher Scientific, USA). Transfection efficiency was evaluated at 2 days posttransfection by real‐time PCR and western blotting. Small interfering RNA (siRNA) sequences against ATF3 were designed as reported previously (Hong et al., 2020) and synthesized by Genomeditech (GenePharma, Shanghai, China). The inhibitor targeting ATF3, GW5074 (Selleck, Catalog No. S2872), was used for silencing ATF3 expression as described (Chen et al., 2008).

### 2.15 Recombinant SPP1 protein treatment

For recombinant protein stimulation experiments, primary human ligament cells from patients with traumatic injury were incubated with 200 ng/mL recombinant SPP1 protein (1433‐OP‐050, R&D Systems, USA). Total RNA and protein were extracted after 24‐h and 48‐h incubation, respectively, with or without recombinant SPP1 protein.

### 2.16 Overexpression of ATF3

The target gene ATF3 (RefSeq NM_001030287.4) primer sequence was inserted into the lentivirus vector (PGMLV‐CMV‐MCS‐T2A‐ZsGreen1‐PGK‐Puro) using the seamless cloning method, as illustrated in Figure S2. Lentiviral vectors' plasmids were cotransfected into 293 T cells using HG transgene reagent (Health Gene, catalog: TG‐10012). Supernatants from cells enriched with lentiviral particles were collected and concentrated to yield a high titer of lentiviral concentrate. Viral titers were determined and calibrated in 293 T cells. Traumatic human ligament cells were then infected by lentiviral‐conditioned media with 5 μg/mL polybrene (Sigma‐Aldrich, TR‐1003‐G). Total RNA and protein were extracted after 24‐h and 48‐h incubation, respectively.

### 2.17 Chromatin immunoprecipitation (ChIP)‐PCR

The chromatin was extracted from approximately 1.0 × 10^7^ 293 T cells and treated with 11% formaldehyde to cross‐link proteins with DNA. Cells were lysed with protease inhibitors, sonicated to shear DNA into fragments, and incubated with antibody against ATF3 (Abcam) or IgG (Santa Cruz) overnight. ATF3 proteins were then immunoprecipitated from the precleared lysates. Protein‐DNA complexes were eluted, and cross‐links were reversed. Purified DNA and input genomic DNA were analyzed by PCR using Master Mix (Yeasen). The sequences of primers are as follows: human CLU promoter forward 5′‐ GAGGATCAAACAGCAGCACC′, human CLU promoter reverse 5′‐ CCCTTCCCTTGCCCTAAACA‐3′, human MGP promoter forward 5′‐ GGACTAGGGCACAGGTTTGA′, human MGP promoter reverse 5′‐ TGGGTTTGGTCCACAGACAG‐3′, human CRTAC1 promoter forward 5′‐ TGATCAAAGCGGTGAGGGAG′, human CRTAC1 promoter reverse 5′‐ AGCCGAGCCGATCTTTATCC‐3′.

### 2.18 RNA isolation and real‐time PCR

Total RNA extraction from cultured cells was carried out using TRIzol reagent (Invitrogen, Carlsbad, CA, USA) following the manufacturer's instructions. Reverse transcription was performed using a High‐Capacity cDNA Reverse Transcription Kit (Applied Biosystems, CA, USA). SYBR Premix Ex Taq (TakaRa Biotech, Tokyo, Japan) and primers were mixed with RT‐PCR samples, and quantitative RT‐PCR was conducted using an ABI Prism 7900 Detector System (Applied Biosystems). The housekeeping gene β‐actin served as the internal control, and primer sequences are provided in Table S3.

### 2.19 Western blotting

Cultured cells were lysed at 4°C using radioimmunoprecipitation assay buffer (Solarbio, Shanghai, China) with 1% phenylmethylsulfonyl fluoride protease inhibitor, 1 mmol/L Na_3_VO_4_, and 1 mmol/L NaF. After centrifugation, equal amounts of protein were subjected to sodium dodecyl sulfate polyacrylamide gel electrophoresis (SDS‐PAGE) on a 10% polyacrylamide gel and transferred to a polyvinylidene difluoride (PVDF) membrane (Millipore). The PVDF membrane was blocked with a 5% bovine serum albumin‐containing blocking buffer for 2 h at 25°C. Incubation with primary antibodies CRTAC1 (1:300, Abcam, ab254691); CLU (1:300, Abcam, catalog: ab92548); ATF3 (1:100, Abcam, catalog: ab254268); MGP (1:100, Abcam, catalog: ab192396) was carried out for 12–14 h at 4°C. β‐actin (Servicebio, GB12001) or glyceraldehyde 3‐phosphate dehydrogenase (Proteintech, 60,004‐1‐lg) was employed as an internal control. After three washes with Tris‐buffered saline with Tween 20 (TBST), membranes were incubated with secondary antibodies (horseradish peroxidase‐conjugated goat anti‐rabbit or anti‐mouse immunoglobulin G) for 2 h at 25°C. Following another three washes with TBST, the enhanced chemiluminescence system was used to visualize protein bands. Image‐QuantTL (General Electric Company, CT, USA) and ImageJ software were used for band intensity quantification.

### 2.20 Statistical analysis

All experiments were repeated at least three times, and statistical analyses were conducted using R software. A *p*‐value < 0.05 was considered significant. The two‐tailed Student's *t* test compared two groups, while one‐way analysis of variance with Tukey's post hoc test compared differences between multiple groups. Pathway enrichment analysis utilized *ClusterProfiler* (version 3.16.1). Plots were generated using R packages *Seurat* (version 3.1.4), *Monocle* (version 2.28.0), *CellChat* (version 1.6.1), *pheatmap* (version 1.0.12), or *ggplot2* (version 3.3.2) in R software or GraphPad Prism 7.

## 3 RESULTS

### 3.1 Nineteen distinct cell types in the stenotic and baseline spinal ligament sample

**FIGURE 1:**
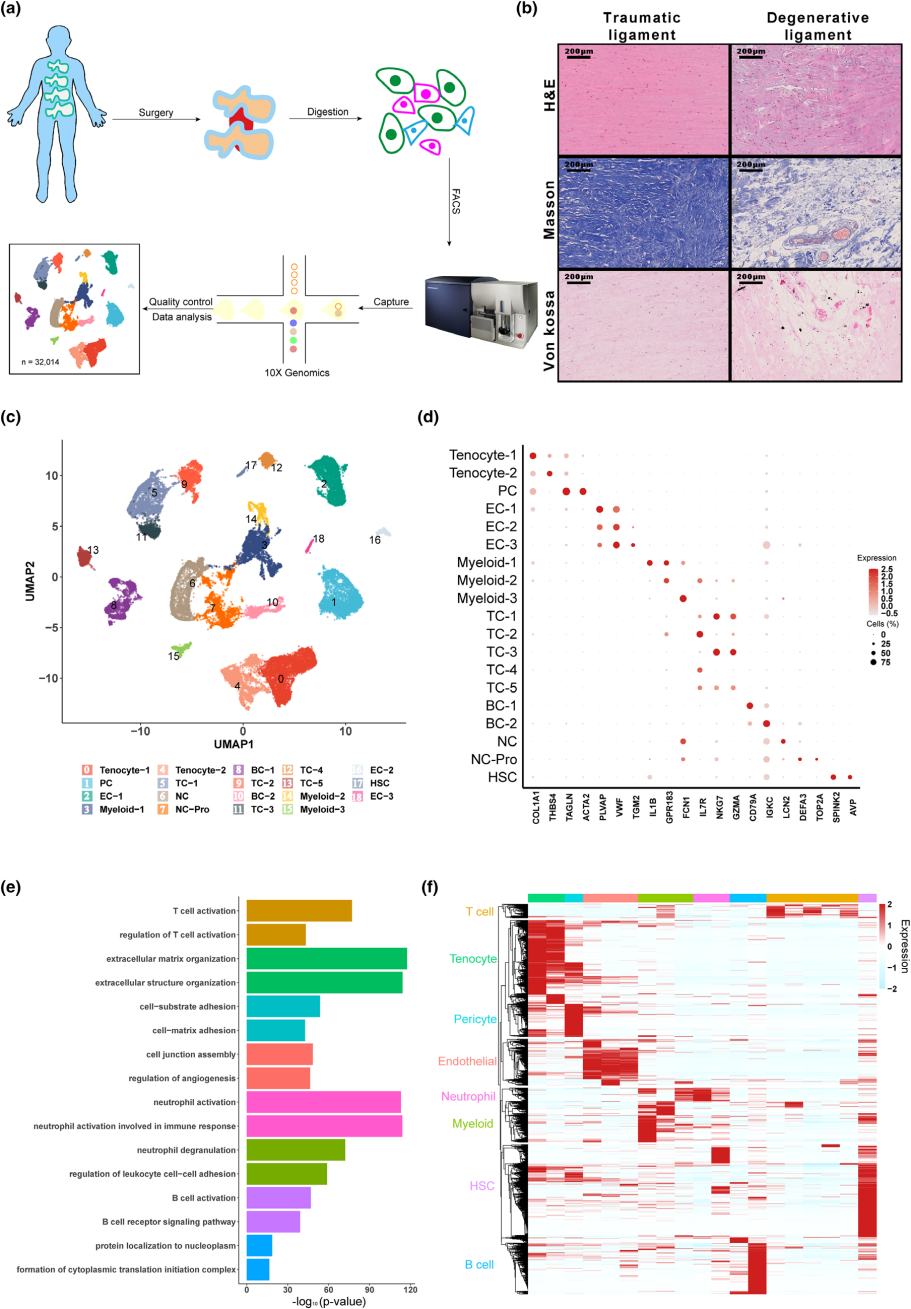
Atlas of cell types in degenerative and non‐degenerative human spinal ligaments. (a) Workflow for sample collection, processing and bioinformatic analysis of spinal ligament tissues in the present study. ScRNA‐Seq was performed using 10X Genomics. (b) H&E staining, Masson staining and Von kossa staining in traumatic (non‐degenerative) and degenerative ligament sections. (c) UMAP clustering of 32,014 high‐quality cells profiled by main cell lineage. PC, pericytes; EC, endothelial cells; TC, T cells; BC, B cells; NC, neutrophils; NC‐pro, proliferation neutrophils. (d) Dot plot of expression level for signature genes in each cell cluster. Color intensity indicates relative expression level and dot size indicates percentage of cells expressing the corresponding markers. (e) GO enrichment analysis of top marker genes for each cell type corresponded well with biological functions. (f) Heatmap of top marker genes and hierarchical clustering of transcriptional patterns in each cluster.

To identify pathological changes in cellular composition associated with spinal ligament degeneration, we obtained fresh tendons and ligaments from DSS patients as well as spinal traumatic injury patients and performed single‐cell RNA sequencing (scRNA‐seq, workflow shown in Figure 1a). Initial morphological analysis showed spinal stenosis samples had consistently more extensive collagen degradation and mineral deposition at the enthesis site than non‐stenosis trauma samples (Figure 1b). ScRNA‐seq of these samples yielded 32,014 total cells for further analysis after filtering for quality control. Uniform manifold approximation and projection (UMAP) analysis to define populations within these samples identified 19 distinct clusters (Figure 1c,d), including two stromal cells clusters (*COL1A1, THBS4*), a pericyte cluster (*TAGLN, ACTA2*), three endothelial cell clusters (*PLVAP, VWF, TGM2*), three myeloid cell clusters (*IL1B, GPR183, FCN1*), five T cell clusters (*NKG7, IL7R, GZMA*), two B cell clusters (*CD79A, IGKC*), a neutrophil cluster (*LCN2*), a proliferative neutrophil cluster (*DEFA3, TOP2A*), and a hematopoietic stem cell cluster (*SPINK2, AVP*). DEG analysis for each cluster revealed cell type‐specific transcriptional programs and pathway enrichment reflective of their respective biological functions (Figure 1e,f). Notably, ECM organization and cell‐matrix adhesion were prominent terms in GO analysis of tenocyte (i.e., stromal) and pericyte clusters, respectively, supporting the accuracy of our analysis.

### 3.2 Identification of ligament stromal subpopulations with potential calcification and degradation function

**FIGURE 2:**
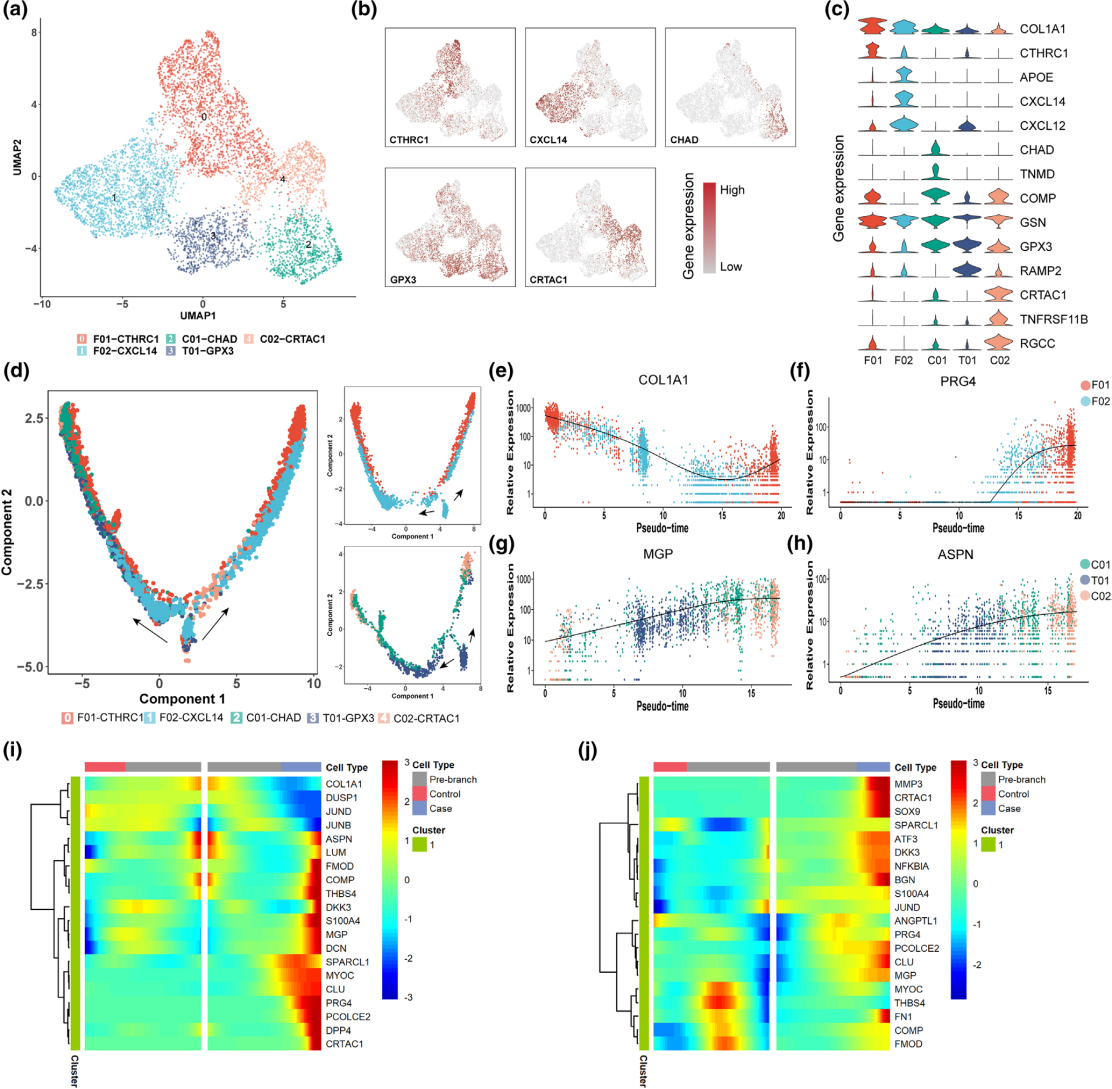
Identifying characteristics of ligament stromal subpopulations. (a) Five stromal cell subpopulations could be distinguished in ligament tissues, including two fibroblast‐like cell types (F01‐CTHRC1 and F02‐CXCL14), two chondrocyte‐like clusters (C01‐CHAD and C02‐CRTAC1) and one tenocyte‐like subpopulation (T01‐GPX3). (b) Feature plots of marker expression in each subpopulations. (c) Top marker genes of each subpopulations. (d) Trajectory analysis of the five subpopulations. (e, f) Expression of cell matrix‐related *COL1A1* and *PRG4* and (g, h) calcification‐related MGP and ASPN in the pseudo‐time trajectory. (i) Key genes in the fibroblast‐like and (j) chondrocyte‐like and tenocyte‐like trajectories.

Since the stromal compartment of ligaments plays an essential role in ECM remodeling, while stromal cell dysfunction contributes to pathological fibrosis and calcification, we performed subclustering analysis to examine heterogeneity specifically within ligament stromal populations. Subsequent UMAP analysis identified five distinct, previously unrecognized ligament stromal subpopulations, including two fibroblast‐like clusters (F01‐CTHRC1 and F02‐CXCL14), two chondrocyte‐like clusters (C01‐CHAD and C02‐CRTAC1), and a tenocyte‐like cluster (T01‐GPX3; Figure 2a, marker genes listed in Table S8).

Among them, the F01 and F02 subpopulations exhibited fibroblast‐like gene signatures, with F01 cells expressing more collagen‐related genes and F02 cells expressing inflammation‐related chemokines and cytokines. The C01 and C02 subpopulations displayed chondrocyte‐like gene signatures, with C01 showing higher expression of *CHAD* (encoding chondroadherin protein) and C02 expressing higher *CRTAC1* (cartilage acidic protein 1), and both expressing elevated *COMP* (cartilage oligomeric matrix protein) compared to other subgroups. The T01 subgroup showed high expression of *GPX3* and *RAMP2*, suggesting a tenocyte‐like phenotype.

Feature plots revealed enrichment for specific gene expression within each cell cluster (Figure 2b). For example, *CXCL14* was predominantly expressed in F02 cells, while *CRTAC1* was primarily expressed in C02 (see Figure 2c for details of primary marker expression in each subgroup). Lineage trajectory analysis to uncover possible dynamic relationships among subpopulations suggested that F02 cells likely differentiate into two F01 cell states that differ between case and control samples (Figure 2d). The transition from F02 to F01 was associated with increased ECM‐related gene expression, such as *COL1* and the cell matrix protein *PRG4* (Figure 2e,f). By contrast, T01 cells could potentially differentiate into C01 or C02 cells (Figure 2d), with C02 cells expressing distinctly higher levels of calcification‐related genes (*MGP* and *ASPN*) than other subpopulations, implying disease‐associated dysfunction (Figure 2g,h). The key genes involved in the trajectory analysis are shown in Figure 2i,j, which indicating the potential ECM degradation and calcification function of the stromal subpopulations.

### 3.3 Impaired collagen expression in CTHRC1 ^+^ fibroblast‐like cells in degenerative ligament

**FIGURE 3:**
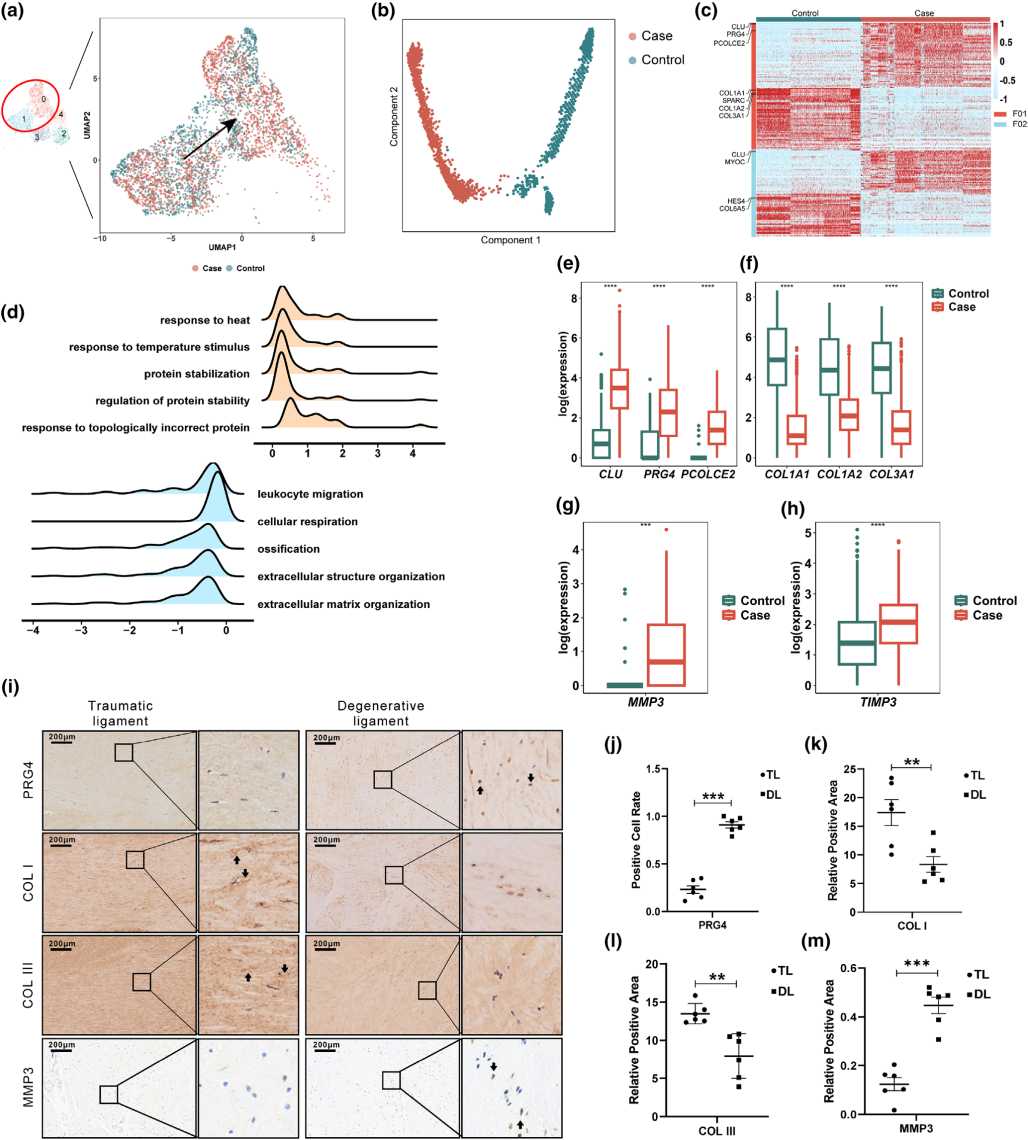
Dysfunction of CTHRC1^+^ fibroblast‐like cells in degenerative ligament. (a) UMAP plot of F01 and F02 fibroblast‐like cells. (b) Trajectory analysis of fibroblast‐like subpopulations. (c) Heatmap of DEGs in fibroblast‐like cells. (d) GSVA enrichment analysis in fibroblast‐like cells. Relative expression levels of (e) *CLU*, *PRG4* and *PCOLCE2*; (f) *COL1A1*, *COL1A2* and *COL3A1*; (g) *MMP3* and (h) *TIMP3* in scRNA‐seq data. (i) IHC staining to detect PRG4, COL1, COL3 and MMP3 in non‐degenerative (left) and degenerative (right) spinal ligament sections with quantitative analysis of PRG4 positive cells, Collagen^+^ area and MMP3^+^ area. (j–m) Quantitative analysis of relative positive area or positive cell rate of target protein in IHC assays. **, *p*‐value < 0.01; ***, *p*‐value < 0.001; ****, *p*‐value < 0.0001.

Since fibroblast‐like cells are the primary collagen‐producing cell type within the stromal compartment of ligament tissue, we conducted subclustering of F01 and F02 fibroblast‐like cells. This analysis revealed that cells from degenerative ligaments were predominantly enriched in F01 (Figure 3a), while pseudo‐time analysis showed obviously distinct trajectories between cells from degenerative and non‐degenerative control ligaments (Figure 3b). Furthermore, DEG analysis showed markedly different transcriptomic profiles in both F01 and F02 subpopulations between the degenerative and control groups. Hierarchical clustering highlighted the dysregulation of several chondrogenesis‐ and ossification‐related genes (*CLU*, *PCOLCE2*, *PRG4*, and *SPARC*) and ECM‐related genes (*COL1A1*, *COL1A2*, *COL3A1*, and *COL6A5*) in the disease state (Figure 3c).

We next conducted GSVA enrichment analysis to further determine which pathways were disrupted between the case and the control groups, which revealed marked enrichment for extracellular structure organization and ECM organization pathways among down‐regulated genes (Figure 3d, Table S4). Most notably, expression of genes related to collagen production was reduced (Figure 3f) while chondrogenesis‐ and calcification‐related genes were elevated (Figure 3e) in degenerative ligaments. In addition, collagen‐degrading enzymes such as *MMP3* (Figure 3g) and *TIMP3* (Figure 3h) were upregulated in degenerative ligaments. Additional validation of these signature genes by IHC staining confirmed that PRG4 and MMP3 expression was enhanced while COL1 and COL3 were downregulated in the degenerative group (Figure 3i–m). These results collectively suggested that pathological collagen degradation might be due to impairment of collagen‐producing CTHRC1^+^ fibroblast‐like cells.

### 3.4 CRTAC1 ^+^ chondrocyte‐like cells show greater capacity for calcification in degenerative group

**FIGURE 4:**
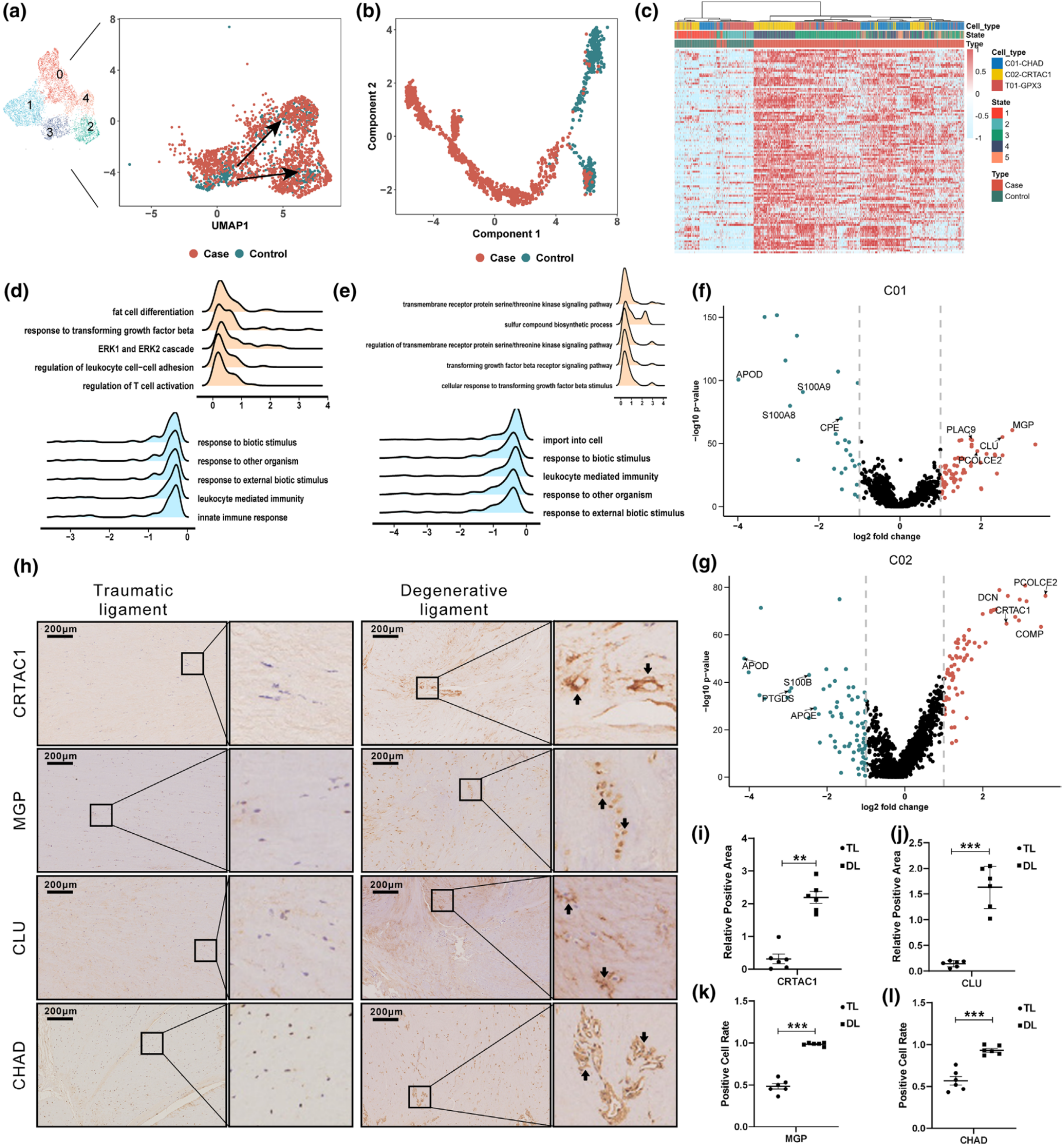
Enhanced function of CRTAC1^+^ chondrocyte‐like cells in degenerative ligament. (a) UMAP plot of chondrocyte‐ (C01 and C02) and tenocyte‐like cells (T01). (b) Pseudo‐time analysis of tenocyte‐to‐chondrocyte trajectories distinguished by chondrogenic and osteogenic transcriptional profiles between degenerative and non‐degenerative ligament samples. (c) Hierarchical clustering of osteogenesis and chondrogenesis‐related DEGs in case and control C01, C02, and T01 cells. (d, e) GSVA enrichment analysis of (d) C01 and (e) C02 subpopulations. (f, g) Volcano plot of DEGs in (f) C01 and (g) C02 cells. (h) IHC detection of CRTAC1, MGP, CLU and CHAD in spinal ligament sections with quantification of relative positive area or positive cell rate. (i‐l) Quantitative analysis of relative positive area or positive cell rate of target protein in IHC assays. **, *p*‐value < 0.01; ***, *p*‐value < 0.001.

As we detected differences in the transcription of calcification‐related genes (e.g., MGP and ASPN) in chondrocyte‐like and tenocyte‐like cells between degenerative and non‐degenerative tissues, we further explored the heterogeneity of C01 and C02 chondrocyte‐like cell clusters, and tenocyte‐like cell cluster T01. Clustering by UMAP showed that the C01 and C02 subpopulations were found in greater abundance than T01 populations in both case and control samples (Figure 4a). Trajectory analysis further illustrated that enhanced expression of osteogenic and chondrogenic genes among degenerative C01 and C02 distinguished the pathological trajectory from that of the physiological state (Figure 4b; Figure S3). Hierarchical clustering of chondrogenesis‐ and osteogenesis‐related genes further verified the differential expression of these genes in the degenerative ligament group, especially in the C02 cluster, compared to the control group (Figure 4c).

GSVA enrichment analysis indicated that up‐regulated genes in C01 cells were primarily enriched in “fat cell differentiation,” “response to transforming growth factor beta,” “ERK1 and ERK2 cascade,” and “biological process of leukocyte related pathways,” while down‐regulated DEGs were mainly enriched in “immune response‐related pathways” (Figure 4d, Table S5). By contrast, up‐regulated DEGs in the C02 cluster were primarily enriched in the “transmembrane receptor serine/threonine kinase signaling pathway,” whereas down‐regulated genes were mainly enriched in “leukocyte‐mediated immunity” (Figure 4e, Table S6). DEG analysis in the C01 and C02 clusters showed that both cell types in degenerative ligament tissue had characteristic upregulation of osteocalcin matrix genes associated with ectopic tissue calcification, such as *MGP*, *PCOLCE2*, and *COMP*, matrix proteoglycans, such as *DCN*, and endochondral‐osteogenesis protein, *CRTAC1*, especially in C02 cells (Figure 4f,g). Validation of these DEGs by in situ IHC staining of ligament sections showed degenerative ligament had greater numbers of positive cells and larger positive areas of CRTAC1, MGP, CLU, and CHAD compared to non‐degenerative controls (Figure 4h–l). These results suggested that calcification of degenerative ligament may be due to activity of CRTAC1^+^ cells, while pathway enrichment implied a role of immune response in regulating these pathological changes.

### 3.5 ATF3 was the key transcription factor (TF) regulating stromal ligament cell pathogenesis

**FIGURE 5:**
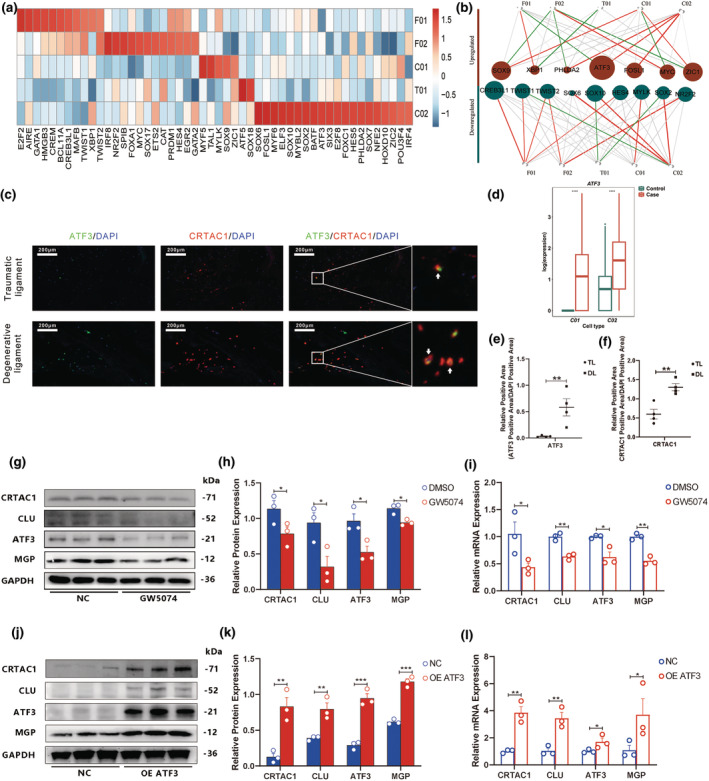
The transcription factor ATF3 regulated pathogenesis in ligament tissues. (a) Expression of selected transcription factors among five stromal cell subclusters. (b) Key transcription factor regulatory network in the five subclusters. (c) Immunofluorescent staining of ATF3 and CRTAC1 in ligament tissues. (d) ATF3 expression among chondrocyte‐like cells in ligament tissues. (e, f) Quantification of the relative positive areas shown in c. (g–i) Western blot analysis (g, h) and quantitative PCR (qPCR) assay (i) of control cultured primary degenerative ligament cells and those treated with the ATF3 inhibitor GW5074. (j–l) Western blot analysis (j, k) and qPCR assay (l) of control cultured primary traumatic ligament cells and those transfected with lentivirus to overexpress ATF3. *, *p*‐value < 0.05; **, *p*‐value < 0.01; ***, *p*‐value < 0.001.

To elucidate the regulatory factors associated with the five stromal cell subgroups, we performed SCENIC analysis to reveal the primary regulatory TFs associated with each group (Figure 5a). Several TFs were activated in CRTAC1^+^ chondrocytes, including *SOX6*, *SOX10*, *ELF3*, and *ATF3*. To identify critical TFs that regulated pathological changes in stromal ligament cells, we constructed a regulatory network using the results of SCENIC analysis for the five stromal cell types. The results suggested that ATF3 functioned as a hub regulator of C02 activity, implicating ATF3 in ligament degeneration (Figure 5b). Indeed, ATF3 was upregulated in chondrocyte‐like cell populations in degenerative group compared to non‐degenerative group (Figure 5d). IF analysis verified that ATF3 and CRTAC1 were upregulated in degenerative ligament tissue as a whole and were co‐expressed in individual cells (Figure 5c,e,f).

The putative regulatory role of ATF3 in pathological stromal cell changes was further validated with in vitro experiments. Specifically, ATF3 was silenced with targeted siRNA or with the specific inhibitor GW5074. CRTAC1 and the osteogenesis‐related genes CLU and MGP were significantly downregulated at both the protein and mRNA levels among cells with ATF3 silenced using either method (Figure 5g–i; Figure S4A–C). Conversely, CRTAC1, CLU, and MGP were upregulated in lentivirus‐transfected cells overexpressing ATF3 (Figure 5j–l). Furthermore, the interaction of CRTAC1, CLU and MGP with ATF3 was examined by a ChIP‐PCR assay, the results showed a direct interaction of CRTAC1 and MGP with ATF3 (Figure S5). These results suggested that ATF3 was a prominent regulator of ligament stromal cell pathogenesis.

### 3.6 SPP1 ^+^ macrophages may play a pathogenic role in the degenerative ligament

The immune compartment, including resident and infiltrating immune cells, can play a crucial role in the regulation and remodeling of ligament tissue. To characterize inflammatory cell diversity and heterogeneity in the ligament immune compartment, we performed subclustering and pathway enrichment analysis for immune cells identified in our initial cell typing.

**FIGURE 6:**
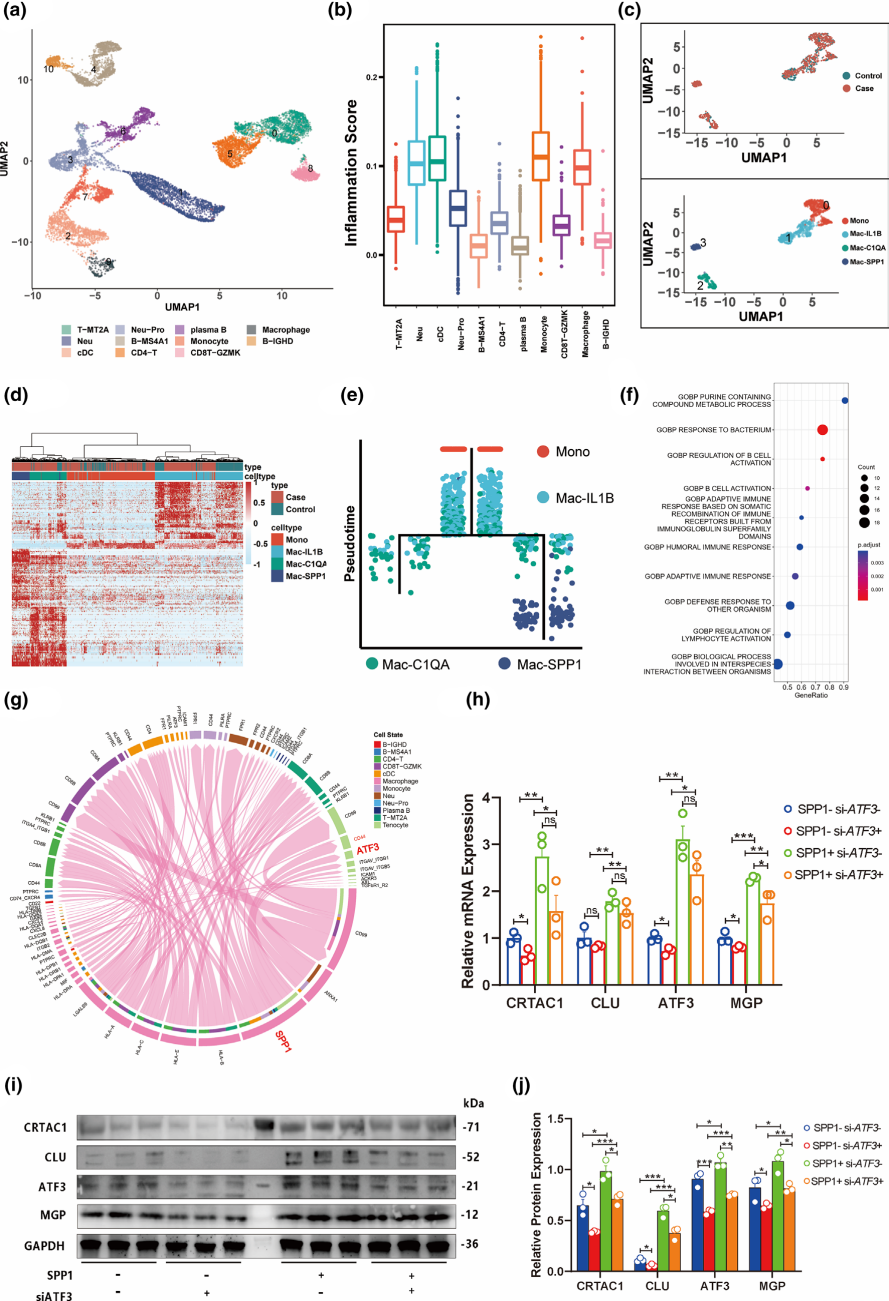
Characterization of ligament immune compartment and possible pathogenic role of SPP1^+^ macrophages. (a) UMAP plot of immune cells in spinal ligament tissues. (b) Inflammation scores of the main immune cell lineages in ligament. (c) UMAP plot of monocytes and macrophages in spinal ligament tissues. (d) Hierarchical clustering of DEGs in monocytes and macrophages. (e) Pseudo‐time analysis of monocyte and macrophage subpopulations. (f) Pathway enrichment analysis for DEGs of macrophages from ligament tissues. (g) Ligand–receptor interactions between macrophages and other cell types. (h–j) Quantitative PCR assays (h) and western blot analysis (i, j) of control cultured primary ligament cells and those treated with short interfering RNA targeting ATF3 and recombinant SPP1. *, *p*‐value < 0.05; **, *p*‐value < 0.01; ***, *p*‐value < 0.001.

This subclustering indicated that the ligament immune compartment mainly comprised T cells, neutrophils, plasma B cells, macrophages, proinflammatory neutrophils, B cells, monocytes, cDC, CD4^+^ T cells, and CD8^+^ T cells (Figure 6a, marker genes listed in Table S9). To assess the activation status of inflammatory signaling in these immune cells, we used a scoring index based on enrichment with genes in inflammatory pathways (listed in Table S2). We found that mainly monocytes and macrophages along a differentiation axis of myeloid cells had the highest inflammation scores in the immune compartment (Figure 6b). Reclustering of monocyte and macrophage subpopulations by UMAP identified four clusters, including monocytes and three macrophage cell types (Mac‐IL1B, Mac‐C1QA, and Mac‐SPP1) (Figure 6c, marker genes listed in Table S10, DEGs of the four cell types listed in Table S11). Further hierarchical clustering revealed that each subpopulation had a distinct transcriptional profile, with Mac‐SPP1 cells found almost exclusively in degenerative spinal ligaments (Figure 6d). Pseudotime trajectory analysis suggested that monocytes first differentiated into Mac‐IL1B, then Mac‐C1QA, and finally Mac‐SPP1 cells (Figure 6e). Pathway enrichment analysis showed that DEGs in macrophages were enriched in “response to the bacterium,” “regulation of B cell activation pathways,” and “defense response to other organisms” (Figure 6f, Table S7).

Given that Mac‐SPP1 populations appear specific to degenerative ligament, we postulated that this cell type could potentially interact with stromal cells, resulting in pathological changes. Based on our above evidence supporting a role of CRTAC1^+^ chondrocyte‐like cells in pathological chondrogenesis and ossification in degenerative ligament, we hypothesized that interactions between Mac‐SPP1 cells and C01/C02 subgroups might result in upregulation of CRTAC1, CLU, and MGP. To test this possibility, we stimulated primary ligament cells isolated from traumatic non‐degenerative ligament samples with exogenous SPP1. Subsequent qPCR and Western blots indicated that CRTAC1, CLU, and MGP were upregulated upon exposure to SPP1 in these ligament cells at both the protein (Figure S6A,B) and mRNA (Figure S6C) levels. These findings strongly supported that SPP1 secreted from Mac‐SPP1 macrophages could induce pathological expression of osteogenic/chondrogenic genes, and implicating SPP1^+^ macrophages (SPP1^+^ Mac) in ligament degeneration.

The results to this point showed that SPP1^+^ macrophages could induce ligament degeneration; that ATF3 was a key regulator of stromal cells; and that chondrocytes were dysregulated by immune cells and the immune response. Thus, we speculated that SPP1^+^ macrophages had pathological roles in stromal cells through interactions with the key mediator ATF3. To assess this hypothesis, we performed a cell–cell communication analysis, which showed that SPP1 and ATF3 interacted with macrophages and tenocytes (Figure 6g, Figure S7, Table S12). This finding was validated with dual si‐ATF3 and recombinant SPP1 treatments in ligament cells derived from traumatic injury patients. The ATF3/CLU/CRTAC1/MGP upregulation triggered by SPP1 was inhibited by si‐ATF3 treatment (Figure 6h–j). To further investigate the interaction of SPP1 and CRTAC1^+^ cells, we performed IF analysis of CD44, a known receptor for SPP1, and CRTAC1. The results showed that a higher number of CRTAC1^+^ cells express CD44 in degenerative ligaments (Figure S8A–C). Additionally, our scRNA‐seq data revealed that CD44 expression was elevated in stromal ligament cells, with the exception of F02, and ITGA5 expression was elevated in C02 (Figure S9). Taken together, these results indicated that secreted SPP1 had a pathological role in ligament degeneration, interacting with stromal cells and regulating the key TF ATF3 to drive pathogenesis.

## 4 DISCUSSION

DSS is a common orthopedic disease that affects spine‐related muscle and bone tissues primarily in the aging population, leading to chronic back pain and a range of clinical symptoms (Hennemann & de Abreu, 2021; Parenteau et al., 2021). Spinal ligament degeneration is a manifestation of DSS that represents a substantial burden to public health and the economy (Ravindra et al., 2018). The etiology of spinal ligament degeneration has remained obscure, but multiple factors are considered contributors, including aging, trauma, overuse, and immune dysregulation (Taylor & Bussières, 2012; Zhao, Xuan, et al., 2022). Previous studies of ligament and tendon pathogenesis have been hampered by limitations in sample types and knowledge of the cell‐type compositions of diseased tissues (Korcari et al., 2023; Yea et al., 2023; Zhang et al., 2023). We here utilized scRNA‐seq to construct a transcriptomic atlas of spinal ligament tissues (cell heterogeneity shown in Figures S11,S12), enabling identification of the pathological mechanisms driving spinal ligament degeneration.

The primary pathological changes in degenerative ligaments are collagen degradation and ligament calcification. The composition of the ECM, especially of collagen fibers, is of critical importance in maintaining ligament and tendon structure and function (Riley, 2008). The collagen composition is known to be altered in tendinopathy tissues, with increases in the type III/type I collagen ratio accompanying mucoid degeneration and neovascularization (Magnusson et al., 2010). We here found that ligament degeneration was characterized by disorganization of collagen fibers and chondroid pre‐osteogenesis. A main collagen‐producing cell cluster, CTHRC1^+^ F01, showed downregulation of collagen‐related genes including *COL1A1*, *COL1A2*, and *COL3A1*. Dynamic changes in these cells drove dysregulation in collagen fibers and in the ECM microenvironment. Genes related to ossification were dysregulated among fibroblast‐like and chondrocyte‐like cell populations. Because fibroblast‐like cells may be the primary drivers of collagen degradation, chondrocyte‐like cells were considered the main pathological cells in ligament degeneration. Markers of chondrocyte hypertrophy and ossification, namely *CLU*, *MGP*, and *PCOLCE2*, were up‐regulated in C01 and C02 populations of degenerative ligaments. CRTAC1^+^ C02 cells were enriched among degenerative ligaments and showed greater differential gene expression between degenerative and control ligaments than other cell types did. CRTAC1 can be used as a biomarker for osteoarthritis severity and progression via plasma proteomics analysis (Szilagyi et al., 2023), demonstrating the role of CRTAC1 and related diverse pathways in chondrocyte pathologies. MGP is a chondrocyte‐specific marker gene for terminal/dedifferentiated chondrocytes, indicating their transition to the skeletal progenitor stages as they convert into osteoblasts (Haseeb et al., 2021). CLU is an articular cartilage chondrocyte marker affected by mechanical loading during growth plate (GP) development and closing (Haseeb et al., 2021). We hypothesize that MGP and CLU regulated bone metabolism in ligament tissues, but this requires further validation.

Immune regulation can strongly influence pathological changes in ligament tissues (Akbar, Crowe, et al., 2021; Cuthbert et al., 2019; Millar et al., 2017, 2021). Infiltrating immune cells can determine ligament repair and degeneration processes. Prior studies have shown that IL‐1β, IL‐18, and IL‐33 are involved in ECM remodeling; that IL‐1β reduces type I collagen production (Archambault et al., 2002); that IL‐33 and IL‐17 increase type III collagen production (Millar et al., 2015), particularly in diseased tendons; that IL‐18 is recruited to immune cells (Millar et al., 2009); and that IL‐6 increases total collagen synthesis and mediates cytokine feedback (Legerlotz et al., 2012). We here found that the COL3/COL1 ratio was significantly increased among stromal degenerative ligament cells (Figure S10A), particularly in the fibroblast‐like cell subpopulation (Figure S10B). Furthermore, macrophages and monocytes had higher inflammation scores in degenerative compared to control ligaments. Macrophages and mast cells accumulate at relatively high levels in a rat model of upper extremity overuse and in a calcaneal tendon overuse model (Pingel et al., 2013). A specific type of macrophage expressing high levels of SPP1 was enriched among degenerative ligaments. SPP1^+^ macrophages have previously been found in various tumor tissues (Luo et al., 2022; Qi et al., 2022; Yu et al., 2023), and SPP1 has a profibrotic role in skin and lung fibrosis (Liu et al., 2022; Papazoglou et al., 2022). Macrophage‐secreted SPP1 was here shown to upregulate the key pathological molecules CRTAC1, ATF3, CLU, and MGP in stromal ligament cells, contributing to ligament degeneration.

SCENIC analysis of the stromal compartment established ATF3 as a hub TF regulating CTHRC1^+^ fibroblast‐like (F01) cells and CRTAC1^+^ chondrocyte‐like (C02) cells. ATF3, which was upregulated in degenerative ligaments, is known to regulate osteogenic differentiation and to contribute to bone formation (Kim et al., 2022). However, it may also serve as an inhibitor of osteogenesis (Fu et al., 2019; Han et al., 2018; Jeong, 2018; Park et al., 2014), and has been shown to modulate calcium signaling in osteoclast differentiation and activity (Jeong et al., 2017). We here showed for the first time that ATF3 was a core mediator of chondrocyte differentiation and a regulator of ligament calcification. CRTAC1, CLU, and MGP were regulated by ATF3, as validated by in vitro ATF3 silencing and overexpression. Further cell–cell communication analysis showed that SPP1 and ATF3 interacted with each other in macrophages and stromal ligament cells. Strikingly, dual SPP1 and si‐ATF3 treatments showed that the CRTAC1/MGP/CLU upregulation triggered by SPP1 stimulation was inhibited by siATF3. Taken together, these results confirmed that macrophage‐secreted SPP1 played a pathological role, interacting with ATF3 to induce CRTAC1/MGP/CLU expression and thus contributing to ligament degeneration.

Several limitations of this study should be noted, including the relatively small sample size. SPP1^+^ Mac enrichment was identified in nine patients; a larger sample size would strengthen the results. The spinal ligament locations analyzed in this study included the five vertebrae of the lumbar spine. Future analysis of varying locations subject to different mechanical loading levels should be conducted for comparison. The inflammation levels of ligaments affected by systematic or localized inflammation could not be addressed in this study, and the origin of the SPP1^+^ Mac was not clarified. Follow‐up functional studies and validations are expected to provide strong evidence of the pathological role of secreted SPP1 and the core mediating TF ATF3, increasing our understanding of spinal degenerative diseases.

## 5 CONCLUSION

Our systematic analysis established a comprehensive transcriptomic atlas of healthy and degenerating spinal ligaments. A pathological macrophage, SPP1^+^ Mac, was identified as a driver of the core mediating factor ATF3, leading to degenerative pathologies. SPP1^+^ Mac and ATF3 are thus novel therapeutic targets for alleviation of spinal degeneration symptoms. These data are a valuable resource, enabling other researchers to clarify the biological mechanisms of spinal or articular diseases, ultimately increasing our understanding of disorders that seriously impact human health and wellbeing.

## AUTHOR CONTRIBUTIONS

Y.T and D.Z performed majority of the experiments. J.L, Y.T and Y.Y analyzed the single cell RNA sequencing data. Y.T and J.L write the original draft. Y.C and W.P provided great help with data analysis and edited the manuscript. Y.M helped with sample collection. Y.Z helped with FACS. Y.H helped with tissue digestion. Q.Z, K.T and L.B helped with some experiments. C.M and D.Y helped with ChIP‐PCR analysis. D.H, L.J, H.Z and H.X provided critical advices to this manuscript. Q.Z and Y.C collected the clinical sample. Y.C, J.W and J.L conceived the idea and J.W supervised this work. All authors contributed to the final version of the manuscript and approved it for publication.

## FUNDING INFORMATION

The study was supported by research grants from the National Natural Science Foundation of China (82001726), CAMS Innovation Fund for Medical Sciences (2019‐I2M‐5‐066), Shanghai Natural Science Foundation (21ZR1454400), Scientific research project of Shanghai Health Commission (202040396), Shanghai Municipal Science and Technology Major Project (2017SHZDZX01), Science and Technology Commission of Shanghai Municipality (19JC1411702), and Interdisciplinary Program of Shanghai Jiaotong University (YG2016MS15).

## CONFLICT OF INTEREST STATEMENT

None declared.

## PATIENT CONSENT FOR PUBLICATION

Not applicable.

## Supporting information


Figure S1.

Table S1.

Table S8.

Table S9.

Table S10.

Table S11.

Table S12.

## Data Availability

The data and the code that support the findings of this study are available on reasonable request from the corresponding author. The scRNA‐Seq datasets presented in this study were deposited in the GEO database under accession no. GSE271018.
