# Supplementary material for: Single‐cell RNA sequencing reveals the CRTAC1 + population actively contributes to the pathogenesis of spinal ligament degeneration by SPP1 + macrophage
Source: Aging Cell. 2024 Aug 19;23(12):e14320. doi: 10.1111/acel.14320 (PMC11634701; doi:10.1111/acel.14320)
Supplement: Supplementary file 1 — Figure S1. [file ACEL-23-e14320-s003.docx]

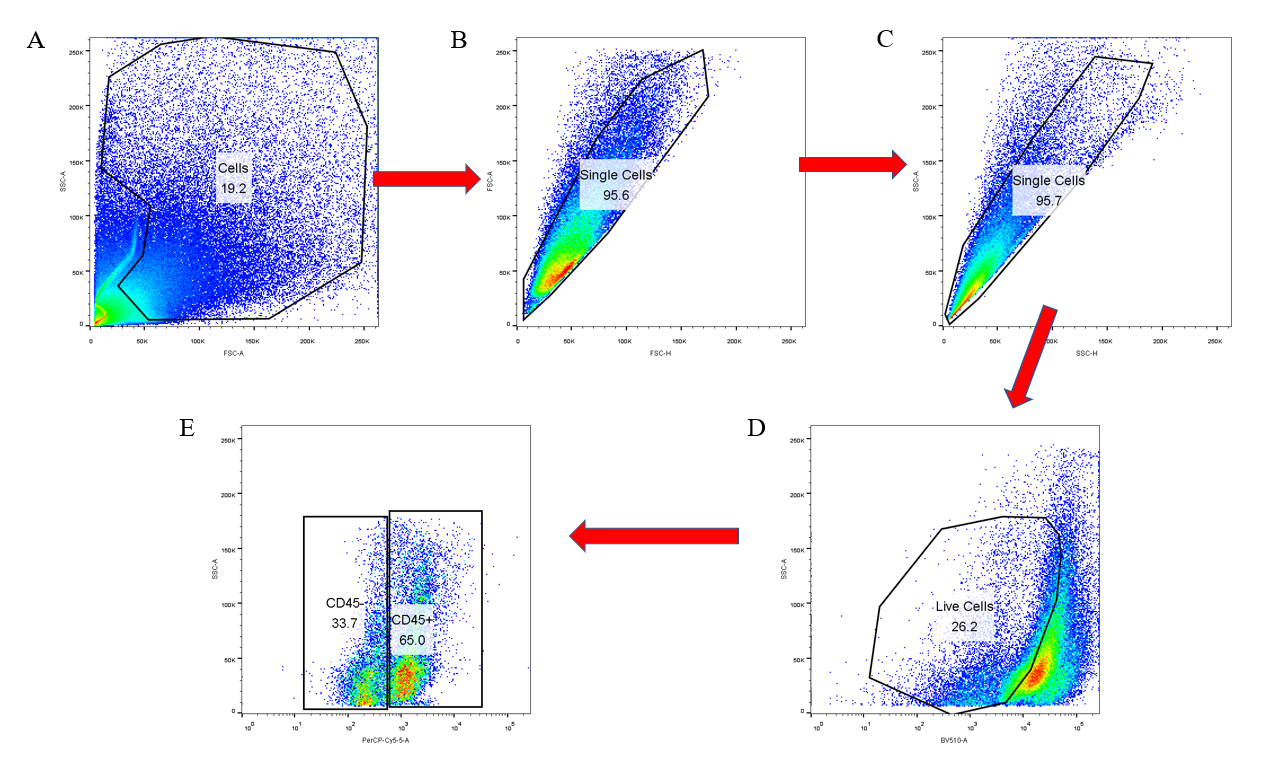


**Figure S1 The gating strategy of fluorescent activated cell sorting of the cell suspension for single cell RNA sequencing. A) debris and nucleus were removed. B-C) doublets and triplets were removed, and singlets were gated. D) live cells were selected. E) CD45^+^ cells and CD45^-^ cells were divided and collected into two tubes respectively.**


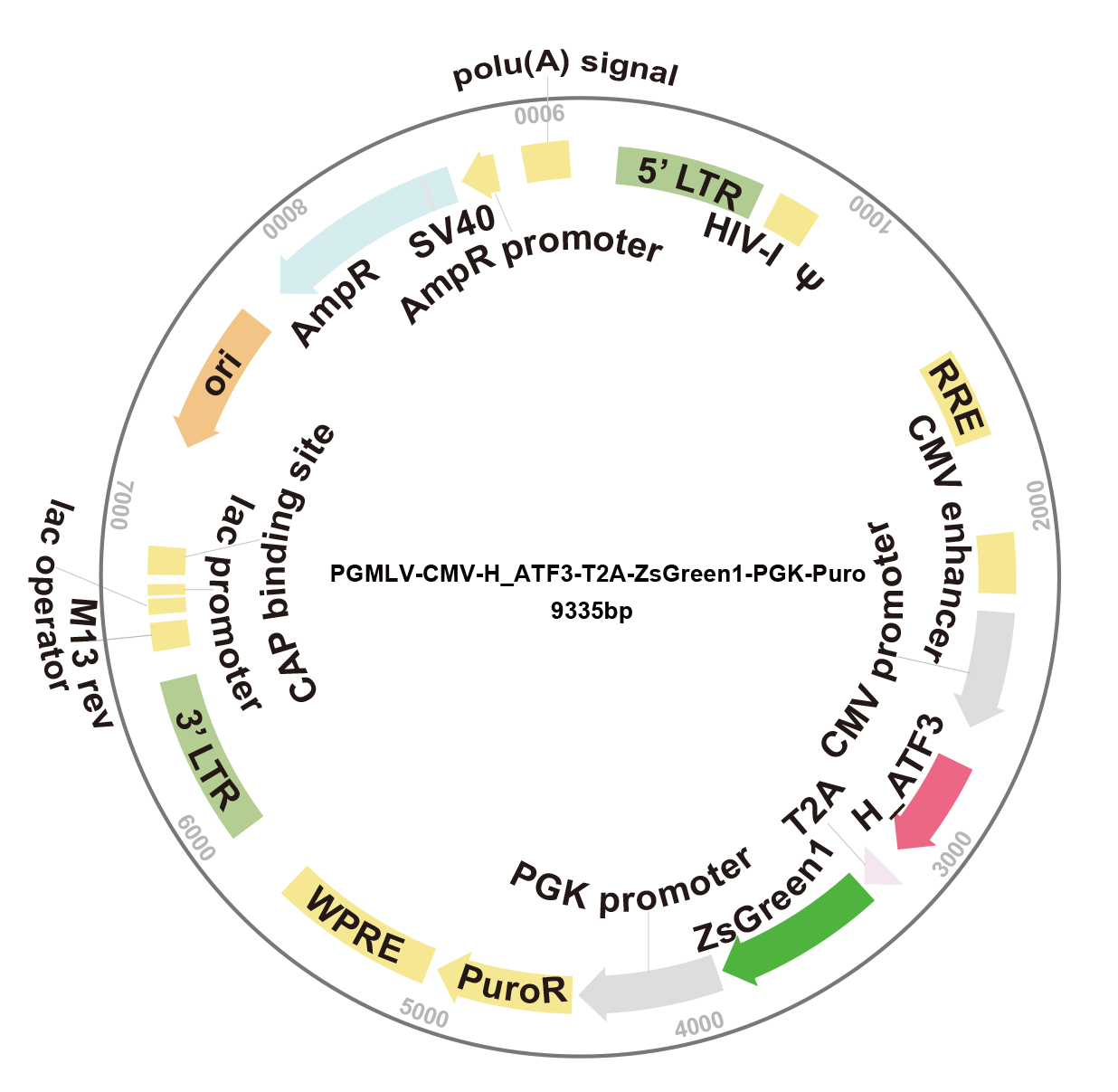


**Figure S2 The plasmid map of the lentiviral vector for overexpression of ATF3.**


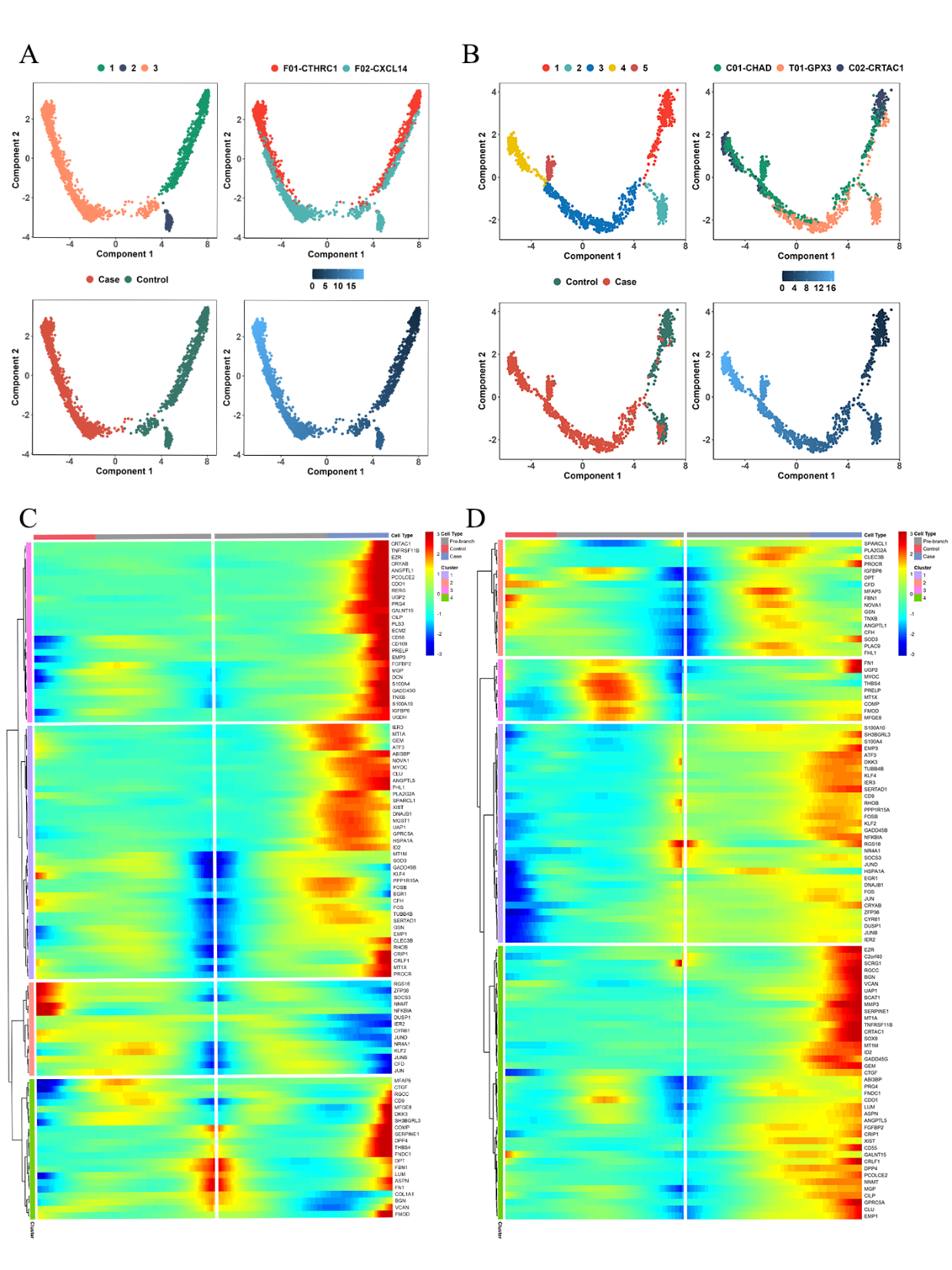


**Figure S3 Trajectory analysis of the stromal cells in the ligament tissue. A) result of trajectory analysis of the fibroblast-like cells showing the cells in different cell states, cell subclusters, sample types, and pseudo time. B) result of trajectory analysis of the chondrocyte-like cells and tenocyte-like cells showing the cells in different cell states, cell subclusters, sample types, and pseudo time. C) key genes in the trajectory of the fibroblast-like cells. D) key genes in the trajectory of the chondrocyte-like cells and tenocyte-like cells.**


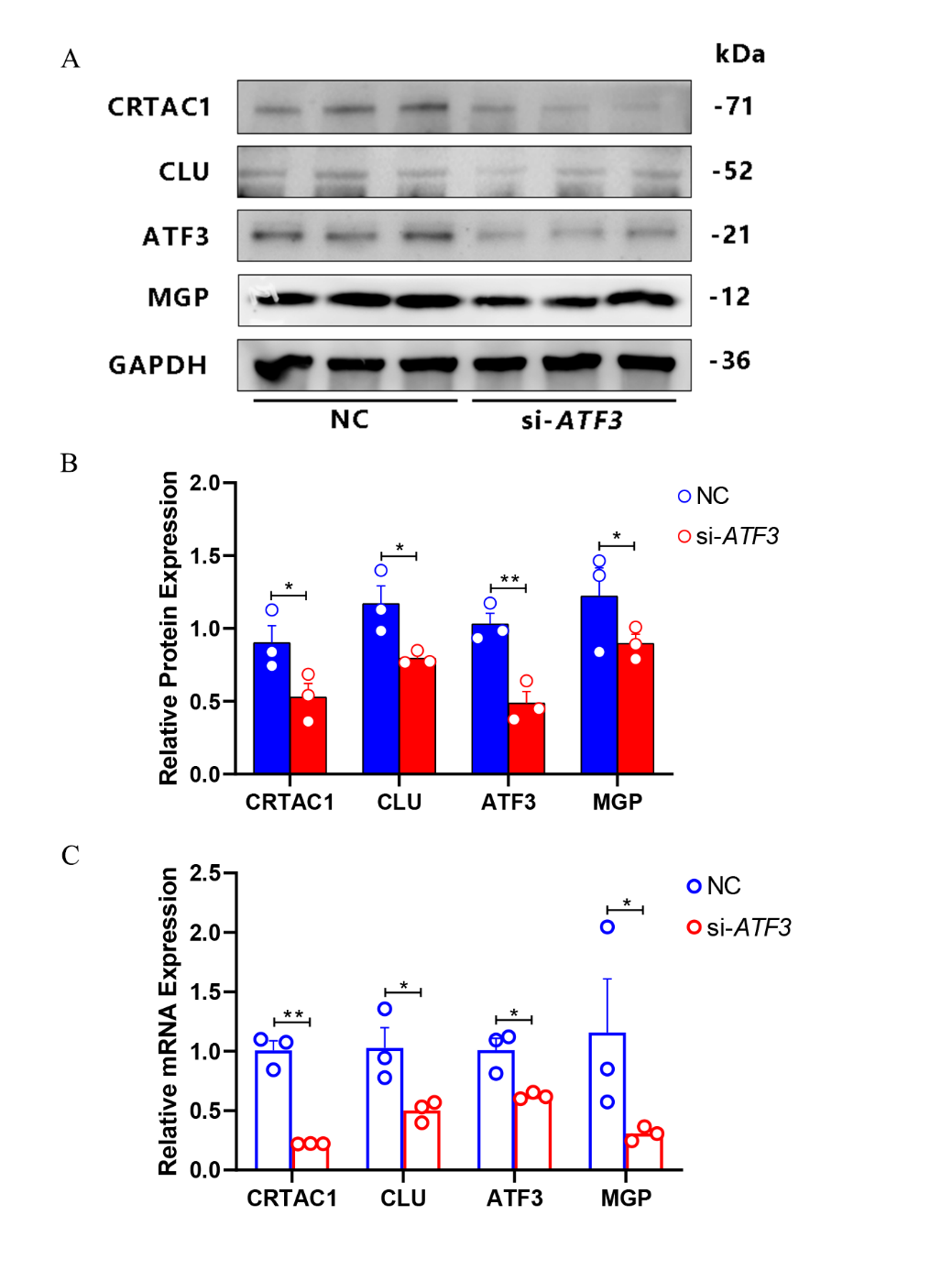


**Figure S4 The effects of si-ATF3 treatment in degenerative ligament cells. A) result of western blot analysis of cultured primary degenerative ligament cells with or without the treatment of si-ATF3. B) result of quantified intensity of western blotting bands. C) result of qPCR assay of cultured primary degenerative ligament cells with or without the treatment of si-ATF3.**


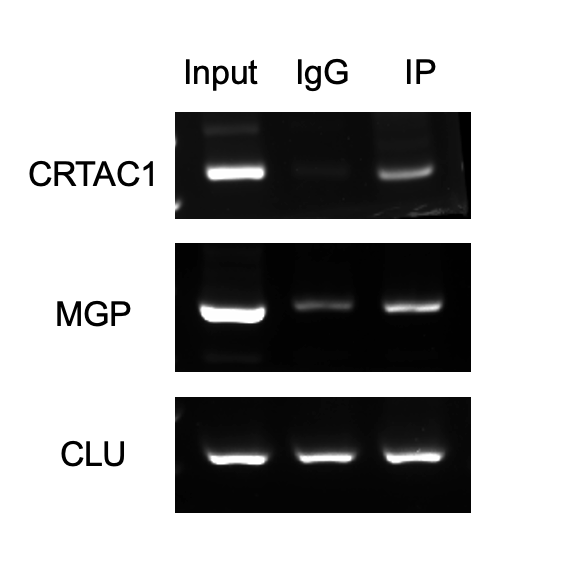


**Figure S5 ChIP-PCR analyses of CRTAC1, MGP, and CLU via reacting with immunoprecipitation of ATF3 antibodies.**


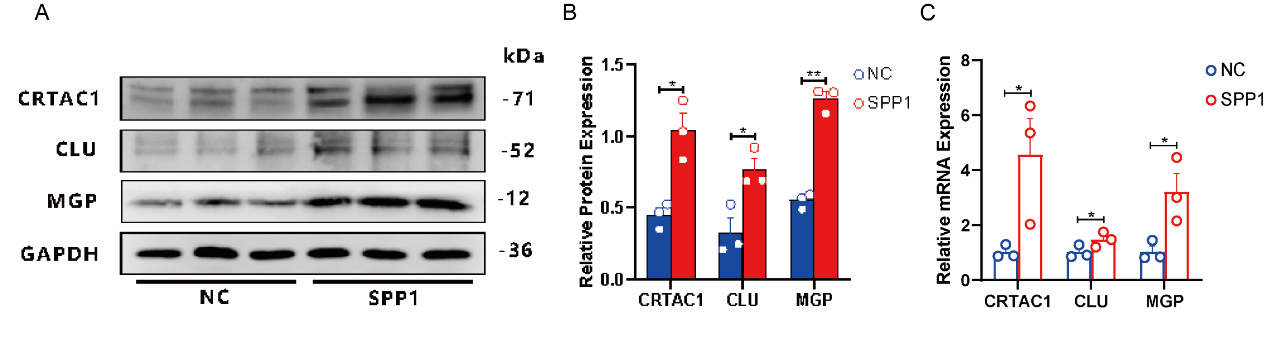


**Figure S6** **The effects of SPP1 in traumatic ligament cells. A) Western blot analysis of cultured primary non-degenerative ligament cells treated with or without recombinant human SPP1, quantified in B). C) QPCR expression analysis of chondrogenesis/osteogenesis markers in cells from (A).**


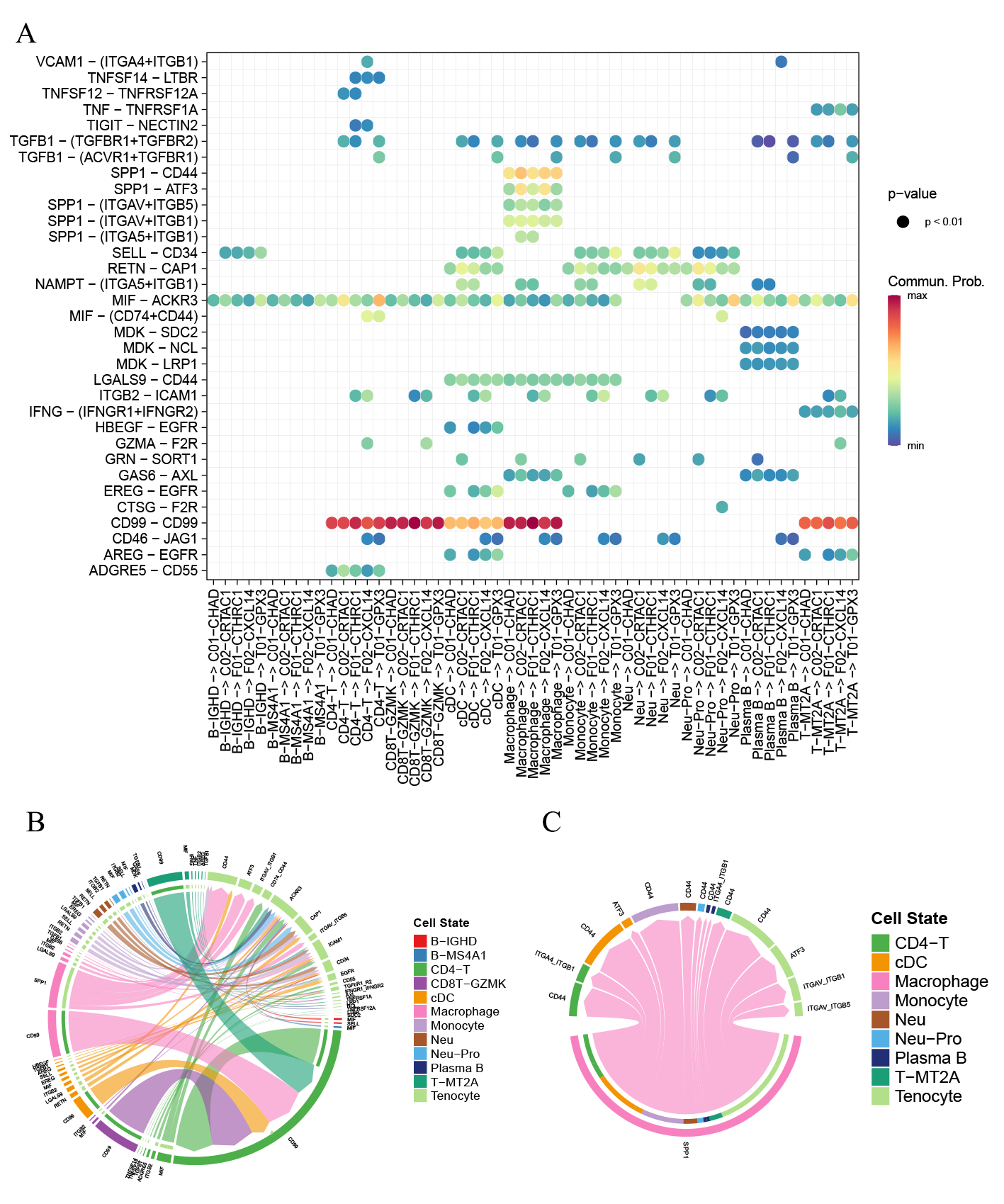


**Figure S7 Cell-cell communication analysis of ligand-receptor (L-R) interaction between immune cells and the stromal cells in ligament tissue. A) bubble plots showing selected ligand-receptor interactions between stromal and immune cell subpopulations in the ligament tissues. Circle size represents the corresponding p-value for one-sided permutation test. The color indicates the average expression level of interacting molecules in different subpopulations. Dark red indicates a relatively high expression level, while blue indicates a relatively low expression level. B-C) chord diagrams showing all the significant interactions (L-R pairs) between tenocyte as well as macrophage, respectively, and other immune cell subpopulations.**

**
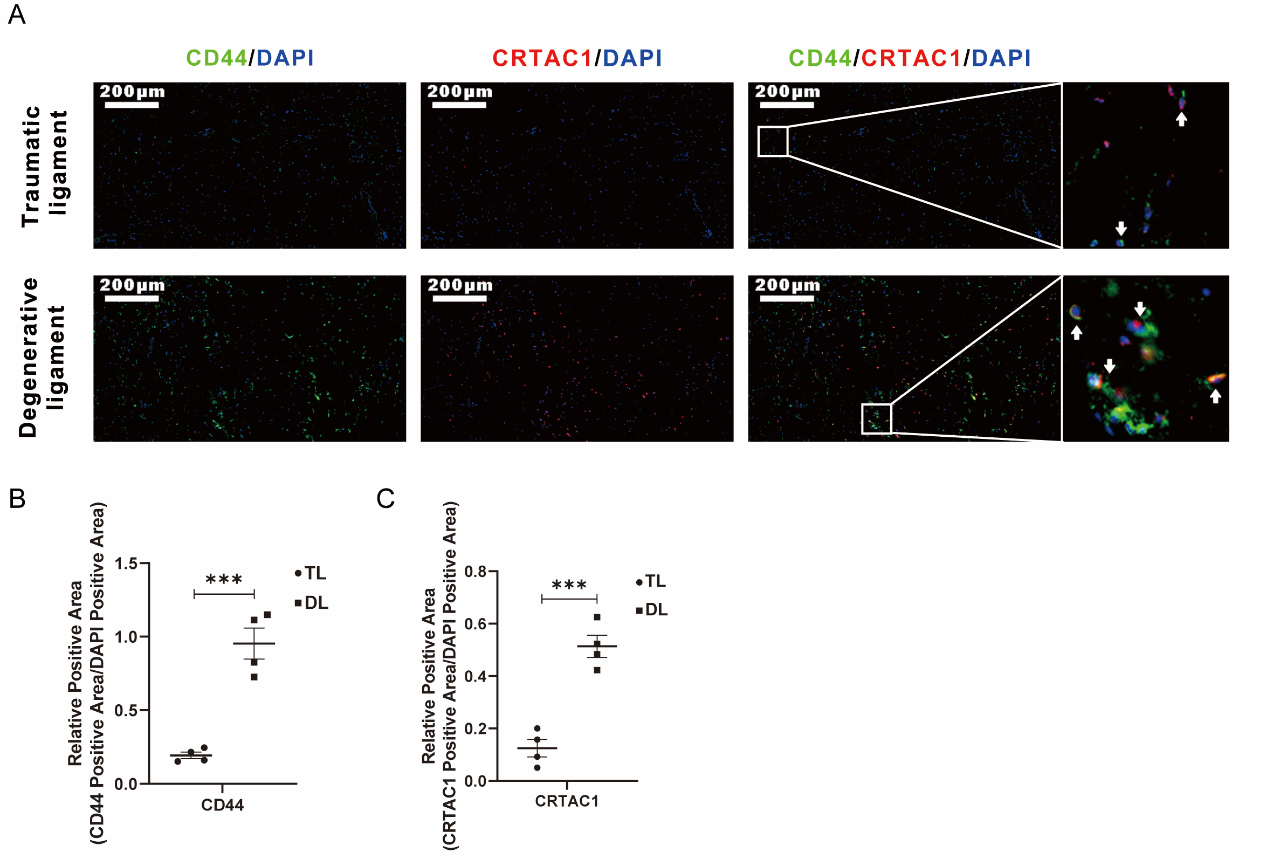
Figure S8 A)** **Immunofluorescent staining of CD44 and CRTAC1 in ligament tissues. (B, C) Quantification of the relative positive areas shown in A.**


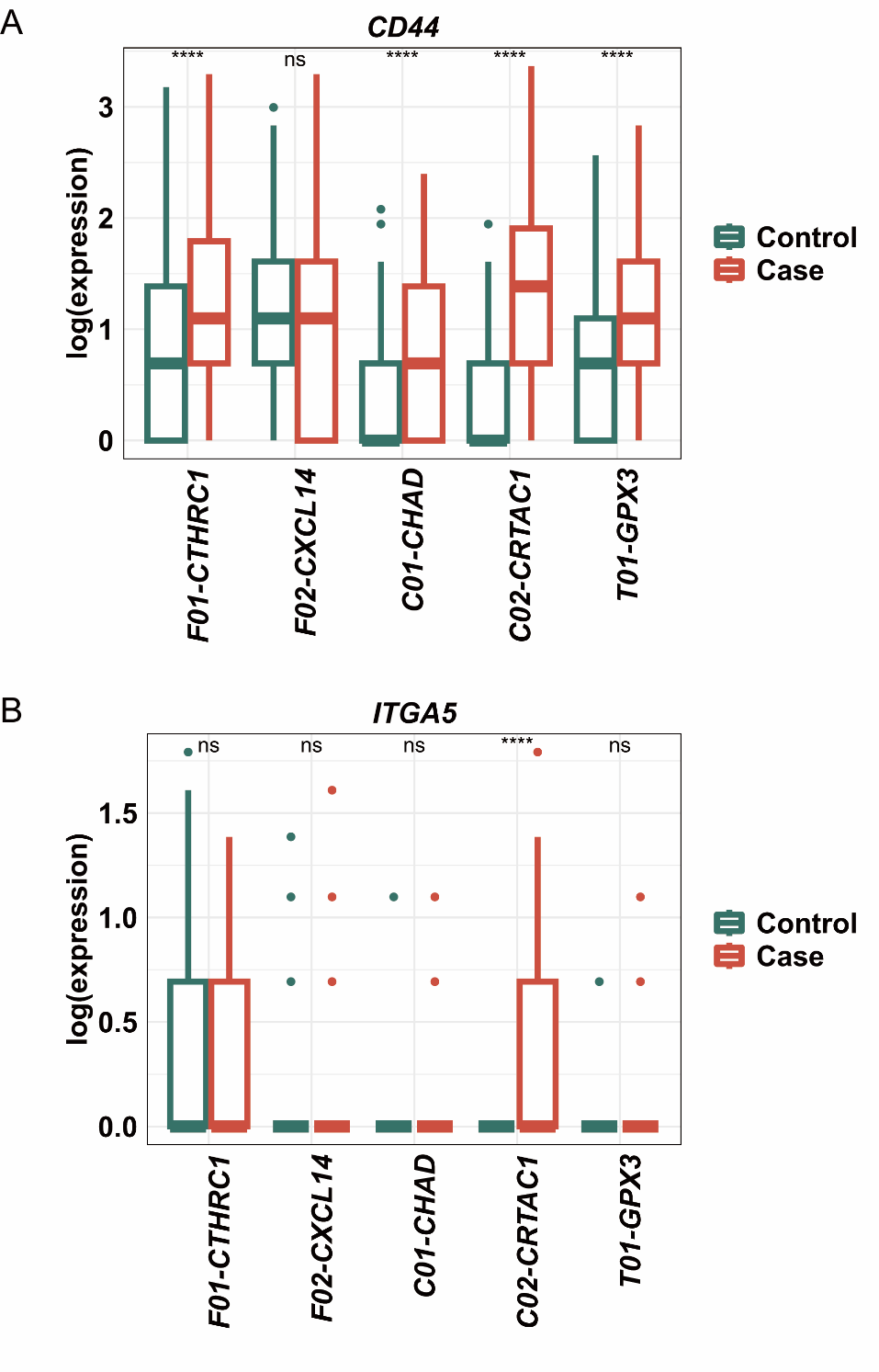


**Figure S9 The expression level of (A) CD44 (SPP1 receptors) and (B) ITGA5 in stromal ligament cell types.**


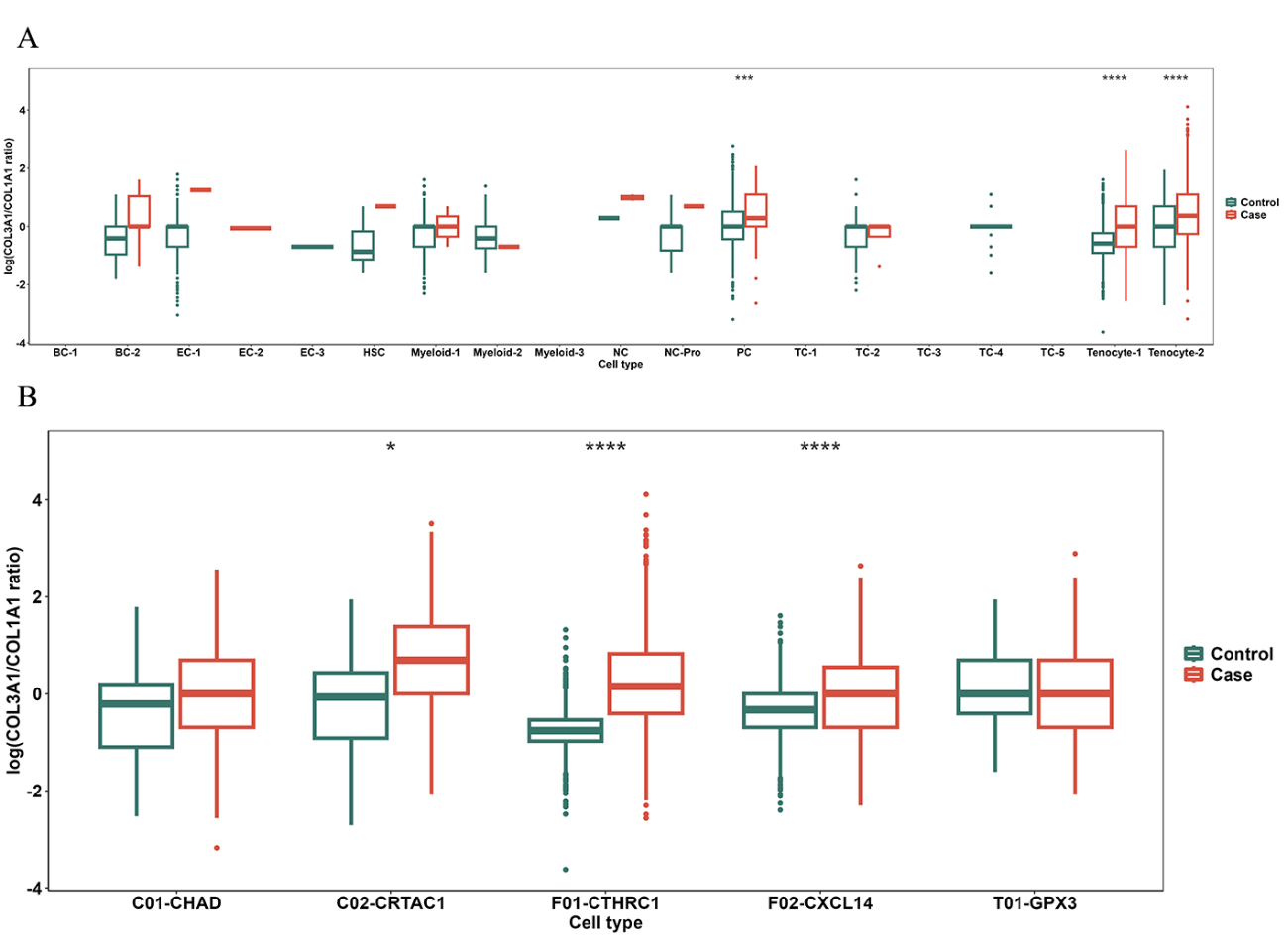


**Figure S10 The COL3/COL1 ratio of ligament tissues in different groups and subpopulations. A) the COL3/COL1 ratio in all cell types in the ligaments. B) the COL3/COL1 ratio in the stromal cell subpopulations in the ligaments.**


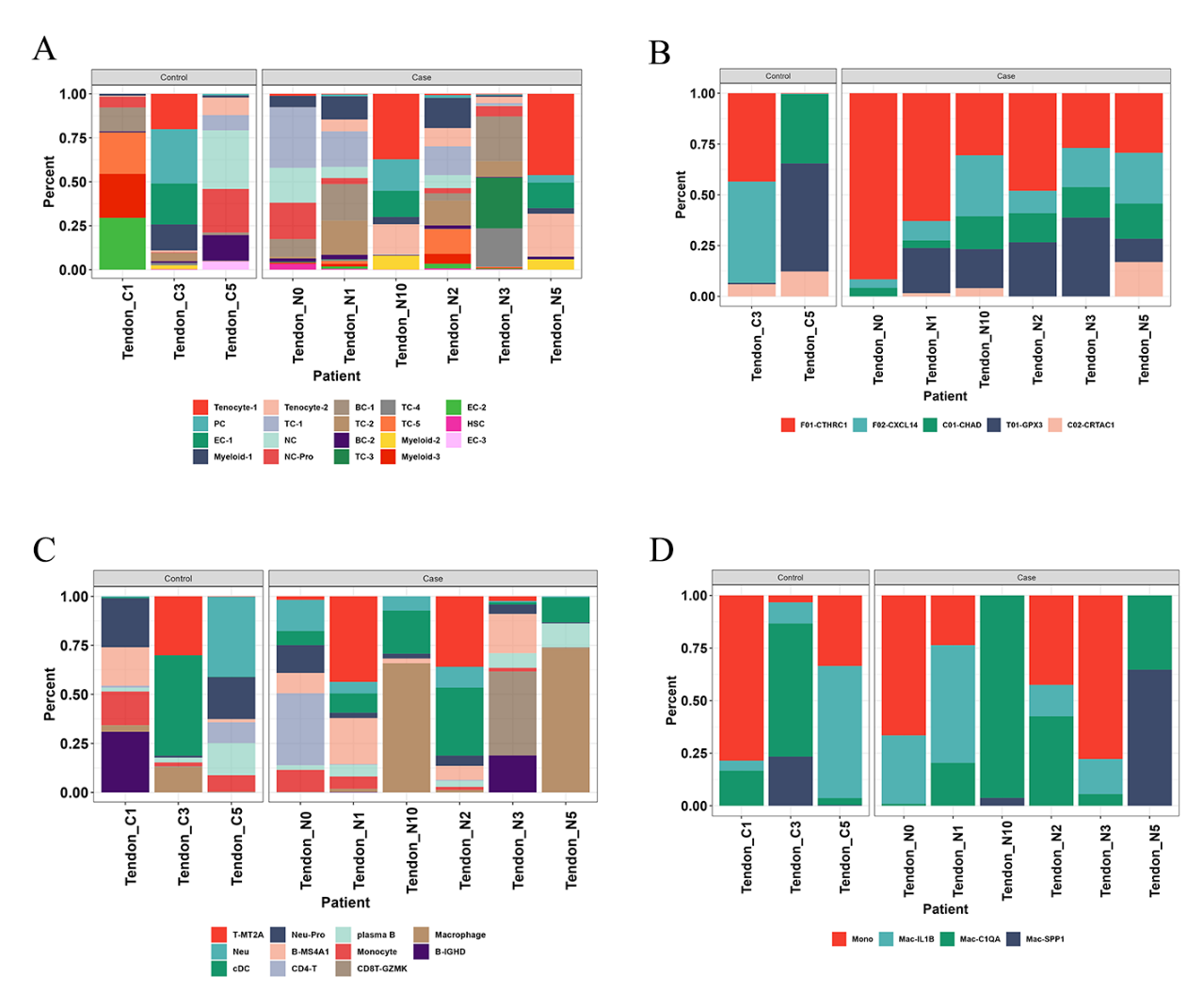


**Figure S11 The cell type distribution of each sample. A) all cell types in the cell atlas of the ligament tissue. B) the stromal cell subpopulations of the ligament tissue. C) the immune cell subpopulations of the ligament tissue. D) the monocyte and macrophage subpopulations of the ligament tissue.**


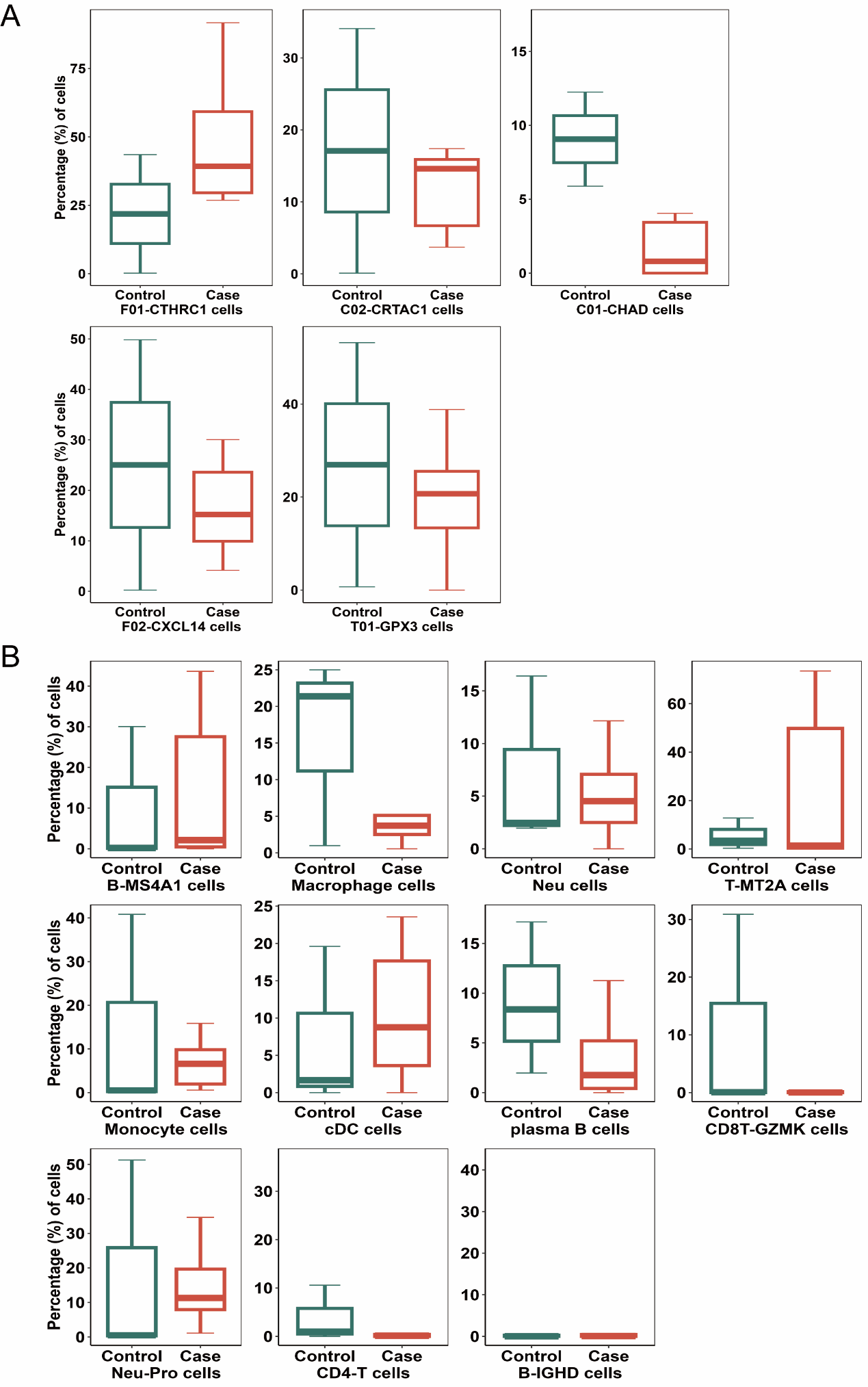


**Figure S12 The cell abundances for each cell type between six degenerative ligaments and three traumatic ligaments.**
