# Supplementary material for: Single‐cell RNA sequencing reveals the CRTAC1 + population actively contributes to the pathogenesis of spinal ligament degeneration by SPP1 + macrophage
Source: Aging Cell. 2024 Aug 19;23(12):e14320. doi: 10.1111/acel.14320 (PMC11634701; doi:10.1111/acel.14320)
Supplement: Supplementary file 2 — Table S1. [file ACEL-23-e14320-s004.docx]

**Table S1 Information of the clinical sample for single-cell RNA sequencing.**

| Sample ID | Gender | Age | Sample Type | Sample location | Cell numbers | Median genes |
| --- | --- | --- | --- | --- | --- | --- |
| Tendon_N0 | Female | 49 | Degenerative group | L5 | 3045 | 1367 |
| Tendon_N1 | Female | 51 | Degenerative group | L4 | 3025 | 992 |
| Tendon_N2 | Female | 74 | Degenerative group | L4-L5 | 2786 | 907.5 |
| Tendon_N3 | Female | 51 | Degenerative group | L3-L4 | 2768 | 767 |
| Tendon_N5 | Male | 57 | Degenerative group | L5 | 5125 | 2129 |
| Tendon_N10 | Male | 60 | Degenerative group | L4 | 940 | 2325.5 |
| Tendon_C1 | Female | 57 | Traumatic group | L2 | 760 | 1594.5 |
| Tendon_C3 | Male | 51 | Traumatic group | L1 | 9221 | 2044 |
| Tendon_C5 | Male | 56 | Traumatic group | L3 | 4344 | 943 |

**Table S2 The list of inflammatory-associated pathways signature genes.**

| Gene | Gene | Gene | Gene | Gene | Gene | Gene |
| --- | --- | --- | --- | --- | --- | --- |
| FABP4 | SELP | DPEP1 | STAT5B | SYT11 | RIPK2 | GSDMD |
| CD96 | ITGB6 | PDCD4 | ATRN | APOE | F8 | ADORA1 |
| SAA4 | IL1RN | CASP5 | CCR6 | APOA1 | EXT1 | CARD18 |
| HYAL2 | ADCYAP1 | DUSP10 | CCR5 | FEM1A | SOD1 | AXL |
| NCR3 | GGT3P | SCN9A | CYBB | CXCL1 | RPS19 | PPARG |
| IKBKB | SERPINF2 | SYK | CCR4 | KDM6B | MCPH1 | IL36RN |
| TREX1 | HCK | LY96 | CCR3 | AREL1 | TRAF3IP2 | XCL2 |
| CSRP3 | AHCY | VAMP7 | CCL11 | FANCD2 | NAIP | SMAD3 |
| HMGB1 | TNFSF4 | CXCL17 | ADAMTS12 | ADAM8 | SLC11A1 | STAT3 |
| MAP2K3 | SDC1 | SCYL3 | HAVCR2 | SIRPA | MFHAS1 | FOXP1 |
| PF4 | TCIRG1 | TLR1 | RAC1 | ADCY1 | CHST2 | PPARD |
| IL6ST | PXK | LTA | MMP3 | TLR10 | SNCA | TNFAIP8L2 |
| HDAC5 | CCL4 | TNF | IFNA2 | PLA2G2A | CCL4L1 | ITCH |
| C2CD4B | CD14 | CCR2 | PRKD1 | ISL1 | IL17B | TMSB4X |
| ADCY8 | IL2RA | GPR68 | GRN | MEFV | BMP6 | RELA |
| AIM2 | EPO | FPR1 | AGER | FFAR2 | HAMP | PSMA1 |
| CD44 | IL1B | IL15 | SCUBE1 | BIRC2 | TLR9 | TPST1 |
| FFAR3 | IL1A | XCL1 | BMPR1B | BIRC3 | APOL2 | IL18RAP |
| HYAL1 | IFNG | NLRP6 | LYZ | OGG1 | SELENOS | BDKRB2 |
| JAK2 | FOXP3 | IL1F10 | CD163 | NMI | APOL3 | SBNO2 |
| NOX4 | IFI35 | RARRES2 | SMPDL3B | CXCL3 | THBS1 | SNX4 |
| IL5 | ELANE | MGST2 | IL1RL2 | CXCL2 | BMP2 | IL6 |
| IL4 | UCN | PTGFR | LACC1 | EDNRB | LRFN5 | AZU1 |
| PBK | F2RL1 | PTGIR | SIGLEC1 | NLRC3 | TNFAIP6 | IL17RE |
| S100A8 | CCL21 | CCL13 | PLD3 | PTGDR | HK1 | NFATC4 |
| ITGB2 | ACKR2 | BCL6B | CCL20 | NFKB1 | MS4A2 | C4B |
| NLRC4 | METRNL | THEMIS2 | TAC4 | CMA1 | LYN | C4A |
| USP18 | AIF1 | FCGR2B | NAPEPLD | CREB3L3 | IL5RA | IL31RA |
| KLKB1 | TGFB1 | CD180 | ELF3 | GPS2 | VCAM1 | TBK1 |
| APP | LDLR | PARK7 | NLRX1 | SPATA2 | IGFBP4 | NFKBID |
| SUCNR1 | PRKCD | ZYX | IL18R1 | STAP1 | ICAM1 | GJA1 |
| NLRP1 | GHSR | PTN | CLOCK | PPARA | CELA1 | ITGA2 |
| CD6 | LIPA | CIITA | C1QTNF12 | HNRNPA0 | TNFSF18 | IL36A |
| LBP | CELF1 | ANO6 | MAS1 | IL6R | CYLD | RICTOR |
| TGM2 | AOAH | TRADD | PTGER1 | SCG2 | EPHA2 | CCR7 |
| CXCL6 | ADIPOQ | RB1 | JAM3 | CCL5 | TNIP3 | CCR1 |
| SETD6 | TTBK1 | F11R | CNR2 | CCL2 | AGTR1 | STK39 |
| NLRP9 | PSMB4 | CD47 | IL36G | IL18 | PSTPIP1 | REL |
| ABHD12 | NT5E | NOX1 | IL36B | LPCAT3 | TLR6 | NLRP12 |
| SCGB1A1 | CD200R1L | CD36 | IL37 | DUOXA2 | RPS6KA4 | GIT1 |
| BAP1 | TNFAIP3 | AP3B1 | GPRC5B | DUOXA1 | MACIR | ZBP1 |
| CCL17 | CCL3L1 | PTGS1 | TREM2 | NFX1 | LIAS | VNN1 |
| PROK2 | MYD88 | P2RX1 | PIK3AP1 | NFATC3 | IRGM | C3AR1 |
| PJA2 | FOS | CD200R1 | IL20 | FOXF1 | CXCR3 | NFKBIA |
| GATA3 | TIRAP | VAMP8 | CCL16 | AIMP1 | TMIGD3 | PNMA1 |
| SOCS3 | S100A9 | LOXL3 | HDAC4 | DDT | ADORA3 | CCL3 |
| KRT1 | POLB | TOLLIP | TLR3 | PRDX5 | CASP4 | CXCL8 |
| AGTR2 | SELE | FFAR4 | CCL25 | GPR31 | GAL | CD40 |
| LILRA5 | CCL22 | ETS1 | HGF | FPR2 | TUSC2 | C2CD4A |
| CYBA | CMKLR1 | KARS1 | FOLR2 | CXCR2 | FUT7 | DEFB114 |
| KRT16 | KLRG1 | IDO1 | IL22 | ACOD1 | PTPN2 | ZC3H12A |
| PLP1 | LTB4R | TNIP1 | MAPK13 | NDFIP1 | CXCL13 | IRF3 |
| STAB1 | CNR1 | LGALS9 | NR1H4 | CCL18 | CHST1 | ITIH4 |
| GPR17 | UFL1 | MMP9 | MAPK7 | CCL23 | TYRO3 | H2BC1 |
| BDKRB1 | CCN3 | PTPRC | PTGER4 | PTGES | F12 | TAC1 |
| IRF5 | PIK3CG | CHIA | NR1H3 | ENPP3 | HDAC9 | ZNF580 |
| C1QTNF3 | KNG1 | TICAM1 | NLRP2 | NOS2 | HPR | DAGLB |
| CLEC7A | TIMP1 | IL17D | XCR1 | CD5L | HP | CHST4 |
| BCL6 | C5 | MVK | GPR4 | AHSG | F2 | GHRL |
| PLA2G2D | C3 | CAMK1D | PGLYRP2 | GPR32 | IFI16 | CYSLTR1 |
| IGF1 | AGT | DNASE1L3 | IL17F | RBPJ | HIF1A | CCL14 |
| MRGPRX1 | SERPINA3 | TRIM55 | WDR83 | HYAL3 | PTGIS | ECM1 |
| SIGLEC10 | SERPINA1 | IL17RA | DHX9 | CCL26 | EGFR | CALCRL |
| PSMA6 | SERPINC1 | CCRL2 | TSPAN2 | PLAA | GPX1 | KLF4 |
| B4GALT1 | OSMR | ABCC2 | GSTP1 | DAB2IP | SHARPIN | CXCL5 |
| MSMP | PRKCZ | PDE2A | NLRP3 | C5AR2 | ABCF1 | CCL1 |
| ASH1L | MGLL | ODAM | XIAP | ACKR1 | RPS6KA5 | PARP4 |
| IL9 | AKT1 | HFE | HSPG2 | CAMK4 | CD28 | CEBPB |
| FNDC4 | TRIL | GPER1 | GGT1 | IL17A | KIT | UGT1A1 |
| TEK | CST7 | S1PR3 | TNFRSF1A | IL23R | PF4V1 | IL10 |
| IL22RA2 | PTGER2 | HMGB2 | BRD4 | CSF1R | BTK | MAPKAPK2 |
| TSLP | PTGER3 | SHPK | MIF | PRCP | SAA2 | SEMA7A |
| HLA-E | SMAD1 | CCL24 | CXCL10 | ESR1 | SAA1 | ASS1 |
| F2R | MBL2 | SNAP23 | PPBP | SIGIRR | UMOD | SCYL1 |
| F3 | NPY5R | WNT5A | CD40LG | LAT | MMP8 | AOC3 |
| OSM | PYDC5 | CD200 | ORM1 | LRRC19 | SERPINE1 | TNFRSF1B |
| IL2 | CCL19 | PYDC2 | FN1 | CCN4 | LY75 | MEP1B |
| LXN | BLNK | NOD2 | APCS | DROSHA | CTNNBIP1 | MYLK3 |
| PTAFR | IKBKG | ALOX15 | CRP | TFR2 | NLRP4 | IL17C |
| TACR1 | MDK | SGMS1 | GPR33 | PLA2G4C | PRDX2 | NR1D1 |
| RORA | CASP12 | PLGRKT | IL4R | RELB | ITGAL | PLD4 |
| TNFSF11 | CXCR6 | CYP4F11 | CRHBP | CASP1 | ACVR1 | IL27 |
| CXCR4 | SERPINF1 | NLRP10 | PLSCR1 | IL12B | NFE2L2 | SOCS5 |
| OPRM1 | CCL7 | IL21 | VPS35 | MMP26 | PRKCQ | GPSM3 |
| HRH1 | CCL8 | CX3CL1 | PLA2G7 | NUPR1 | GPR32P1 | NFAM1 |
| PTGS2 | BST1 | HLA-DRB1 | NPFF | IL34 | NOTCH2 | DAGLA |
| GBA | INS | NOTCH1 | CHUK | CERS6 | TNIP2 | PYCARD |
| PROC | TNFRSF4 | PTX3 | ORM2 | CYP26B1 | IL33 | CDO1 |
| IL10RB | LRRK2 | CXCL11 | PLA2G3 | IL1RAP | SPP1 | TLR5 |
| ANXA1 | TLR8 | ALOX5 | IL13 | ADORA2A | NR1D2 | TLR2 |
| ADM | LPL | ACE2 | ATM | IL23A | OTULIN | REG3A |
| GBP5 | CRH | GGT5 | TLR7 | CEBPA | ADGRE2 | NDST1 |
| NFKBIZ | CTSC | GGT2 | CD68 | LTB4R2 | RHBDD3 | WFDC1 |
| CXCL9 | ADGRE5 | DNASE1 | SPHK1 | MMP25 | TNFRSF11A | IL16 |
| TAFA3 | CDH5 | CHI3L1 | UNC13D | PLA2G4B | ITGB1 | TLR4 |
| AFAP1L2 | ZP3 | OLR1 | FPR3 | IL17RC | IRAK2 | IL1R1 |
| ABCC1 | TBXA2R | RIPK1 | CSF1 | EIF2AK1 | SLAMF8 | S100A12 |
| TICAM2 | C5AR1 | NRROS | HMOX1 | TREM1 | NOD1 | CDK19 |
| LY86 | CALCA | REG3G | FANCA | HRH4 | IL25 | CD81 |
| AKNA | IL20RB | PIK3CD | IL1RL1 | ADA | CCL15 | PLA2G2E |
| STING1 |  |  |  |  |  |  |

**Table S3 The primer sequences used in this study.**

|  | Forward (5′-3′) | Reverse (5′-3′) |
| --- | --- | --- |
| GAPDH | ATAAATTGAGCCCGCAGCC | CCCAATACGACCAAATCCGTTG |
| ATF3 | CGGAGCCTGGAGCAAAATGA | GGATGGCAAACCTCAGCTCT |
| CLU | GGAGATCTTGTCTGTGGACTGTT | CTGGTCAACCTCTCAGCGAC |
| CRTAC1 | GCTAAGGTCGTGCTCTACACCA | ACCTCCACACTGCTGGCTTCAT |
| MGP | TTTGTGTTATGAATCACATGAAAGC | AGCGTTCTCGGATCCTCTCT |
| PCOLCE2 | AGGAGTCACTTGTGTGTGGC | TCAGACACAATTGGCGCAGG |

**Table S4 GSVA enrichment analysis of fibroblast-like cells of the ligament tissue.**

| Description | NES | pvalue | qvalues | Gene |
| --- | --- | --- | --- | --- |
| extracellular matrix organization | -2.41477 | 3.37E-03 | 1.89E-02 | ACAN/COLGALT1/CAPN1/QSOX1/LAMB2/KAZALD1/PTX3/COL4A1/AGT/CTSL/EGFL6/MATN4/BSG/GPM6B/FBLN2/MMP16/NID2/GREM1/GAS6/MMP19/ADAMTS4/ENG/ADAMTS14/COL10A1/TNFRSF1A/NID1/TGFB1/AEBP1/A2M/SH3PXD2B/COL15A1/VCAM1/COL7A1/ICAM1/LAMC3/FAP/BGN/LAMB1/THBS1/COL4A2/FBLN1/PLOD1/TGFBI/IL6/TIMP1/FBLN5/COL12A1/CRISPLD2/SH3PXD2A/B4GALT1/COL6A3/LOXL1/FSCN1/MFAP4/P3H4/LOXL2/HSPG2/MMP2/ADAMTS2/HTRA1/TNC/COL5A3/EFEMP2/PRDX4/BMP1/COL18A1/CTSK/MFAP2/EMILIN1/COL11A1/COL6A2/CREB3L1/MMP14/CCDC80/SERPINH1/COL16A1/COL6A1/SFRP2/LOX/ADAM12/COL5A2/MMP23B/MMP11/COL5A1/ELN/COL3A1/COL1A2/POSTN/SPARC/COL1A1 |
| extracellular structure organization | -2.3962 | 3.38E-03 | 1.89E-02 | ACAN/COLGALT1/CAPN1/QSOX1/LAMB2/KAZALD1/PTX3/COL4A1/AGT/CTSL/EGFL6/MATN4/BSG/GPM6B/FBLN2/MMP16/NID2/GREM1/GAS6/MMP19/ADAMTS4/ENG/ADAMTS14/COL10A1/TNFRSF1A/NID1/TGFB1/AEBP1/A2M/SH3PXD2B/COL15A1/VCAM1/COL7A1/ICAM1/LAMC3/FAP/BGN/LAMB1/THBS1/COL4A2/FBLN1/PLOD1/TGFBI/IL6/TIMP1/FBLN5/COL12A1/CRISPLD2/SH3PXD2A/B4GALT1/COL6A3/LOXL1/FSCN1/MFAP4/P3H4/LOXL2/HSPG2/MMP2/ADAMTS2/SDC1/HTRA1/TNC/P4HB/COL5A3/EFEMP2/PRDX4/BMP1/COL18A1/CTSK/MFAP2/EMILIN1/COL11A1/COL6A2/CREB3L1/MMP14/CCDC80/SERPINH1/COL16A1/COL6A1/SFRP2/LOX/ADAM12/COL5A2/MMP23B/MMP11/COL5A1/ELN/COL3A1/COL1A2/APOE/POSTN/SPARC/COL1A1 |
| ossification | -1.82384 | 3.45E-03 | 1.90E-02 | WNT5A/GPM6B/MMP16/RUNX2/TNN/DCHS1/CEBPB/GPC3/GREM1/OSR2/FZD1/SHOX2/TGFB1/TGFB3/ECM1/FSTL3/SH3PXD2B/ROR2/CHRD/LGR4/JUNB/TPM4/IL6/TWIST1/OMD/JUND/MRC2/IGFBP3/MMP2/PDLIM7/TMEM119/ISG15/IGFBP5/TNC/TWIST2/BMP1/GJA1/CDH11/CTSK/COL11A1/CREB3L1/MMP14/COL6A1/SFRP2/LOX/COL5A2/CLEC11A/PTN/MDK/CTHRC1/COL1A2/SPARC/COL1A1 |
| cellular respiration | -1.76813 | 2.95E-03 | 1.89E-02 | NDUFA12/NDUFS7/NDUFA9/SLC25A22/NDUFV2/UQCRC1/BID/CYCS/MDH1/NDUFC2/NDUFA8/SDHAF2/PDHB/TRAP1/DNAJC15/NDUFB10/UQCRC2/SHMT2/NFATC4/COX4I1/SURF1/COX5B/NDUFB5/BLOC1S1/VCP/MDH2/NDUFB8/ETFA/COX8A/ETFB/NDUFS3/NDUFB7/UQCRH/CISD1/NDUFS2/NDUFV1/CYC1/UQCRFS1/COX7A2L/NDUFA3/COX5A/NDUFA13/COX7B/NDUFAB1/COX6C/PRELID1/NDUFS6/NDUFA6/UQCRQ/PARK7/NDUFB11/ATP5F1D/NDUFS8/NDUFB2/UQCR11/UQCR10/IDH2/NDUFA4 |
| leukocyte migration | -1.73298 | 3.34E-03 | 1.89E-02 | BDKRB1/FN1/SLC7A6/SLC7A8/MIF/ZNF580/ADA/CALR/CYP7B1/AKT1/SLC16A3/C1QBP/NA/WDR1/VEGFA/CCL21/SDC3/WNT5A/GPC1/BSG/CXCL1/MYH9/GREM1/GAS6/SHC1/TGFB1/ECM1/ROR2/VCAM1/ICAM1/LGMN/VEGFB/NBL1/THBS1/CH25H/CXCL8/IL6/APOD/CCL19/B4GALT1/SDC1/CXCL12/EMILIN1/CXCL3/MMP14/DUSP1/NA/CXCL2/NA/PTN/MDK/THY1/RARRES2/COL1A2/CXCL14/COL1A1 |
| blood vessel development | -1.7204 | 4.10E-03 | 1.90E-02 | WNT2/ATP5F1B/COL4A1/BAX/TNFAIP2/AGT/SNX17/CIB1/HSPB6/ZFP36L1/WNT5A/UNC5B/AHR/PLXND1/PLXDC1/ATP5IF1/MYH9/CTSH/GPC3/GREM1/MMP19/ENG/SHC1/RAMP1/TNFRSF12A/ACVRL1/EPHB3/TGFB1/JUN/ECM1/SEMA5A/C3/COL15A1/FAP/VEGFB/SERPINF1/NR4A1/ADGRA2/HPGD/THBS1/COL4A2/EDNRA/CXCL8/TGFBI/JUNB/IL6/SPHK1/APOD/TWIST1/B4GALT1/LOXL1/NOTCH3/MYDGF/LOXL2/HSPG2/TNFAIP3/TYMP/GRN/MMP2/PTK7/GJA1/COL18A1/EMILIN1/CREB3L1/MMP14/SFRP2/LOX/ACTA2/ADAM12/THBS2/PTN/MDK/THY1/COL5A1/COL3A1/COL1A2/APOE/SPARC/COL1A1 |
| cell-substrate adhesion | -1.69631 | 3.36E-03 | 1.89E-02 | NINJ1/VEGFA/NRP1/LIMS1/RAB1A/CTTN/SORBS3/PARVB/CCL21/RAC3/LAMB2/TRIP6/CIB1/EGFL6/GPM6B/FBLN2/GBP1/ACTN4/TNN/TRIOBP/NID2/ZYX/GAS6/TNFRSF12A/ACVRL1/ACTN1/EPHB3/NID1/VCAM1/ADAMTS12/LAMC3/LAMB1/THBS1/FBLN1/ITGBL1/FLNA/APOD/FBLN5/CD63/HSPG2/PLAU/COL5A3/LGALS1/EMID1/EMILIN1/MMP14/CCDC80/COL16A1/PTN/MDK/THY1/COL3A1/POSTN/COL1A1 |
| negative regulation of cell migration | -1.69434 | 3.17E-03 | 1.89E-02 | ADAMTS9/APEX1/NR2F2/ILK/MIIP/TRIB1/NOTCH1/MIF/ADA/IFITM1/CALR/JAG1/AKT1/CCL21/STRAP/ARHGDIB/BST2/NRG1/EVL/HYAL2/TNN/GREM1/PRKG1/ENG/ACVRL1/TGFB1/GSTP1/CHRD/SEMA3A/PODN/NBL1/SERPINF1/THBS1/IDH2/APOD/TIMP1/TPM1/IGFBP3/CXCL12/IGFBP5/EMILIN1/DUSP1/SFRP2/PTN/THY1/COL3A1/APOE |
| cardiovascular system development | -1.68931 | 4.05E-03 | 1.90E-02 | WNT2/ATP5F1B/COL4A1/BAX/TNFAIP2/AGT/SNX17/LIF/CIB1/HSPB6/ZFP36L1/WNT5A/UNC5B/NDUFS6/AHR/PLXND1/PLXDC1/ATP5IF1/MYH9/CTSH/GPC3/GREM1/MMP19/ENG/SHC1/RAMP1/TNFRSF12A/ACVRL1/EPHB3/TGFB1/JUN/ECM1/SEMA5A/C3/COL15A1/FAP/VEGFB/NXN/SERPINF1/NR4A1/ADGRA2/HPGD/THBS1/COL4A2/EDNRA/CXCL8/TGFBI/JUNB/IL6/SPHK1/APOD/TWIST1/B4GALT1/LOXL1/NOTCH3/MYDGF/LOXL2/HSPG2/TNFAIP3/TYMP/GRN/MMP2/PTK7/GJA1/COL18A1/EMILIN1/CREB3L1/MMP14/SFRP2/LOX/ACTA2/ADAM12/THBS2/PTN/MDK/THY1/COL5A1/COL3A1/COL1A2/APOE/SPARC/COL1A1 |
| vasculature development | -1.68587 | 3.97E-03 | 1.90E-02 | WNT2/ATP5F1B/COL4A1/BAX/TNFAIP2/AGT/SNX17/LIF/CIB1/HSPB6/ZFP36L1/WNT5A/UNC5B/AHR/PLXND1/PLXDC1/ATP5IF1/MYH9/CTSH/GPC3/GREM1/MMP19/ENG/SHC1/RAMP1/TNFRSF12A/ACVRL1/EPHB3/TGFB1/JUN/ECM1/SEMA5A/C3/COL15A1/FAP/VEGFB/SERPINF1/NR4A1/ADGRA2/HPGD/THBS1/COL4A2/EDNRA/CXCL8/TGFBI/JUNB/IL6/SPHK1/APOD/TWIST1/B4GALT1/LOXL1/NOTCH3/MYDGF/LOXL2/HSPG2/TNFAIP3/TYMP/GRN/MMP2/PTK7/GJA1/COL18A1/EMILIN1/CREB3L1/MMP14/SFRP2/LOX/ACTA2/ADAM12/THBS2/PTN/MDK/THY1/COL5A1/COL3A1/COL1A2/APOE/SPARC/COL1A1 |
| negative regulation of cell motility | -1.68284 | 3.16E-03 | 1.89E-02 | ADAMTS9/APEX1/NR2F2/ILK/MIIP/TRIB1/NOTCH1/MIF/ADA/IFITM1/CALR/JAG1/AKT1/CCL21/STRAP/ARHGDIB/BST2/NRG1/EVL/HYAL2/TNN/GREM1/PRKG1/ENG/ACVRL1/TGFB1/GSTP1/CHRD/SEMA3A/PODN/NBL1/SERPINF1/THBS1/FBLN1/IDH2/APOD/TIMP1/TPM1/IGFBP3/CXCL12/IGFBP5/EMILIN1/DUSP1/SFRP2/PTN/THY1/COL3A1/APOE |
| bone development | -1.67714 | 2.99E-03 | 1.89E-02 | MATN4/MMP16/RUNX2/DCHS1/GREM1/ENG/OSR2/SHOX2/TGFB1/TGFB3/SH3PXD2B/SFRP4/COL7A1/COCH/LRRC17/VKORC1/TWIST1/COL12A1/COL6A3/P3H1/TMEM119/PPIB/GJA1/COL6A2/MMP14/SERPINH1/COL6A1/SFRP2/LOX/SPARC/COL1A1 |
| cellular response to acid chemical | -1.67469 | 3.06E-03 | 1.89E-02 | LDLR/AKR1C3/LAMTOR4/AKT1/CYP26B1/VEGFA/PTGFR/WNT2/COL4A1/NDUFA13/WNT5A/CDK4/CEBPB/TWF2/LAMTOR1/SERPINF1/CYBA/NME1/MMP2/TNC/PTK7/COL16A1/COL6A1/COL5A2/COL3A1/COL1A2/COL1A1 |
| skin development | -1.66496 | 3.00E-03 | 1.89E-02 | LDB2/TNFRSF19/FST/NOTCH1/ELOVL1/AKR1C3/JAG1/SMO/CYP26B1/TRPC4AP/RUNX1/CAPN1/CTSL/PALLD/ZFP36L1/WNT5A/KRT10/FOSL2/LGR4/ZFP36/APCDD1/ADAMTS2/IGFBP5/COL5A3/CTSK/COL5A2/COL5A1/COL3A1/COL1A2/COL1A1 |
| negative regulation of locomotion | -1.66469 | 3.28E-03 | 1.89E-02 | ADAMTS9/ELANE/APEX1/NR2F2/ILK/MIIP/TRIB1/NOTCH1/MIF/ADA/IFITM1/CALR/JAG1/AKT1/PML/NRP1/CCL21/STRAP/ARHGDIB/BST2/NRG1/WNT5A/EVL/HYAL2/TNN/GREM1/PRKG1/ENG/ACVRL1/TGFB1/GSTP1/SEMA5A/CHRD/SEMA3A/PODN/NBL1/SERPINF1/THBS1/FBLN1/IDH2/APOD/TIMP1/TPM1/IGFBP3/CXCL12/IGFBP5/EMILIN1/DUSP1/SFRP2/PTN/THY1/COL3A1/APOE |
| response to heat | 2.029614 | 1.51E-03 | 1.89E-02 | CRYAB/HSPA1B/DNAJB1/HSPA1A/HSP90AA1/HSP90AB1/HSPA6/HSPH1/DNAJA1/SCARA5/SOD1/PSIP1/CDKN1A/HSPA8/DNAJB4/IGF1/BAG3/TPR/HSPB8/HSPA5/CHORDC1/PTGES3/YWHAE/DHX36/HSPA2/SIRT1/SLU7/MYOF/BAG5/EP300/MAPK1/ATM/DNAJA2/HSPA9/NUP153/BAG2/RBBP7/FKBP4/DNAJB6 |
| response to temperature stimulus | 1.932195 | 1.46E-03 | 1.89E-02 | CRYAB/HSPA1B/DNAJB1/HSPA1A/HSP90AA1/HSP90AB1/HSPA6/HSPH1/DNAJA1/HSPD1/SCARA5/SOD1/PSIP1/CDKN1A/HSPA8/DNAJB4/IGF1/BAG3/TPR/HSPB8/FOXO1/ATP2B1/HSPA5/CHORDC1/PTGES3/NFE2L1/YWHAE/DHX36/YBX3/HSPA2/DNAJC3/SIRT1/FOS/SLU7/THRA/MYOF/BAG5/EP300/MAPK1/ATM/DNAJA2/HSPA9/NUP153/BAG2/RBBP7/FKBP4/ADM/DNAJB6 |
| protein stabilization | 1.890857 | 1.48E-03 | 1.89E-02 | CLU/CRYAB/HSPA1B/HSPA1A/HSP90AA1/PIK3R1/HSP90AB1/ANK2/HSPD1/CDKN1A/PER3/IGF1/BAG3/SUGT1/PTGES3/UBE2B/RTN4/TCP1/CAMLG/LAMP2/ATF7IP/LAMP1/BAG5/USP9X/MORC3/HIP1/ZNF207/EP300/PDCD10/USP7/CCT4/FBXW7/CCT2/CREB1/USP33/BAG2/SMAD3/SAV1 |
| regulation of protein stability | 1.856776 | 1.44E-03 | 1.89E-02 | CLU/CRYAB/HSPA1B/GSN/HSPA1A/HSP90AA1/PIK3R1/HSP90AB1/ANK2/HSPD1/CDKN1A/HSPA8/PER3/IGF1/BAG3/CDKN2AIP/SUGT1/PTGES3/PRKDC/UBE2B/FBXL3/RTN4/VPS35/TCP1/CAMLG/MYLIP/SIRT1/LAMP2/ATF7IP/USP8/USP34/ASPH/CD81/LAMP1/BAG5/CUL3/USP9X/MORC3/HIP1/BCL2/ID1/ZNF207/USP47/EP300/PDCD10/USP7/MAPK1/CCT4/BMP2/FBXW7/CCT2/FBXW11/CREB1/USP33/CDC73/MDM2/BAG2/SMAD3/SAV1/CASP3 |
| response to topologically incorrect protein | 1.854969 | 1.45E-03 | 1.89E-02 | CLU/HSPA1B/DNAJB1/HSPA1A/THBS4/HSP90AA1/PIK3R1/HSP90AB1/HSPA6/COMP/HSPH1/DNAJA1/HSPB1/HSPD1/HSPA8/CREBRF/HSPE1/DNAJB4/HSP90B1/BAG3/OPTN/HSPB8/TLN1/NFE2L2/CANX/HSPA5 |
| RNA splicing | 1.838037 | 1.35E-03 | 1.89E-02 | HSPA1A/PIK3R1/NOVA1/PSIP1/AHNAK/MBNL1/HSPA8/AHNAK2/DDX5/HNRNPA2B1/CDC5L/HNRNPU/SYF2/IVNS1ABP/SON/PABPC1/PRPF4B/HNRNPA3/SCAF11/TRA2B/CELF2/RBM39/FUS/YTHDC1/RBMX/LGALS3/IK/CWF19L2/PCF11/HNRNPH3/SRSF3/SAP18/SRRM2/SNW1/PNN/SRSF7/FRG1/SRRM1/U2SURP/FAM172A/QKI/C9orf78/KHDRBS1/SRSF10/SMNDC1/SLU7/HNRNPK/DNAJC8/CRNKL1/DHX40/PAPOLA/GTF2F1/SREK1IP1/ZC3H13/PRPF6/RBM8A/PPIG/MBNL2/HNRNPC/CIR1/ZNF638/HNRNPR/REST/CDK13/HNRNPL/RBM22/SFPQ/SF3B2/VIRMA/ZCCHC8/WBP4/C2orf49/ZBTB7A/LUC7L2/PRPF38B/DHX9/DDX46/HNRNPA0/HTATSF1/PCBP2/ACIN1/MTREX/RBM25/PRPF8/MPHOSPH10/AQR/NUDT21/SRSF2/YBX1/HNRNPM/RSRC1/THRAP3/RNPC3/RRAGC/HNRNPUL1/NSRP1/CDK12/SF3B1/SMU1/HABP4/TGS1/SNRNP27/CWC15/ZNF830/MFAP1/DDX1/CLK1/RBM17/WBP11/ZRSR2/SYNCRIP/ZMAT2/HNRNPA1/CDC40/PPP2CA/TRA2A/CWC22/PRPF38A/METTL14/RBM28/RBM7/HNRNPD/DHX15/CWC25/SRSF4/FIP1L1/SNIP1/SNRNP200/FAM98B/TMBIM6/PRPF40A/DYRK1A/FMR1/METTL16/DDX39B |
| regulation of microtubule-based process | 1.834035 | 1.49E-03 | 1.89E-02 | HSPA1B/HSPA1A/STAG2/HNRNPU/TPR/DIXDC1/MAP1A/VPS4B/MAP1B/CDKN1B/SMC3/AKAP9/MACF1/CHORDC1/SMC1A/DIAPH1/GPSM2/APC/ROCK2/PLK2/RHOA/LAMP1/SENP6/NIN/KAT2B/CHMP4B/NUMA1/XPO1/PAFAH1B1/PDCD6IP/CLTC/CDK5RAP2/CHMP3/MID1IP1/SLAIN2/MAPRE2/STAG1/FKBP4/CHMP2B/TAOK1/CYLD/RAC1/MECP2/TERF1/DYNC1H1/CAMSAP2/CHMP1B/BICD1/PRKAA1/CDK2AP2/DYRK1A/IGBP1/MCPH1/CLIP1/TTBK2 |
| regulation of microtubule cytoskeleton organization | 1.825021 | 1.56E-03 | 1.89E-02 | HSPA1B/HSPA1A/STAG2/HNRNPU/TPR/DIXDC1/MAP1A/VPS4B/MAP1B/CDKN1B/SMC3/AKAP9/CHORDC1/SMC1A/GPSM2/APC/ROCK2/PLK2/RHOA/SENP6/NIN/KAT2B/CHMP4B/NUMA1/XPO1/PAFAH1B1/PDCD6IP/CLTC/CDK5RAP2/CHMP3/MID1IP1/SLAIN2/MAPRE2/STAG1/FKBP4/CHMP2B/TAOK1/CYLD/RAC1/MECP2/TERF1/DYNC1H1/CAMSAP2/CHMP1B/BICD1/PRKAA1/CDK2AP2/DYRK1A/MCPH1/CLIP1/TTBK2 |
| regulation of organelle assembly | 1.774614 | 1.50E-03 | 1.89E-02 | HSPA1B/GSN/HSPA1A/EZR/STAG2/PTPRD/SYNE2/HNRNPU/TPR/MSN/MAP4/VPS4B/AKAP13/SMC3/SMC1A/CCDC88A/GPSM2/PLK2/RHOA/SENP6/DYNLL1/KAT2B/CHMP4B/NUMA1/G3BP2/RDX/PDCD6IP/CDK5RAP2/NUPR1/RAB3GAP2/DYNC2LI1/GAP43/STAG1/CHMP2B/G3BP1/CYLD/ATXN2 |
| intrinsic apoptotic signaling pathway | 1.7672 | 1.45E-03 | 1.89E-02 | CLU/HSPA1A/S100A8/CRIP1/PIK3R1/S100A9/DDIT4/CAV1/DNAJA1/HSPB1/DDX3X/SOD1/ARL6IP5/CDKN1A/CD44/DDX5/IVNS1ABP/NFE2L2/ACKR3/SOD2/SKIL/SNW1/PRKDC/BCLAF1/CD24/YBX3/SIRT1/HNRNPK/PPP1R15A/PTTG1IP/DNAJC10/BAG5/CUL3/TOPORS/CYP1B1/CUL5/DNM1L/BCL2/MSH6/USP47/EP300/PDCD10/SFPQ/STK24/DYRK2/SERINC3/ATM/DDIT3/ING2/FBXW7/NUPR1/JAK2/HINT1/ATF4/URI1/MDM2/SNAI1/UBE2K/CASP3/UBB/CYLD |
| RNA splicing, via transesterification reactions with bulged adenosine as nucleophile | 1.761532 | 1.36E-03 | 1.89E-02 | NOVA1/PSIP1/MBNL1/HSPA8/DDX5/HNRNPA2B1/CDC5L/HNRNPU/SYF2/SON/PABPC1/PRPF4B/HNRNPA3/SCAF11/TRA2B/CELF2/FUS/YTHDC1/RBMX/IK/CWF19L2/PCF11/HNRNPH3/SRSF3/SAP18/SRRM2/SNW1/PNN/SRSF7/FRG1/SRRM1/U2SURP/FAM172A/C9orf78/KHDRBS1/SRSF10/SMNDC1/SLU7/HNRNPK/DNAJC8/CRNKL1/DHX40/PAPOLA/GTF2F1/PRPF6/RBM8A/MBNL2/HNRNPC/HNRNPR/REST/CDK13/HNRNPL/RBM22/SFPQ/SF3B2/ZCCHC8/WBP4/ZBTB7A/LUC7L2/DHX9/DDX46/HNRNPA0/HTATSF1/PCBP2/MTREX/RBM25/PRPF8/AQR/NUDT21/SRSF2/YBX1/HNRNPM/RSRC1/THRAP3/RNPC3/HNRNPUL1/NSRP1/SF3B1/SMU1/TGS1/SNRNP27/CWC15/MFAP1/DDX1/RBM17/WBP11/ZRSR2/SYNCRIP/ZMAT2/HNRNPA1/CDC40/TRA2A/CWC22/PRPF38A/METTL14/RBM7/HNRNPD/DHX15/CWC25/SRSF4/FIP1L1/SNIP1/SNRNP200/PRPF40A/DYRK1A/FMR1/METTL16/DDX39B |
| mRNA splicing, via spliceosome | 1.761532 | 1.36E-03 | 1.89E-02 | NOVA1/PSIP1/MBNL1/HSPA8/DDX5/HNRNPA2B1/CDC5L/HNRNPU/SYF2/SON/PABPC1/PRPF4B/HNRNPA3/SCAF11/TRA2B/CELF2/FUS/YTHDC1/RBMX/IK/CWF19L2/PCF11/HNRNPH3/SRSF3/SAP18/SRRM2/SNW1/PNN/SRSF7/FRG1/SRRM1/U2SURP/FAM172A/C9orf78/KHDRBS1/SRSF10/SMNDC1/SLU7/HNRNPK/DNAJC8/CRNKL1/DHX40/PAPOLA/GTF2F1/PRPF6/RBM8A/MBNL2/HNRNPC/HNRNPR/REST/CDK13/HNRNPL/RBM22/SFPQ/SF3B2/ZCCHC8/WBP4/ZBTB7A/LUC7L2/DHX9/DDX46/HNRNPA0/HTATSF1/PCBP2/MTREX/RBM25/PRPF8/AQR/NUDT21/SRSF2/YBX1/HNRNPM/RSRC1/THRAP3/RNPC3/HNRNPUL1/NSRP1/SF3B1/SMU1/TGS1/SNRNP27/CWC15/MFAP1/DDX1/RBM17/WBP11/ZRSR2/SYNCRIP/ZMAT2/HNRNPA1/CDC40/TRA2A/CWC22/PRPF38A/METTL14/RBM7/HNRNPD/DHX15/CWC25/SRSF4/FIP1L1/SNIP1/SNRNP200/PRPF40A/DYRK1A/FMR1/METTL16/DDX39B |
| protein folding | 1.761044 | 1.44E-03 | 1.89E-02 | CLU/CRYAB/HSPA1B/DNAJB1/HSPA1A/HSP90AA1/HSP90AB1/HSPA6/HSPH1/DNAJA1/LTBP4/HSPB1/HSPD1/FKBP5/HSPA8/HSPE1/DNAJB4/HSP90B1/BAG3/ST13/CANX/HSPA5/PDIA3/CHORDC1/PTGES3/TTC1/TCP1/HSPA2/DNAJC3 |
| RNA splicing, via transesterification reactions | 1.760615 | 1.36E-03 | 1.89E-02 | NOVA1/PSIP1/MBNL1/HSPA8/DDX5/HNRNPA2B1/CDC5L/HNRNPU/SYF2/SON/PABPC1/PRPF4B/HNRNPA3/SCAF11/TRA2B/CELF2/FUS/YTHDC1/RBMX/IK/CWF19L2/PCF11/HNRNPH3/SRSF3/SAP18/SRRM2/SNW1/PNN/SRSF7/FRG1/SRRM1/U2SURP/FAM172A/C9orf78/KHDRBS1/SRSF10/SMNDC1/SLU7/HNRNPK/DNAJC8/CRNKL1/DHX40/PAPOLA/GTF2F1/PRPF6/RBM8A/MBNL2/HNRNPC/HNRNPR/REST/CDK13/HNRNPL/RBM22/SFPQ/SF3B2/ZCCHC8/WBP4/ZBTB7A/LUC7L2/DHX9/DDX46/HNRNPA0/HTATSF1/PCBP2/MTREX/RBM25/PRPF8/MPHOSPH10/AQR/NUDT21/SRSF2/YBX1/HNRNPM/RSRC1/THRAP3/RNPC3/HNRNPUL1/NSRP1/SF3B1/SMU1/TGS1/SNRNP27/CWC15/MFAP1/DDX1/RBM17/WBP11/ZRSR2/SYNCRIP/ZMAT2/HNRNPA1/CDC40/TRA2A/CWC22/PRPF38A/METTL14/RBM7/HNRNPD/DHX15/CWC25/SRSF4/FIP1L1/SNIP1/SNRNP200/PRPF40A/DYRK1A/FMR1/METTL16/DDX39B |
| DNA conformation change | 1.753171 | 1.46E-03 | 1.89E-02 | DDX3X/BRD2/ANXA1/HNRNPA2B1/CHD1/TPR/UBC/SOX9/HMGB1/HP1BP3/ASH1L/SET/ANP32B/XRCC5/SPTY2D1/RSF1/CHD9/UBA52/SMARCA5/NAP1L4/DHX36/NASP/NIPBL/TTN/RECQL/ATRX/SMCHD1/PHF13/MNAT1/TOP1/CHD4/CENPC/PURA/RBBP4/DHX9/ACIN1/RBBP7/UBB/XPC/G3BP1/XRCC6/DDX1/WAPL/SMARCAD1/NBN/RAD23B/KAT6A/HMGB2/NAP1L1/MRE11/MCM7/KAT6B/RAD50/MCPH1/TOP2B/INO80/UBN1/CTCF/WRN/TSPYL4/CHD8/POLE3/DDB2/GTF2H1/CENPX/SHPRH/SETX/TSPYL2/RNF8/XPA/CETN2/CHD2/SMARCA1/WRNIP1/SRPK1/ASF1A/PAF1/PARP1/CUL4B |

**Table S5 GSVA enrichment analysis of CHAD^+^ chondrocyte-like cells in the ligament tissue.**

| Description | NES | pvalue | qvalues | Gene |
| --- | --- | --- | --- | --- |
| leukocyte mediated immunity | -1.60353 | 1.52E-03 | 1.33E-02 | DNASE1/DLG1/JUP/SERPINB9/C1R/STXBP2/ROCK1/MAP3K7/PDXK/GMFG/COTL1/TXNDC5/CYFIP1/CRK/HGSNAT/PTPRN2/FTL/ITGB2/ANXA3/PYGL/AZU1/GRN/MPO/FCER1G/SYNGR1/FCN1/MAPK14/BRI3/MAPK1/CTSS/VAMP2/METTL7A/CFI/RAB31/RAC2/SLC44A2/C1RL/SERPINB6/GLIPR1/PTPRC/EMP2/NPC2/CYBA/NA/PGLYRP1/DEFA4/RETN/TYROBP/CDK13/CNN2/MAVS/SVIP/NA/LCN2/BST2/IGLL5/C1S/MNDA/IQGAP2/ELANE/C7/CAMP/MMP9/NA/LYZ/LTF/NA/S100A12/S100A9/NA/S100A8/NA/NA/NA |
| response to biotic stimulus | -1.49199 | 1.52E-03 | 1.33E-02 | CNOT7/BCL2L11/BAIAP2/TNFRSF14/SERPINB9/CREBZF/IFI44L/ERBIN/G3BP1/PABPN1/COTL1/ANKRD17/CRK/ADARB1/TPT1/ANXA3/PYGL/AZU1/GRN/TRIM44/MPO/FCER1G/MAPK14/GBP3/SCN7A/SERINC5/MAPK1/TNFRSF1A/BMP2/IL17RA/GJA1/BNIP3/PDCD4/LITAF/FLNA/SAMHD1/IL33/JCHAIN/PTPRC/TIMP4/STAT2/TRIM38/NPC2/NR1H3/CYBA/NA/IFITM3/FKBP5/PGLYRP1/DEFA4/SEC14L1/IRF3/TRIM56/HMGB2/TFPI/MAVS/NA/LCN2/CFLAR/BST2/IGLL5/ELANE/CXCL8/CAMP/NA/ABCA1/F2R/DDX17/IFITM2/IFITM1/LYZ/LTF/TXNIP/NA/DEFA3/S100A12/S100A9/NA/S100A8/NA/NA/NA |
| innate immune response | -1.63601 | 1.52E-03 | 1.33E-02 | CNOT7/FBXO9/PSMB4/TNFRSF14/SERPINB9/XIAP/C1R/STXBP2/MAP3K7/ERBIN/G3BP1/ICAM3/ANKRD17/CRK/TRIM23/ADARB1/PTPRS/PSMB8/ITGB2/MAP4K2/CAMK2D/GRN/IL1RAP/FCER1G/CREBBP/FCN1/TAB3/GBP3/CASP4/XAF1/SERINC5/CTSS/NCF1/PARP14/CFI/APPL1/TRIM27/SAMHD1/C1RL/PIAS1/JCHAIN/TRIM13/STAT2/TRIM38/NR1H3/CYBA/NA/IFITM3/PGLYRP1/DEFA4/SEC14L1/TYROBP/IRF3/TRIM56/HMGB2/MAVS/NA/LCN2/NCAM1/BST2/IGLL5/C1S/MNDA/C7/CAMP/NA/APOE/IFITM2/IFITM1/LTF/NA/A2M/DEFA3/S100A12/S100A9/SAA1/NA/S100A8/NA/NA/NA |
| response to external biotic stimulus | -1.50662 | 1.53E-03 | 1.33E-02 | CNOT7/BCL2L11/BAIAP2/TNFRSF14/SERPINB9/CREBZF/IFI44L/ERBIN/G3BP1/PABPN1/COTL1/ANKRD17/CRK/ADARB1/TPT1/ANXA3/PYGL/AZU1/GRN/TRIM44/MPO/FCER1G/MAPK14/GBP3/SCN7A/SERINC5/MAPK1/TNFRSF1A/BMP2/IL17RA/GJA1/BNIP3/PDCD4/LITAF/FLNA/SAMHD1/IL33/JCHAIN/PTPRC/TIMP4/STAT2/TRIM38/NPC2/NR1H3/CYBA/NA/IFITM3/FKBP5/PGLYRP1/DEFA4/SEC14L1/IRF3/TRIM56/HMGB2/TFPI/MAVS/NA/LCN2/CFLAR/BST2/IGLL5/ELANE/CXCL8/CAMP/NA/ABCA1/F2R/DDX17/IFITM2/IFITM1/LYZ/LTF/NA/DEFA3/S100A12/S100A9/NA/S100A8/NA/NA/NA |
| response to other organism | -1.50662 | 1.53E-03 | 1.33E-02 | CNOT7/BCL2L11/BAIAP2/TNFRSF14/SERPINB9/CREBZF/IFI44L/ERBIN/G3BP1/PABPN1/COTL1/ANKRD17/CRK/ADARB1/TPT1/ANXA3/PYGL/AZU1/GRN/TRIM44/MPO/FCER1G/MAPK14/GBP3/SCN7A/SERINC5/MAPK1/TNFRSF1A/BMP2/IL17RA/GJA1/BNIP3/PDCD4/LITAF/FLNA/SAMHD1/IL33/JCHAIN/PTPRC/TIMP4/STAT2/TRIM38/NPC2/NR1H3/CYBA/NA/IFITM3/FKBP5/PGLYRP1/DEFA4/SEC14L1/IRF3/TRIM56/HMGB2/TFPI/MAVS/NA/LCN2/CFLAR/BST2/IGLL5/ELANE/CXCL8/CAMP/NA/ABCA1/F2R/DDX17/IFITM2/IFITM1/LYZ/LTF/NA/DEFA3/S100A12/S100A9/NA/S100A8/NA/NA/NA |
| regulation of defense response | -1.64673 | 1.55E-03 | 1.33E-02 | RBM14/TAB1/ARRB2/PTPN2/VPS35/GPX4/CD200/IL6ST/UBA52/MGLL/CNOT7/DNASE1/PSMB4/SERPINB9/XIAP/C1R/MAP3K7/ERBIN/ICAM3/ANKRD17/CRK/PTPRS/PSMB8/ITGB2/ASH1L/GRN/PTGIS/TRIM44/FCER1G/CREBBP/FCN1/MAPK14/TAB3/ATM/CTSS/RICTOR/TNFRSF1A/PARP14/IL17RA/CFI/GJA1/APPL1/PDCD4/SAMHD1/PIAS1/IL33/NUPR1/PTPRC/TRIM38/NR1H3/CYBA/NA/EGFR/PGLYRP1/SEC14L1/IRF3/HMGB2/MAVS/PROS1/C1S/MNDA/ELANE/C7/MMP9/NA/MDK/APOE/LTF/A2M/NEAT1/S100A12/S100A9/SAA1/NA/S100A8/NA/NA/NA/APOD |
| immune response-activating signal transduction | -1.63786 | 1.55E-03 | 1.33E-02 | FYB1/UBA52/HLA-DRA/BAIAP2/PSMB4/XIAP/MAP3K7/ERBIN/ICAM3/ANKRD17/CYFIP1/CRK/PIK3R1/PTPRS/PSMB8/ITGB2/WIPF2/FCER1G/CREBBP/FCN1/TAB3/CBFB/MAPK1/CTSS/APPL1/PTPRC/NR1H3/CYBA/NA/PGLYRP1/SEC14L1/IRF3/NA/IGLL5/MNDA/NA/LTF/NA/S100A9/NA/S100A8/NA/NA/NA |
| regulation of response to external stimulus | -1.6431 | 1.56E-03 | 1.33E-02 | ASH1L/GRN/PTGIS/ZNF580/TRIM44/FCER1G/NBL1/MAPK14/PTN/STK24/ATM/RICTOR/TNFRSF1A/IL17RA/CFI/GJA1/RAC2/APPL1/PDCD4/IL33/NUPR1/PTPRC/PTEN/TRIM38/NR1H3/CYBA/NA/EGFR/PGLYRP1/SEC14L1/IRF3/FGFR1/TFPI/MAVS/PROS1/SLIT2/TSPAN8/C1S/MET/ELANE/CXCL8/C7/THBS1/MMP9/NA/MDK/APOE/F2R/LTF/A2M/NEAT1/S100A12/S100A9/SAA1/NA/S100A8/NA/NA/NA/APOD |
| inflammatory response | -1.64754 | 1.58E-03 | 1.33E-02 | ITGB2/LXN/AZU1/ASH1L/GRN/PTGIS/ZNF580/IL1RAP/FCER1G/MAPK14/PTN/KDM6B/ATM/CASP4/RICTOR/TNFRSF1A/BMP2/IL17RA/CFI/GJA1/APPL1/PDCD4/IL33/NUPR1/PTPRC/NR1H3/CYBA/NA/EGFR/PGLYRP1/TYROBP/IRF3/HMGB2/GGT5/PROS1/C1S/ELANE/CXCL8/C7/THBS1/MMP9/NA/MDK/APOE/F2R/LYZ/A2M/NEAT1/S100A12/S100A9/SAA1/NA/S100A8/NA/NA/NA/APOD |
| response to bacterium | -1.58785 | 1.60E-03 | 1.33E-02 | SCN7A/MAPK1/TNFRSF1A/BMP2/GJA1/BNIP3/PDCD4/LITAF/JCHAIN/TIMP4/NR1H3/CYBA/NA/FKBP5/PGLYRP1/DEFA4/IRF3/HMGB2/TFPI/MAVS/NA/LCN2/CFLAR/IGLL5/ELANE/CXCL8/CAMP/NA/ABCA1/F2R/LYZ/LTF/NA/DEFA3/S100A12/S100A9/NA/S100A8/NA/NA/NA |
| defense response to other organism | -2.22632 | 1.61E-03 | 1.33E-02 | TNFRSF14/IFI44L/G3BP1/COTL1/ANKRD17/ADARB1/ANXA3/AZU1/GRN/TRIM44/MPO/FCER1G/GBP3/SERINC5/TNFRSF1A/IL17RA/BNIP3/FLNA/SAMHD1/IL33/JCHAIN/PTPRC/STAT2/TRIM38/CYBA/NA/IFITM3/PGLYRP1/DEFA4/SEC14L1/IRF3/TRIM56/HMGB2/MAVS/NA/LCN2/BST2/IGLL5/ELANE/CAMP/NA/DDX17/IFITM2/IFITM1/LYZ/LTF/NA/DEFA3/S100A12/S100A9/NA/S100A8/NA/NA/NA |
| phagocytosis | -1.97909 | 1.64E-03 | 1.33E-02 | ICAM3/TXNDC5/CYFIP1/CRK/PIK3R1/ITGB2/ANXA3/AZU1/WIPF2/FCER1G/FCN1/MAPK1/RAB31/APPL1/PTPRC/PTEN/NR1H3/CYBA/NA/TYROBP/NA/IGLL5/MET/ELANE/THBS1/NA/ABCA1/NA/LEPR/NA/NA/NA/NA |
| leukocyte migration | -1.83108 | 1.64E-03 | 1.33E-02 | SLC3A2/MYH9/SDC3/PREX1/CD99L2/TNFRSF14/SLC7A8/ROCK1/MTUS1/CRK/PIK3R1/ITGA1/ITGB2/ANGPT1/AZU1/ZNF580/FCER1G/NBL1/EPS8/MAPK14/PTN/GLG1/IL17RA/RAC2/CSF3R/BMP5/IL33/JCHAIN/MMP14/ATP1B1/NA/PROS1/SLIT2/ELANE/CXCL8/THBS1/MDK/NA/S100A12/S100A9/SAA1/S100A8/NA/NA/APOD |
| regulation of immune effector process | -1.68851 | 1.64E-03 | 1.33E-02 | DNASE1/TNFRSF14/SERPINB9/C1R/STXBP2/MAP3K7/ANKRD17/CRK/HLX/ITGB2/ANGPT1/GRN/TRIM44/FCER1G/MAPK14/MZB1/CFI/RAC2/APPL1/IL33/PTPRC/TRIM38/NA/PGLYRP1/SEC14L1/MAVS/PROS1/BST2/C1S/C7/NA/A2M/NA/NA/NA/NA |
| immune response-activating cell surface receptor signaling pathway | -1.66399 | 1.64E-03 | 1.33E-02 | TAB1/PTPN2/FYB1/HLA-DRA/BAIAP2/PSMB4/MAP3K7/ICAM3/CYFIP1/CRK/PIK3R1/PSMB8/WIPF2/FCER1G/CREBBP/FCN1/TAB3/CBFB/MAPK1/APPL1/PTPRC/NA/NA/IGLL5/MNDA/NA/NA/NA/NA/NA/NA |
| response to transforming growth factor beta | 1.856261 | 2.44E-03 | 1.33E-02 | FOS/JUN/CILP/ASPN/DKK3/HTRA1/CLEC3B/CD109/CAV1/UBC/FMOD/HSPA5/HSP90AB1/LEFTY2/SOX9/HSPA1A/UBB/SFRP1/RHOA/ID1/COL1A2/COL3A1/MSTN/BAMBI/NR3C1/SOX5/LTBP4/USP15/CITED2/SIRT1/HTRA3/SKI/CAV2/ADAM9/ADAMTSL2/USP9X/BMPR1B/TGFBR2/PPM1A |
| regulation of leukocyte cell-cell adhesion | 1.711623 | 2.45E-03 | 1.33E-02 | KLF4/DPP4/CCL2/ANXA1/HES1/CD55/IRF1/NFKBIZ/CAV1/XBP1/CEBPB/RHOA/GPNMB/LGALS3/LGALS1/CDC42/CD44/CD81/ICAM1/ZC3H12A/HSPH1/CD24/PRDX2/MAP3K8/TNFSF9/BCL6/IGF1/THY1/FGL2/IL6/TGFBR2/SOCS5/PELI1/HSPD1/ASS1/GRB2/SELENOK/TFRC/PNP/FYN/CD70/SOCS1 |
| regulation of T cell activation | 1.702483 | 2.45E-03 | 1.33E-02 | GSN/DPP4/CCL2/ANXA1/HES1/CD55/IRF1/NFKBIZ/CAV1/XBP1/CEBPB/RHOA/GPNMB/LGALS3/LGALS1/CDC42/CD81/NFATC2/ZC3H12A/HSPH1/CD24/PRDX2/MAP3K8/TNFSF9/BCL6/IGF1/THY1/FGL2/IL6/TGFBR2/SOCS5/PELI1/SOD1/HSPD1/GRB2/SELENOK/TFRC/PNP/FYN/CD70/SOCS1 |
| fat cell differentiation | 1.89971 | 2.46E-03 | 1.33E-02 | ZFP36/KLF4/C1QTNF3/SFRP2/NR4A1/HES1/ID2/ADIRF/FRZB/XBP1/CEBPB/SFRP1/NR4A2/RGS2/TCF7L2/CCDC3/CEBPD/ID4/INSIG1/ENPP1/ARID5B/KLF5/ADRB2/LMO3/ZC3H12A/CCDC71L/NR1D1/SIRT1/MEDAG/PTGS2/SYAP1/SDF4/JDP2/IL6/RORA |
| ERK1 and ERK2 cascade | 1.719454 | 2.46E-03 | 1.33E-02 | JUN/PLA2G2A/KLF4/ATF3/DUSP1/ACKR3/CCL2/MYC/EZR/SOX9/GPNMB/CD44/FN1/FGF18/ICAM1/CAMK2N1/PDGFRA/CAVIN3/RRAS/LMO3/ERRFI1 |
| cellular response to extracellular stimulus | 1.709498 | 2.47E-03 | 1.33E-02 | FOS/JUN/ATF3/SFRP2/CDKN1A/HSPA8/KLF10/PDK4/HSP90B1/HSPA5/XBP1/SLC38A2/SFRP1/NR4A2/ATF4/MAP1LC3B/DAP/ICAM1/HIGD1A/ZC3H12A/PIM1/GSTP1/SIRT1 |
| regulation of angiogenesis | 1.817965 | 2.49E-03 | 1.33E-02 | KLF4/RHOB/SERPINE1/KLF2/DCN/SFRP2/ANXA1/RGCC/TNMD/ECM1/PLK2/SERPINF1/AQP1/THBS2/XBP1/ADM/SFRP1/RHOA/PKM/GPNMB/ID1/SRPX2/BTG1/MYDGF/S100A1/CD34/FGF18/SEMA3E/TNFRSF12A/RRAS/SPARC/ZC3H12A/SULF1/SIRT1/VEGFA/HIF1A/PTGS2/IL6 |
| negative regulation of growth | 1.821539 | 2.49E-03 | 1.33E-02 | RERG/MT1M/MT1A/DDX3X/SFRP2/CDKN1A/MT2A/RBP4/FHL1/FRZB/CRYAB/HSPA1B/HSPA1A/SFRP1/BTG1/MT1E/RGS2/ING1/INHBA/ENO1/SEMA3C/ENPP1/SERTAD3/SEMA3E/IFRD1/MSTN/MSX1/ADRB2/NDUFA13/BCL6/CDKN2AIP/SEMA3D/SIRT1/HIF1A/WNT11/NTN1 |
| response to topologically incorrect protein | 2.099403 | 2.50E-03 | 1.33E-02 | CLU/COMP/PPP1R15A/ATF3/DNAJB1/CCL2/HSPA8/DNAJA1/HSP90B1/HSPA5/LMNA/HSP90AB1/XBP1/CALR/HSPA1B/HSPA1A/HSPA2/SELENOS/MYDGF/ATF4/DNAJB4/HERPUD1/BAG3/HSP90AA1/PDIA6/HSPB8/KDELR3 |
| response to temperature stimulus | 1.91904 | 2.50E-03 | 1.33E-02 | FOS/DNAJB1/NFKBIA/IGFBP7/CDKN1A/HSPA8/DNAJA1/HSPA5/HSP90AB1/ADM/CRYAB/HSPA1B/HSPA1A/HSPA2/DNAJB4/RBM3/IER5/BAG3/HSP90AA1/CD34/SCARA5/HSPB8/YWHAE/MSTN/ADRB2/HSPH1/MYOF |
| response to molecule of bacterial origin | 1.775789 | 2.50E-03 | 1.33E-02 | FOS/JUN/JUNB/ZFP36/SERPINE1/MGST1/NFKBIA/JUND/DCN/CXCL2/CCL2/CD55/XBP1/ADM/TSPO/CEBPB/SELENOS/RHOA/VIM/SSC5D/JAK2/ICAM1/CTR9/B2M/SLPI/CXCL3/SPARC/ZC3H12A/CD24/PRDX2/GSTP1/NR1D1/CAPN2 |
| response to unfolded protein | 2.039903 | 2.51E-03 | 1.33E-02 | COMP/PPP1R15A/ATF3/DNAJB1/CCL2/HSPA8/DNAJA1/HSP90B1/HSPA5/LMNA/HSP90AB1/XBP1/CALR/HSPA1B/HSPA1A/HSPA2/SELENOS/MYDGF/ATF4/DNAJB4/HERPUD1/BAG3/HSP90AA1/PDIA6/HSPB8/KDELR3/HSPH1/YIF1A/SEC31A/CREB3L2/SERPINH1/DERL1/SSR1/OPTN/PPP1R15B |
| cellular response to transforming growth factor beta stimulus | 1.878355 | 2.51E-03 | 1.33E-02 | FOS/JUN/CILP/ASPN/DKK3/HTRA1/CLEC3B/CD109/CAV1/UBC/FMOD/HSPA5/HSP90AB1/LEFTY2/SOX9/HSPA1A/UBB/SFRP1/RHOA/ID1/COL1A2/COL3A1/MSTN/BAMBI/NR3C1/SOX5/LTBP4/USP15/CITED2/SIRT1/HTRA3/SKI/CAV2/ADAM9/ADAMTSL2/USP9X/BMPR1B/TGFBR2/PPM1A |
| regulation of ERK1 and ERK2 cascade | 1.677079 | 2.51E-03 | 1.33E-02 | JUN/PLA2G2A/KLF4/ATF3/DUSP1/ACKR3/CCL2/EZR/GPNMB/CD44/FN1/FGF18/ICAM1/CAMK2N1/PDGFRA/CAVIN3/RRAS/LMO3/ERRFI1 |
| connective tissue development | 1.910337 | 2.52E-03 | 1.33E-02 | MGP/EGR1/COMP/CRIP1/LUM/SFRP2/CYTL1/ID2/TNMD/ECM1/FRZB/TIMP1/MATN2/SCARA3/XBP1/SOX9/ANXA2/UBB/SNAI1/CD34/CD44/ID4/ACAN/ZMPSTE24/FGF18/COL6A2/LNPK/ARID5B/CTSK/MSX1/SOX5/SULF1/THBS3/COL14A1/COL12A1/SIRT1/SLC39A13/CREB3L2/SERPINH1/LRP6/HIF1A/WNT11 |

**Table S6 GSVA enrichment analysis of CRTAC1^+^ chondrocyte -like cells in the ligament tissue.**

| Description | NES | pvalue | qvalues | Gene |
| --- | --- | --- | --- | --- |
| sulfur compound biosynthetic process | 1.940535 | 0.002941 | 0.013105 | DCN/PRELP/CDO1/LUM/VCAN/FMOD/UGDH/BGN/PDK4/MGST1/AMD1/DSE/PAPSS2/MGST3/ACAT1/ACSL3/NFE2L2 |
| transforming growth factor beta receptor signaling pathway | 1.814029 | 0.002959 | 0.013105 | ASPN/FMOD/CD109/FBN1/SKIL/ITGB5/CAV1/LTBP2/HTRA1/HSP90AB1/TGFBR3/VASN/DKK3/FERMT2/SIRT1/DAB2/HSPA5/LDLRAD4/FOS/GDF10/BAMBI/ZEB1/ACVR1/SMAD7/LTBP3/RHOA/SNW1/CITED2/ING2/ID1/UBC/USP15/TGFBR1/LEFTY2/ADAM9 |
| connective tissue development | 1.532641 | 0.002985 | 0.013105 | COMP/MGP/LUM/CRIP1/ID2/SOX9/COL12A1/SULF1/EFEMP1/ARID5B/SCX/OSR2/CD44/SIRT1/FGF2/SCARA3/EGR1/SOX5/FGFR1/XBP1/PRRX1/LNPK/ZEB1/ZBTB16/ACAT1/THRA/BMP2/COL6A1/SULF2/CSF1/LTBP3/RARG/CREB3L2/HIF1A/FOXD1/SPART/SFRP2/MSX1/TGFBR1/KLF7/CD34/NFIB/COL6A3/SNAI1/BMPR1A/THBS3/TRPS1/SMAD3/BMPR2/PIK3CA/SIX2 |
| cellular response to transforming growth factor beta stimulus | 1.674806 | 0.002985 | 0.013105 | ASPN/CILP/FMOD/CD109/CLEC3B/SOX9/FBN1/SKIL/ITGB5/CAV1/LTBP2/HTRA1/HSP90AB1/TGFBR3/VASN/DKK3/SCX/FERMT2/SIRT1/DAB2/HSPA5/LDLRAD4/FOS/SOX5/GDF10/BAMBI/ZEB1/NOX4/ACVR1/SMAD7/LTBP3/RHOA/SNW1/CITED2/ING2/ID1/UBC/USP15/TGFBR1/LEFTY2/NR3C1/ADAM9 |
| regulation of transmembrane receptor protein serine/threonine kinase signaling pathway | 1.811935 | 0.003003 | 0.013105 | ASPN/CILP/CD109/FBN1/FSTL1/SKIL/SULF1/CAV1/HTRA1/HSP90AB1/TGFBR3/VASN/DKK3/NBL1/SIRT1/DAB2/HSPA5/LDLRAD4/GDF10/BAMBI/ZEB1/TOB1/ACVR1/BMP2/SMAD7/SNW1/SPTBN1/FOXD1/CITED2/ING2/SPART/UBC/SFRP2/MSX1/TGFBR1/LEFTY2 |
| response to unfolded protein | 1.524926 | 0.00303 | 0.013105 | COMP/THBS4/DNAJA1/ATF3/HSP90AB1/HSPB8/HSPB1/DNAJB4/HSPA8/HERPUD1/OPTN/HSPA5/CREBRF/TLN1/XBP1/BAG3/NFE2L2/PIK3R1/HSPA2/SEC31A/DNAJC3/PPP1R15A/DNAJB9/GFPT1/CREB3L2/SELENOS/CANX/ATF4/DNAJB1/KDELR3/EIF2S1/HSP90B1 |
| regulation of cellular response to growth factor stimulus | 1.621884 | 0.003067 | 0.013105 | ASPN/DCN/CD109/FBN1/FSTL1/SKIL/SULF1/CAV1/HTRA1/HSP90AB1/TGFBR3/VASN/DKK3/NBL1/SIRT1/FGF2/DAB2/HSPA5/LDLRAD4/FGFR1/MYOF/BAMBI/ZEB1/NPTN/TOB1/SMAD7/SULF2/SNW1/HIF1A/FOXD1/CITED2/ING2/SPART/UBC/SFRP2/MSX1/TGFBR1 |
| response to topologically incorrect protein | 1.64927 | 0.003077 | 0.013105 | CLU/COMP/THBS4/DNAJA1/ATF3/HSP90AB1/HSPB8/HSPB1/DNAJB4/HSPA8/HERPUD1/OPTN/HSPA5/CREBRF/TLN1/XBP1/BAG3/NFE2L2/PIK3R1/HSPA2/SEC31A/DNAJC3/PPP1R15A/DNAJB9/GFPT1/CREB3L2/SELENOS/CANX/CUL3/ATF4/DNAJB1/KDELR3/EIF2S1/HSP90B1 |
| response to transforming growth factor beta | 1.681782 | 0.003086 | 0.013105 | ASPN/CILP/FMOD/CD109/CLEC3B/SOX9/FBN1/SKIL/ITGB5/CAV1/LTBP2/HTRA1/HSP90AB1/TGFBR3/VASN/DKK3/SCX/FERMT2/SIRT1/ZFHX3/DAB2/HSPA5/LDLRAD4/FOS/SOX5/GDF10/BAMBI/ZEB1/NOX4/ACVR1/SMAD7/LTBP3/RHOA/SNW1/CITED2/ING2/ID1/UBC/USP15/TGFBR1/LEFTY2/NR3C1/ADAM9 |
| transmembrane receptor protein serine/threonine kinase signaling pathway | 1.954921 | 0.003175 | 0.013298 | COMP/ASPN/CILP/FMOD/CD109/FBN1/FSTL1/SKIL/ITGB5/SULF1/CAV1/LTBP2/HTRA1/HSP90AB1/TGFBR3/VIM/VASN/DKK3/SCX/DDX5/NBL1/FERMT2/SIRT1/EGR1/DAB2/HSPA5/LDLRAD4/FOS/TMEM100/HIVEP1/GDF10/BAMBI/ZEB1/TOB1/ACVR1/BMP2/SMAD7/LTBP3/RHOA/SNW1/SPTBN1/FOXD1/CITED2/ING2/SPART/ID1/UBC/SFRP2/USP15/MSX1/NKX2-5/TGFBR1/LEFTY2/ADAM9 |
| extracellular matrix organization | 1.593163 | 0.003185 | 0.013298 | COMP/ABI3BP/TNFRSF11B/DCN/LUM/VCAN/RGCC/DPP4/TNXB/FMOD/HAS1/DPT/FN1/ECM2/SOX9/BGN/SERPINE1/FBN1/FBLN1/MFAP5/MFAP4/ITGB5/COL12A1/SULF1/PLOD2/HTRA1/TIMP2/FAP/CAPN2/SCX/CD44/ANTXR1/CST3/FGF2/ITGA10/P4HA1/ITGA11/CYP1B1/MMP3/COL8A2/NTN4/PDGFRA/COL8A1/DDR2 |
| sulfur compound metabolic process | 1.659168 | 0.003236 | 0.013403 | DCN/PRELP/CDO1/LUM/VCAN/FMOD/UGDH/BGN/PDK4/MGST1/AMD1/DSE/SOD1/ENPP1/NFE2L1/PAPSS2/MGST3/ACAT1/ACSL3/AHCYL1/NOX4/PCYOX1/NFE2L2/SLC19A2 |
| extracellular structure organization | 1.51656 | 0.003344 | 0.013739 | COMP/ABI3BP/TNFRSF11B/DCN/LUM/VCAN/RGCC/DPP4/TNXB/FMOD/HAS1/PLA2G2A/DPT/FN1/ECM2/SOX9/BGN/SERPINE1/FBN1/FBLN1/MFAP5/MFAP4/ITGB5/COL12A1/SULF1/PLOD2/HTRA1/TIMP2/FAP/CAPN2/SCX/CD44/ANTXR1/CST3/FGF2/ITGA10/P4HA1/ITGA11/CYP1B1/MMP3/COL8A2/NTN4/PDGFRA/COL8A1/DDR2 |
| skeletal system development | 1.559047 | 0.00361 | 0.01471 | COMP/MGP/TNFRSF11B/PRELP/LUM/VCAN/MYOC/PLS3/SOX9/FBN1/RBP4/COL12A1/SULF1/TIPARP/EFEMP1/CHAD/ARID5B/SCX/CLEC3A/OSR2/CD44/FGF2/SCARA3/RAB23/ANKH/SBDS/SOX5/ZNF385A/PAPSS2/GDF10/FGFR1/PDGFRA/PRRX1/LNPK/HOXC10/ZEB1/ZBTB16/ANO6/PTPN11/VKORC1/RYK/INSIG1/PDGFC/THRA/HOXD8/BMP2/CMKLR1/COL6A1/SULF2/LTBP3/HOXC9/RARG/CREB3L2/RHOA/HIF1A/SETD2/PLEKHA1/SFRP2/MSX1/TGFBR1/NOTCH2/NFIB/COL6A3/PBX1/SNAI1/SIX1/HOXA10/IGF1/RAB33B/WDR48/BMPR1A/THBS3/FAT4/TRPS1/FOXN3/SMAD3/BMPR2/PRDX1/SIX2/SFRP4/FOXC1 |
| cellular response to growth factor stimulus | 1.566896 | 0.003861 | 0.015607 | COMP/ASPN/CILP/DCN/FMOD/CD109/HAS1/CLEC3B/KLF4/SOX9/FBN1/FSTL1/SKIL/ITGB5/SULF1/NCL/CAV1/NTRK2/LTBP2/GAS1/HTRA1/HSP90AB1/ERRFI1/ANXA1/TGFBR3/HSPB1/VASN/DKK3/MAP1B/SCX/DDX5/CD44/NBL1/FERMT2/SIRT1/FGF2/IQGAP1/EGR1/DAB2/HSPA5/LDLRAD4/FOS/CDC5L/TMEM100/SOX5/CASP3/HIVEP1/GDF10/FGFR1/PDGFRA/MYOF/BAMBI/ZEB1/PTPN11/FOXO3/SHOC2/NOX4/NPTN/TOB1/ACVR1/BMP2/SMAD7/SULF2/LTBP3/RHOA/SNW1/HIF1A/FOXD1/PTPN12/CITED2/RAB14/CPNE3/ING2/ITGB1BP1/SPART/ID1/UBC/SFRP2/USP15/MSX1/NKX2-5/TGFBR1/LEFTY2/NR3C1 |
| regulated exocytosis | -1.62312 | 0.001316 | 0.009182 | ADAM10/NR4A3/GOLGA7/DEGS1/SNAP23/ARL8A/GYG1/PFKL/PLEKHO2/SERPINB1/CRISPLD2/SLC2A3/SYT11/ACTR2/UBR4/SCRIB/NAPA/TOLLIP/PLAU/PSMB1/PTGES2/PSEN1/GPI/TEX264/ACAA1/SDCBP/HSP90AA1/HEBP2/PRCP/PGAM1/ARPC5/GNAI2/VAMP2/TAGLN2/HGSNAT/PSMD7/CAP1/CPPED1/HSPA6/APRT/PYCARD/ADRA2A/SLC44A2/CD63/TMC6/CALM3/ALDH3B1/CPNE1/PDGFB/NOTCH1/PSMD13/SYNGR2/LRRK2/MIF/RAB11FIP1/RAB11B/TGFB1/OSTF1/DGAT1/RHOG/AGPAT2/STK10/NDUFC2/CLEC12A/IQGAP2/PLAUR/GAA/BRI3/IGF2R/QPCT/LAMTOR1/HSPA1A/VAMP8/TRAPPC1/STXBP2/SVIP/PTX3/VWF/FCGR2B/CNN2/ACTN1/TSPAN14/MAGED2/VCL/FLNA/PLEK/ASAH1/PECAM1/S100P/CYBB/CTSH/SCCPDH/PGLYRP1/GCA/RNASET2/FOLR3/ELANE/A1BG/LCN2/ACTN4/GRN/B2M/RAC2/BST2/SYNGR1/CD59/CTSG/FCER1G/CTSD/A2M/SNCA/CTSS/GMFG/FABP5/TYROBP/CORO1A/MNDA/RETN/MMP9/FTH1/MPO/CAMP/FGL2/PTPRC/COTL1/LGALS3BP/CYBA/CTSC/LTF/RARRES2/S100A12/SPARC/SRGN/LYZ/S100A9/S100A8 |
| innate immune response | -2.07821 | 0.001325 | 0.009182 | IL1RAP/MSRB1/PSMB10/FAM3A/TRIM28/MYO1C/FLNB/IFI16/PSMD7/NOP53/TNFRSF14/ARRB2/PYCARD/CAPZA1/C1RL/SIRT2/PSMD13/ICAM3/PSMB8/C1S/IKBKB/HLA-C/MIF/PSMA4/PSMA3/IFI35/CNPY3/PSMB5/NECTIN2/MUCL1/TGFB1/ZYX/IRF7/PSPC1/IFNAR2/C1QBP/HLA-A/PSMB3/TP53/PSMA1/C1QA/WNT5A/CXCL16/NA/SOCS3/ISG20/HSPA1A/STXBP2/PTX3/PSME2/EVL/FCGR2B/GBP1/IFNGR2/C1R/ISG15/NCAM1/PSMB9/BIRC3/HMGB2/MX2/COL20A1/NCF2/CYBB/SAMHD1/CLDN1/PGLYRP1/IFITM1/LCN2/GRN/HLA-DQA1/B2M/BST2/JCHAIN/FCER1G/LYST/ARID5A/A2M/SNCA/CTSS/HLA-B/NCF1/TYROBP/CORO1A/TNFAIP3/AIF1/MNDA/NA/CAMP/SPON2/CADM1/IFITM3/DEFA3/HLA-DPB1/CYBA/NA/NA/LTF/HLA-DPA1/IFITM2/RARRES2/S100A12/CCL2/CCL19/HLA-DRB1/HLA-DRA/APOE/S100B/NA/NA/S100A9/NA/S100A8/NA |
| cation transport | -1.45972 | 0.001326 | 0.009182 | F2R/TMEM175/BCL2/DLG1/NLGN3/ANXA6/COX7C/SFXN1/ATP5F1B/TPT1/PIEZO1/SLC22A18/SLC25A25/SLC9A3/ATP6V1F/SLC25A3/PTEN/SNAP23/COX6B1/BAX/SYT11/ATP6V1G1/ATG5/CALCRL/ATP5ME/COX5A/RASA3/SRI/PSEN1/BIN1/SLC2A8/SELENON/NKAIN4/MYLK/SLC11A2/CYC1/CTNNB1/PTGS2/ATP6V0E2/GNAI2/CNTN1/VAMP2/SARAF/SLC12A7/SLC22A3/COX5B/ATP6AP1/RAMP1/COX17/MLLT6/ARRB2/ADRA2A/SLC44A2/CD63/SLC33A1/PHB2/SLC12A2/CALM3/ARHGAP1/PDGFB/COX6C/MYO5A/CNKSR3/ATP1A2/ORAI2/CD4/ITPR1/ATP5F1C/SLC44A1/RAB11B/ATP5MC1/TGFB1/ORAI1/AKT1/ATOX1/DDIT3/SLC5A3/NDUFS7/SLC15A3/KCNS3/ATP5MF/COX8A/VAMP8/UCP2/SLC25A29/CXCR4/ATP6V0B/COX4I1/CHCHD10/STEAP4/MAGED2/KCNMB4/FLNA/NDUFA4/ATP1B1/ATP6V0D1/FKBP1A/CYB5A/ATP5MC2/ATP5F1D/FXYD1/ATP5MC3/RELN/SCN9A/ATP5MG/DMD/ATP5F1E/ACTN4/B2M/FCER1G/MEF2C/TTYH1/SNCA/CTSS/CXCL12/THY1/CORO1A/MMP9/FTH1/PTPRC/CYBA/PRNP/SCN7A/CCL2/CCL19/GPM6B |
| cell activation involved in immune response | -1.77501 | 0.00133 | 0.009182 | ADAM10/NR4A3/GOLGA7/DEGS1/SNAP23/ARL8A/GYG1/PFKL/PLEKHO2/SERPINB1/CRISPLD2/SLC2A3/ACTR2/UBR4/LOXL3/TOLLIP/PLAU/PSMB1/PTGES2/MAD2L2/PSEN1/GPI/ACAA1/SDCBP/HSP90AA1/HEBP2/PRCP/PGAM1/ARPC5/ERCC1/VAMP2/HGSNAT/PSMD7/CAP1/CPPED1/HSPA6/APRT/PYCARD/CD81/SLC44A2/CD63/TMC6/ALDH3B1/CPNE1/PSMD13/MIF/TGFB1/OSTF1/DGAT1/TP53/RHOG/AGPAT2/STK10/NDUFC2/CLEC12A/IQGAP2/PLAUR/GAA/BRI3/IGF2R/QPCT/LAMTOR1/HSPA1A/VAMP8/TRAPPC1/STXBP2/SVIP/PTX3/FCGR2B/CNN2/RARA/TSPAN14/VCL/LGALS1/ASAH1/PECAM1/S100P/CYBB/CTSH/MDK/PGLYRP1/GCA/RNASET2/FOLR3/ELANE/A1BG/LCN2/GRN/B2M/RAC2/BST2/SYNGR1/CD59/CTSG/FCER1G/CTSD/CTSS/GMFG/FABP5/TYROBP/CORO1A/MNDA/RETN/MMP9/LCP1/FTH1/MPO/CAMP/FGL2/PTPRC/COTL1/CYBA/TCIM/CTSC/LTF/S100A12/CCL19/LYZ/S100A9/S100A8 |
| response to biotic stimulus | -1.94597 | 0.00133 | 0.009182 | FAM3A/JUND/IFI16/PRKRA/BANF1/DGKB/HYAL2/PDCD4/NOP53/TNFRSF14/AIMP1/PYCARD/SIRT2/CDK4/NOTCH1/LITAF/IKBKB/MIF/CD4/NECTIN2/TGFB1/IRF7/IFNAR2/TNFRSF1A/C1QBP/SIGIRR/TP53/AKT1/WNT5A/COMT/DDIT3/ZFP36/CFL1/NA/ISG20/RGS1/PTX3/CARD8/CCDC80/FCGR2B/GBP1/PABPN1/IFNGR2/CARD16/CDK6/CXCR4/RARA/ABCA1/ISG15/IFI44/BIRC3/HMGB2/HMGA1/FLNA/MX2/SAMHD1/CLDN1/DDX17/TIMP4/PGLYRP1/CFLAR/ELANE/IFITM1/LCN2/GRN/ACTA2/DCD/B2M/BST2/JCHAIN/CTSG/FCER1G/MEF2C/LYST/SNCA/EDNRB/CNP/CXCL12/TNFAIP3/CXCL8/IL1B/NA/MPO/CAMP/FGL2/SPON2/IFITM3/DEFA3/PTPRC/COTL1/STMN1/CYBA/NA/NA/LTF/IFITM2/SCN7A/RARRES2/S100A12/CCL2/SPARC/CCL19/LYZ/NA/NA/S100A9/NA/S100A8/NA |
| regulation of proteolysis | -1.52031 | 0.00133 | 0.009182 | ARRB2/PLGRKT/PYCARD/MTCH1/ADRA2A/CD81/SIRT2/DNAJB2/PDCD6/TNFAIP8/ADRM1/PSMB8/C1S/LRRK2/TMEM259/SGTA/STUB1/PSMA3/PTK2/TRIB2/NDUFA13/DNAJB6/SPINT2/AURKAIP1/CRIM1/CSNK1E/PRELID1/C1QBP/TP53/AKT1/HIPK2/C1QA/ATP5IF1/RBX1/PLAUR/HSPE1/NA/RHBDF1/HSPA1A/SVIP/CARD8/PSME2/CARD16/MMP14/C1R/F3/PSMB9/BIRC3/PCOLCE/CTSH/TIMP4/PEBP1/CFLAR/GRN/CSTA/ITIH5/BST2/SOX2/CD59/CTSD/PI16/A2M/SNCA/COL7A1/NGFR/IL1B/BEX3/COL28A1/MMP9/PLAT/NA/NA/PRNP/CTSC/LTF/APOE/NA/NA/S100A9/NA/S100A8/NA |
| response to external biotic stimulus | -1.96729 | 0.00133 | 0.009182 | FAM3A/JUND/IFI16/PRKRA/BANF1/DGKB/HYAL2/PDCD4/NOP53/TNFRSF14/AIMP1/PYCARD/SIRT2/CDK4/NOTCH1/LITAF/IKBKB/MIF/CD4/TGFB1/IRF7/IFNAR2/TNFRSF1A/C1QBP/SIGIRR/AKT1/WNT5A/COMT/ZFP36/CFL1/NA/ISG20/RGS1/PTX3/CARD8/CCDC80/FCGR2B/GBP1/PABPN1/IFNGR2/CARD16/CDK6/CXCR4/RARA/ABCA1/ISG15/IFI44/BIRC3/HMGB2/HMGA1/FLNA/MX2/SAMHD1/CLDN1/DDX17/TIMP4/PGLYRP1/CFLAR/ELANE/IFITM1/LCN2/GRN/ACTA2/DCD/B2M/BST2/JCHAIN/CTSG/FCER1G/MEF2C/LYST/SNCA/EDNRB/CNP/CXCL12/TNFAIP3/CXCL8/IL1B/NA/MPO/CAMP/FGL2/SPON2/IFITM3/DEFA3/PTPRC/COTL1/STMN1/CYBA/NA/NA/LTF/IFITM2/SCN7A/RARRES2/S100A12/CCL2/SPARC/CCL19/LYZ/NA/NA/S100A9/NA/S100A8/NA |
| response to other organism | -1.96729 | 0.00133 | 0.009182 | FAM3A/JUND/IFI16/PRKRA/BANF1/DGKB/HYAL2/PDCD4/NOP53/TNFRSF14/AIMP1/PYCARD/SIRT2/CDK4/NOTCH1/LITAF/IKBKB/MIF/CD4/TGFB1/IRF7/IFNAR2/TNFRSF1A/C1QBP/SIGIRR/AKT1/WNT5A/COMT/ZFP36/CFL1/NA/ISG20/RGS1/PTX3/CARD8/CCDC80/FCGR2B/GBP1/PABPN1/IFNGR2/CARD16/CDK6/CXCR4/RARA/ABCA1/ISG15/IFI44/BIRC3/HMGB2/HMGA1/FLNA/MX2/SAMHD1/CLDN1/DDX17/TIMP4/PGLYRP1/CFLAR/ELANE/IFITM1/LCN2/GRN/ACTA2/DCD/B2M/BST2/JCHAIN/CTSG/FCER1G/MEF2C/LYST/SNCA/EDNRB/CNP/CXCL12/TNFAIP3/CXCL8/IL1B/NA/MPO/CAMP/FGL2/SPON2/IFITM3/DEFA3/PTPRC/COTL1/STMN1/CYBA/NA/NA/LTF/IFITM2/SCN7A/RARRES2/S100A12/CCL2/SPARC/CCL19/LYZ/NA/NA/S100A9/NA/S100A8/NA |
| leukocyte mediated immunity | -1.96006 | 0.001333 | 0.009182 | ADAM17/ADAM10/NR4A3/GOLGA7/DEGS1/SNAP23/ARL8A/GYG1/PFKL/PLEKHO2/SERPINB1/CRISPLD2/SLC2A3/ACTR2/UBR4/TOLLIP/PLAU/PSMB1/PTGES2/MAD2L2/PSEN1/GPI/ACAA1/SDCBP/HSP90AA1/HEBP2/PRCP/PGAM1/ARPC5/ERCC1/VAMP2/HGSNAT/PSMD7/CAP1/CPPED1/HSPA6/APRT/ARRB2/PYCARD/CD81/C1RL/SLC44A2/CD63/TMC6/ALDH3B1/CPNE1/PSMD13/C1S/HLA-C/MIF/NECTIN2/TGFB1/IRF7/OSTF1/DGAT1/C1QBP/HLA-A/RHOG/JAG1/AGPAT2/STK10/NDUFC2/C1QA/CLEC12A/IQGAP2/PLAUR/GAA/BRI3/IGF2R/QPCT/IL7R/NA/LAMTOR1/HSPA1A/VAMP8/TRAPPC1/STXBP2/SVIP/PTX3/FCGR2B/CNN2/C1R/TSPAN14/VCL/ASAH1/PECAM1/S100P/CYBB/CTSH/PGLYRP1/GCA/RNASET2/FOLR3/ELANE/A1BG/LCN2/GRN/B2M/RAC2/BST2/SYNGR1/CD59/CTSG/FCER1G/CTSD/LYST/CTSS/GMFG/FABP5/HLA-B/TYROBP/CORO1A/MNDA/RETN/IL1B/MMP9/FTH1/NA/MPO/CAMP/FGL2/SPON2/CADM1/PTPRC/COTL1/CYBA/NA/NA/CTSC/LTF/S100A12/CD74/LYZ/NA/NA/S100A9/NA/S100A8/NA |
| cytokine-mediated signaling pathway | -1.56614 | 0.001333 | 0.009182 | AIP/ITGB1/HSP90AA1/CCL3L1/LAMA5/PTGS2/IFI27/RHOU/IL1RAP/PSMB10/PSMD7/PDCD4/TNFRSF14/PYCARD/CAPZA1/CPNE1/PDGFB/PSMD13/PSMB8/IKBKB/HLA-C/MIF/CD4/PSMA4/SPPL2A/PSMA3/IFI35/PSMB5/P4HB/AGPAT1/TGFB1/IRF7/IFNAR2/TNFRSF1A/COL1A2/SIGIRR/HLA-A/PSMB3/TP53/AKT1/AGPAT2/PSMA1/WNT5A/RBX1/IL7R/TNFRSF19/SLC27A1/CFL1/SOCS3/ISG20/HSPA1A/COMMD7/CARD8/PSME2/GBP1/IFNGR2/CARD16/CXCR4/CNN2/ISG15/IL11RA/F3/NCAM1/PSMB9/BIRC3/LIMS1/TALDO1/MX2/SPI1/SAMHD1/SOCS2/IFITM1/LCN2/ACTN4/HLA-DQA1/B2M/BST2/CSF3R/SOX2/FCER1G/CXCL12/HLA-B/TNFAIP3/CXCL8/PTPRZ1/IL1B/MMP9/LCP1/IFITM3/PTPRC/HLA-DPB1/HLA-DPA1/IFITM2/CCL2/CCL19/HLA-DRB1/HLA-DRA/CD74/NA/NA |
| import into cell | -1.55949 | 0.001333 | 0.009182 | PTPRJ/SLC9A3/INPPL1/PTEN/WIPF1/C9orf72/EFNB2/SH3BP1/MYO10/RAB34/CTTN/ARPC1A/BIN3/BRK1/SYT11/ACTR2/LOXL3/ATG5/CALCRL/SCRIB/ARPC2/HYOU1/UNC119/SBSPON/GRK2/PSEN1/BIN1/FNBP1/SDCBP/ITGB1/GAK/HSP90AA1/GPC3/ARF6/M6PR/SH3KBP1/WIPF2/RHOU/ARPC5/ANKRD13D/SLC12A7/SLC22A3/MYO1C/RAMP1/CAP1/SNX17/LMAN2/ARRB2/PYCARD/CD81/CD63/SLC12A2/CSK/CALM3/ATP1A2/ICAM3/LRRK2/NME1/PTK2/AAK1/ENPP2/TGFB1/CSNK1E/VPS28/AP2M1/NLGN4X/WNT5A/CXCL16/IGF2R/PPT1/ACTB/NA/PTX3/PEAR1/FCGR2B/RARA/AP2S1/ABCA1/ARPC3/TINAGL1/HSPH1/ATP1B1/PECAM1/SRPX/NCF2/ELANE/PDLIM7/APOC1/CDC42SE1/ACTN4/B2M/JCHAIN/FCER1G/SNCA/HSPG2/TYROBP/CORO1A/XKR4/AIF1/CXCL8/IL1B/NA/SPON2/PTPRC/ARPC1B/LGALS3BP/CYBA/NA/NA/PRNP/CCL2/SPARC/CCL19/GPM6B/APOE/NA/NA/NA/NA |
| myeloid leukocyte activation | -1.77575 | 0.001335 | 0.009182 | IL4R/PRDX6/PYGL/NHLRC3/C1orf35/SPHK1/ANPEP/DYNLT1/NCSTN/MVP/PGRMC1/PSMD3/NAPRT/IFNGR1/TGFBR2/FES/RAB27A/PTPRJ/CRTC3/ADAM10/NR4A3/GOLGA7/DEGS1/SNAP23/ARL8A/GYG1/PFKL/PLEKHO2/SERPINB1/CRISPLD2/SLC2A3/ACTR2/UBR4/TOLLIP/PLAU/PSMB1/PTGES2/PSEN1/GPI/ACAA1/SDCBP/HSP90AA1/HEBP2/PRCP/PGAM1/ARPC5/VAMP2/HGSNAT/HYAL2/PSMD7/CAP1/CPPED1/HSPA6/APRT/PYCARD/SLC44A2/CD63/TMC6/ALDH3B1/CPNE1/PSMD13/LRRK2/MIF/NECTIN2/TGFB1/OSTF1/DGAT1/RHOG/AGPAT2/STK10/NDUFC2/C1QA/WNT5A/CLEC12A/IQGAP2/PLAUR/GAA/BRI3/IGF2R/QPCT/LAMTOR1/HSPA1A/VAMP8/TRAPPC1/STXBP2/SVIP/PTX3/FCGR2B/CNN2/NAMPT/TSPAN14/VCL/ASAH1/PECAM1/S100P/SPI1/CYBB/CTSH/PGLYRP1/GCA/RNASET2/FOLR3/ELANE/A1BG/LCN2/GRN/B2M/RAC2/BST2/SYNGR1/CD59/CTSG/FCER1G/CTSD/SNCA/CTSS/GMFG/FABP5/TYROBP/AIF1/CXCL8/MNDA/RETN/MMP9/FTH1/MPO/CAMP/FGL2/PTPRC/COTL1/CYBA/CTSC/LTF/S100A12/CD74/LYZ/S100A9/S100A8 |
| endocytosis | -1.58737 | 0.001337 | 0.009182 | PTPRJ/INPPL1/PTEN/WIPF1/C9orf72/EFNB2/SH3BP1/MYO10/RAB34/CTTN/ARPC1A/BIN3/BRK1/SYT11/ACTR2/LOXL3/ATG5/CALCRL/SCRIB/ARPC2/HYOU1/UNC119/SBSPON/GRK2/BIN1/FNBP1/SDCBP/ITGB1/GAK/HSP90AA1/GPC3/ARF6/M6PR/SH3KBP1/WIPF2/RHOU/ARPC5/ANKRD13D/MYO1C/RAMP1/CAP1/SNX17/LMAN2/ARRB2/PYCARD/CD81/CD63/CSK/CALM3/ICAM3/LRRK2/NME1/PTK2/AAK1/ENPP2/TGFB1/CSNK1E/VPS28/AP2M1/NLGN4X/WNT5A/CXCL16/IGF2R/PPT1/ACTB/NA/PTX3/PEAR1/FCGR2B/RARA/AP2S1/ABCA1/ARPC3/TINAGL1/HSPH1/PECAM1/SRPX/NCF2/ELANE/PDLIM7/APOC1/CDC42SE1/ACTN4/B2M/JCHAIN/FCER1G/SNCA/HSPG2/TYROBP/CORO1A/XKR4/AIF1/CXCL8/IL1B/NA/SPON2/PTPRC/ARPC1B/LGALS3BP/CYBA/NA/NA/CCL2/SPARC/CCL19/APOE/NA/NA/NA/NA |
| leukocyte activation involved in immune response | -1.75605 | 0.001339 | 0.009182 | ADAM10/NR4A3/GOLGA7/DEGS1/SNAP23/ARL8A/GYG1/PFKL/PLEKHO2/SERPINB1/CRISPLD2/SLC2A3/ACTR2/UBR4/LOXL3/TOLLIP/PLAU/PSMB1/PTGES2/MAD2L2/PSEN1/GPI/ACAA1/SDCBP/HSP90AA1/HEBP2/PRCP/PGAM1/ARPC5/ERCC1/VAMP2/HGSNAT/PSMD7/CAP1/CPPED1/HSPA6/APRT/PYCARD/CD81/SLC44A2/CD63/TMC6/ALDH3B1/CPNE1/PSMD13/MIF/TGFB1/OSTF1/DGAT1/TP53/RHOG/AGPAT2/STK10/NDUFC2/CLEC12A/IQGAP2/PLAUR/GAA/BRI3/IGF2R/QPCT/LAMTOR1/HSPA1A/VAMP8/TRAPPC1/STXBP2/SVIP/PTX3/FCGR2B/CNN2/RARA/TSPAN14/VCL/LGALS1/ASAH1/PECAM1/S100P/CYBB/CTSH/MDK/PGLYRP1/GCA/RNASET2/FOLR3/ELANE/A1BG/LCN2/GRN/B2M/RAC2/BST2/SYNGR1/CD59/CTSG/FCER1G/CTSD/CTSS/GMFG/FABP5/TYROBP/CORO1A/MNDA/RETN/MMP9/LCP1/FTH1/MPO/CAMP/FGL2/PTPRC/COTL1/CYBA/CTSC/LTF/S100A12/CCL19/LYZ/S100A9/S100A8 |
| positive regulation of immune response | -1.91211 | 0.00134 | 0.009182 | WIPF2/ARPC5/CD276/PSMB10/MYO1C/IFI16/PSMD7/NOP53/TNFRSF14/PAG1/ARRB2/PYCARD/CD81/C1RL/CSK/PSMD13/ICAM3/PSMB8/C1S/IKBKB/HLA-C/MIF/CD4/PSMA4/PSMA3/CNPY3/PSMB5/NECTIN2/PTK2/MUCL1/TGFB1/IRF7/PSPC1/C1QBP/HLA-A/PSMB3/PSMA1/C1QA/WNT5A/ACTB/NA/HSPA1A/VAMP8/PSME2/FCGR2B/GBP1/RARA/C1R/ARPC3/PSMB9/BIRC3/HMGB2/COL20A1/PGLYRP1/ELANE/FYB1/HLA-DQA1/B2M/CD59/CTSG/FCER1G/MEF2C/A2M/CTSS/HLA-B/THY1/TNFAIP3/MNDA/IL1B/NA/SPON2/CADM1/PTPRC/HLA-DPB1/ARPC1B/CYBA/NA/NA/PRNP/LTF/HLA-DPA1/CCL19/HLA-DRB1/HLA-DRA/CD74/NA/NA/S100A9/NA/S100A8/NA |

**Table S7 Pathway enrichment analysis of macrophages in the ligament tissue.**

| GO term | pvalue | qvalue | Gene ID |
| --- | --- | --- | --- |
| GOBP_RESPONSE_TO_BACTERIUM | 8.57E-07 | 0.000163 | SOD2/ABCA1/THBD/CXCL8/RGS1/NFKBIA/IGLC2/IGHG1/JUND/IGHG4/IL10/TNFAIP3/ZFP36/IL1B/CXCL3/CXCL2/IGHA1/IGKC |
| GOBP_REGULATION_OF_B_CELL_ACTIVATION | 1.04E-06 | 0.000163 | IGLC2/IGHG1/IGHG4/IL10/TFRC/TNFAIP3/GPR183/IGHA1/IGKC |
| GOBP_B_CELL_ACTIVATION | 1.79E-05 | 0.001877 | IGLC2/IGHG1/IGHG4/IL10/TFRC/TNFAIP3/GPR183/IGHA1/IGKC |
| GOBP_ADAPTIVE_IMMUNE_RESPONSE | 3.92E-05 | 0.003084 | IGLC2/IGHG1/IGHG4/IL10/TFRC/TNFAIP3/IL1B/GPR183/IGHA1/IGKC |
| GOBP_PURINE_CONTAINING_COMPOUND_METABOLIC_PROCESS | 9.67E-05 | 0.003335 | NUPR1/TREM2/ATP5PF/PKM/ATP5MC3/TSPO/GAPDH/MPC2/TPI1/ENO1 |
| GOBP_HUMORAL_IMMUNE_RESPONSE | 6.49E-05 | 0.003335 | CXCL8/IGLC2/IGHG1/IGHG4/IL1B/GPR183/CXCL3/CXCL2/IGHA1/IGKC |
| GOBP_ADAPTIVE_IMMUNE_RESPONSE_BASED_ON_SOMATIC_RECOMBINATION_OF_IMMUNE_RECEPTORS_BUILT_FROM_IMMUNOGLOBULIN_SUPERFAMILY_DOMAINS | 9.96E-05 | 0.003335 | IGLC2/IGHG1/IGHG4/IL10/TFRC/TNFAIP3/IL1B/IGHA1/IGKC |
| GOBP_REGULATION_OF_LYMPHOCYTE_ACTIVATION | 8.63E-05 | 0.003335 | IGLC2/IGHG1/IGHG4/IL10/TFRC/TNFAIP3/IL1B/GPR183/IGHA1/IGKC |
| GOBP_DEFENSE_RESPONSE_TO_OTHER_ORGANISM | 0.000106 | 0.003335 | CCL3L1/ITGAX/CXCL8/IGLC2/IGHG1/IGHG4/IL10/TNFAIP3/IL1B/CXCL3/CXCL2/IGHA1/IGKC |
| GOBP_BIOLOGICAL_PROCESS_INVOLVED_IN_INTERSPECIES_INTERACTION_BETWEEN_ORGANISMS | 6.93E-05 | 0.003335 | CCL3L1/ITGAX/CXCL8/RGS1/NFKBIA/IGLC2/IGHG1/JUND/IGHG4/IL10/TFRC/TNFAIP3/ZFP36/IL1B/CXCL3/CXCL2/IGHA1/IGKC |
| GOBP_POSITIVE_REGULATION_OF_CELL_ACTIVATION | 0.000122 | 0.003479 | IGLC2/IGHG1/JUND/IGHG4/IL10/TFRC/IL1B/GPR183/IGHA1/IGKC |
| GOBP_IMMUNE_RESPONSE | 0.000245 | 0.006416 | CCL3L1/CXCL8/RGS1/NFKBIA/IGLC2/IGHG1/IGHG4/IL10/TFRC/TNFAIP3/IL1B/GPR183/CXCL3/CXCL2/IGHA1/IGKC |
| GOBP_NUCLEOBASE_CONTAINING_SMALL_MOLECULE_METABOLIC_PROCESS | 0.00039 | 0.007215 | NUPR1/TREM2/ATP5PF/PKM/ATP5MC3/TSPO/PARK7/GAPDH/MPC2/TPI1/ENO1 |
| GOBP_ATP_METABOLIC_PROCESS | 0.000387 | 0.007215 | NUPR1/TREM2/ATP5PF/PKM/ATP5MC3/TSPO/GAPDH/TPI1/ENO1 |
| GOBP_NUCLEOSIDE_TRIPHOSPHATE_METABOLIC_PROCESS | 0.000387 | 0.007215 | NUPR1/TREM2/ATP5PF/PKM/ATP5MC3/TSPO/GAPDH/TPI1/ENO1 |
| GOBP_LYMPHOCYTE_MEDIATED_IMMUNITY | 0.000323 | 0.007215 | IGLC2/IGHG1/IGHG4/IL10/TFRC/IL1B/IGHA1/IGKC |
| GOBP_B_CELL_MEDIATED_IMMUNITY | 0.000311 | 0.007215 | IGLC2/IGHG1/IGHG4/IL10/TFRC/IGHA1/IGKC |
| GOBP_IMMUNE_RESPONSE_REGULATING_SIGNALING_PATHWAY | 0.000489 | 0.008173 | NFKBIA/IGLC2/IGHG1/IGHG4/TNFAIP3/IGHA1/IGKC |
| GOBP_REGULATION_OF_CELL_ACTIVATION | 0.000493 | 0.008173 | IGLC2/IGHG1/JUND/IGHG4/IL10/TFRC/TNFAIP3/IL1B/GPR183/IGHA1/IGKC |
| GOBP_LYMPHOCYTE_ACTIVATION | 0.000531 | 0.008351 | IGLC2/IGHG1/IGHG4/IL10/TFRC/TNFAIP3/IL1B/GPR183/IGHA1/IGKC |
| GOBP_POSITIVE_REGULATION_OF_IMMUNE_RESPONSE | 0.000568 | 0.008507 | IGLC2/IGHG1/IGHG4/IL10/TFRC/IL1B/IGHA1/IGKC |
| GOBP_LEUKOCYTE_MEDIATED_IMMUNITY | 0.000595 | 0.008514 | IGLC2/IGHG1/IGHG4/IL10/TFRC/IL1B/IGHA1/IGKC |
| GOBP_RESPONSE_TO_MOLECULE_OF_BACTERIAL_ORIGIN | 0.000722 | 0.009538 | SOD2/ABCA1/THBD/CXCL8/NFKBIA/JUND/IL10/TNFAIP3/ZFP36/IL1B/CXCL3/CXCL2 |
| GOBP_IMMUNE_EFFECTOR_PROCESS | 0.000727 | 0.009538 | IGLC2/IGHG1/IGHG4/IL10/TFRC/IL1B/GPR183/IGHA1/IGKC |
| GOBP_CARBOHYDRATE_DERIVATIVE_METABOLIC_PROCESS | 0.000841 | 0.01018 | NUPR1/TREM2/ATP5PF/PKM/ATP5MC3/TSPO/AKR1A1/PARK7/GAPDH/MPC2/TPI1/ENO1 |
| GOBP_DEFENSE_RESPONSE_TO_BACTERIUM | 0.000822 | 0.01018 | IGLC2/IGHG1/IGHG4/IL10/IL1B/IGHA1/IGKC |
| GOBP_CELL_CHEMOTAXIS | 0.000954 | 0.010725 | VEGFA/CCL3L1/CXCL8/IL10/IL1B/GPR183/CXCL3/CXCL2 |
| GOBP_LEUKOCYTE_CHEMOTAXIS | 0.000954 | 0.010725 | VEGFA/CCL3L1/CXCL8/IL10/IL1B/GPR183/CXCL3/CXCL2 |
| GOBP_ORGANOPHOSPHATE_METABOLIC_PROCESS | 0.001056 | 0.011287 | NUPR1/TREM2/ATP5PF/PKM/ATP5MC3/TSPO/AKR1A1/PARK7/GAPDH/MPC2/TPI1/ENO1 |
| GOBP_REGULATION_OF_IMMUNE_RESPONSE | 0.001076 | 0.011287 | NFKBIA/IGLC2/IGHG1/IGHG4/IL10/TFRC/TNFAIP3/IL1B/IGHA1/IGKC |
| GOBP_REGULATION_OF_IMMUNE_SYSTEM_PROCESS | 0.001405 | 0.01426 | CXCL8/NFKBIA/IGLC2/IGHG1/JUND/IGHG4/IL10/TFRC/TNFAIP3/ZFP36/IL1B/GPR183/IGHA1/IGKC |
| GOBP_ACTIVATION_OF_IMMUNE_RESPONSE | 0.001711 | 0.016829 | IGLC2/IGHG1/IGHG4/IL1B/IGHA1/IGKC |
| GOBP_PHAGOCYTOSIS | 0.002384 | 0.022735 | IGLC2/IGHG1/IGHG4/IL1B/IGHA1/IGKC |
| GOBP_DEFENSE_RESPONSE | 0.002534 | 0.02346 | CCL3L1/ITGAX/CXCL8/NFKBIA/IGLC2/IGHG1/IGHG4/SOCS3/IL10/TFRC/TNFAIP3/ZFP36/IL1B/CXCL3/CXCL2/IGHA1/IGKC |
| GOBP_TAXIS | 0.002626 | 0.023617 | VEGFA/CCL3L1/CXCL8/PLAUR/IL10/IL1B/GPR183/CXCL3/CXCL2 |
| GOBP_POSITIVE_REGULATION_OF_IMMUNE_SYSTEM_PROCESS | 0.002859 | 0.024995 | CXCL8/IGLC2/IGHG1/JUND/IGHG4/IL10/TFRC/IL1B/GPR183/IGHA1/IGKC |
| GOCC_SIDE_OF_MEMBRANE | 0.003008 | 0.025588 | ICAM1/ABCA1/THBD/ITGAX/RGS1/IGLC2/IGHG1/IGHG4/TFRC/IGHA1/IGKC |
| GOMF_SIGNALING_RECEPTOR_BINDING | 0.003235 | 0.026797 | VEGFA/CCL3L1/ITGAX/CXCL8/IGLC2/IGHG1/NAMPT/IGHG4/PLAUR/IL10/IL1B/CXCL3/CXCL2/IGHA1/IGKC |
| GOCC_EXTERNAL_SIDE_OF_PLASMA_MEMBRANE | 0.004832 | 0.038994 | ICAM1/ABCA1/THBD/ITGAX/IGLC2/IGHG1/IGHG4/TFRC/IGHA1/IGKC |
| GOBP_ION_TRANSMEMBRANE_TRANSPORT | 0.004985 | 0.039225 | HAMP/TREM2/ATP5PF/ATP5MC3/ATP6V0E1/PARK7/ATP6V1F/MPC2/CALM2/YWHAE |
